# Identification of Novel COVID-19 Biomarkers by Multiple Feature Selection Strategies

**DOI:** 10.1155/2021/2203636

**Published:** 2021-09-27

**Authors:** Shuai Zhang, Renliang Qu, Pengyan Wang, Shenghan Wang

**Affiliations:** ^1^Department of Clinical Laboratory, Yantai Qishan Hospital, Yantai, Shandong Province, China 264000; ^2^Department of Microbiological Laboratory, Yantai Zhifu Center for Disease Control and Prevention, Yantai, Shandong Province, China 264000

## Abstract

Coronavirus disease 2019 (COVID-19) arising from severe acute respiratory syndrome coronavirus 2 (SARS-CoV-2) has resulted in a global pandemic since its first report in December 2019. So far, SARS-CoV-2 nucleic acid detection has been deemed as the golden standard of COVID-19 diagnosis. However, this detection method often leads to false negatives, thus triggering missed COVID-19 diagnosis. Therefore, it is urgent to find new biomarkers to increase the accuracy of COVID-19 diagnosis. To explore new biomarkers of COVID-19 in this study, expression profiles were firstly accessed from the GEO database. On this basis, 500 feature genes were screened by the minimum-redundancy maximum-relevancy (mRMR) feature selection method. Afterwards, the incremental feature selection (IFS) method was used to choose a classifier with the best performance from different feature gene-based support vector machine (SVM) classifiers. The corresponding 66 feature genes were set as the optimal feature genes. Lastly, the optimal feature genes were subjected to GO functional enrichment analysis, principal component analysis (PCA), and protein-protein interaction (PPI) network analysis. All in all, it was posited that the 66 feature genes could effectively classify positive and negative COVID-19 and work as new biomarkers of the disease.

## 1. Introduction

Severe acute respiratory syndrome coronavirus 2 (SARS-CoV-2) is a positive-sense single-stranded RNA virus (+ssRNA virus) that triggered the global coronavirus disease 2019 (COVID-19) epidemic [1]. Most *α* and *β* coronaviruses can cause mild flu-like symptoms, while SARS-CoV-2 infection can lead to severe acute respiratory syndrome [2].

COVID-19 diagnosis is a vital step for antiepidemic affairs. To date, several commercialized SARS-CoV-2 detection kits have acquired the Emergency Use Authorization (EUA) of the Food and Drug Administration (FDA), such as qRT-PCR assay (detection of specific sequences of the virus), antibody assay (detection of serum antiviral antibodies IgG and IgM), and lateral flow assay (detection of viral antigens). qRT-PCR assay is well-accepted as the most reliable method and serves as the golden standard for COVID-19 diagnosis. However, the assay is not perfect. Sensitivity of qRT-PCR often relies on the virus load in samples, thus easily causing false negative results. For instance, inappropriate preservation of samples causes virus RNA degradation; inappropriate sample collection results in insufficient virus RNA; or the virus load is insufficient in samples of patients in the early stage of SARS-CoV-2 infection [1]. Therefore, it is urgent to come up with a novel COVID-19 diagnostic method to increase specificity and sensitivity. Due to various complex biological reactions occurring in the patient's infection site during SARS-CoV-2 infection, it could be a novel idea for COVID-19 diagnosis to detect several key genes in samples meanwhile combining the expression of diversified genes.

Machine learning can predict unknown data based on known data and has been widely applied in the life science field [3]. Support vector machine (SVM), as a machine learning method, determines the patient's prognosis, drug efficacy, and tumor classification based on a known gene expression profile [4]. The basic principle of the algorithm is to create the decision boundary from the known data and classify the unknown data based on the decision boundary. In 2017, Xu et al. [5] established a 15-gene-based classifier and effectively predicted postoperative occurrence of colon cancer using the classifier. Altogether, SVM is a highly efficient bioinformatics method for classification. In this study, bioinformatics analysis was applied to mine main feature genes from expression profiles of COVID-19 positive and negative samples. The expression profile genes were ranked by feature importance via the minimum-redundancy maximum-relevancy (mRMR) method. SVM classifiers of different feature gene sets were constructed, and 66 optimal feature genes were screened by the incremental feature selection (IFS) method. Finally, principal component analysis (PCA) and functional enrichment analysis were used to determine whether these feature genes could be used as novel biomarkers of COVID-19.

## 2. Materials and Methods

### 2.1. Expression Profiles and Research Design

**Figure 1:**
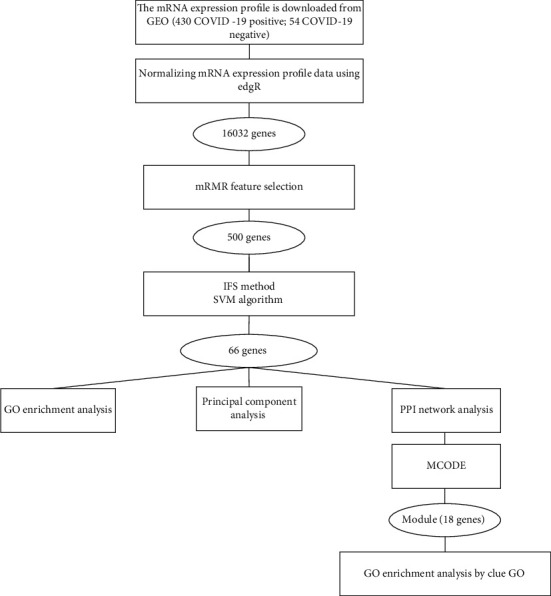
Flow chart of bioinformatics analysis.

In the present study, the expression profile (GSE152075) was downloaded from the Gene Expression Omnibus (GEO) database (https://www.ncbi.nlm.nih.gov/geo/). The expression data was obtained through GPL18573 Illumina NextSeq 500 (Homo sapiens) platform sequencing, including mRNA sequencing results of throat swab samples of 54 negative and 430 positive COVID-19. In the expression matrix, genes with average value < 1 and maximum value < 5 were deleted. The other genes were standardized by using the edgeR package, and 16,032 genes were obtained [6] (Supplementary Table 1). A bioinformatics analysis flow chart was designed as follows in Figure 1.

### 2.2. Feature Gene Selection

Feature genes were ranked with the mRMR method. mRMR acquires feature values by computing max relevance and minimal redundancy [7]. Max relevance met the following formula:

(1) $$\begin{array}{c} \max D\left(S,c\right),D=\frac{1}{\left|S\right|}\underset{x_{i}\in S}{\sum }I\left(x_{I};c\right). \end{array}$$

High redundancy may exist in the selected genes according to the max relevance. Thus, removal of a feature would not have much influence on the classification results. To further screen relatively independent features, minimal redundancy was included into the feature value algorithm. The minimal redundancy met the following formula:

(2) $$\begin{array}{c} \min R\left(S\right),R=\frac{1}{{\left|S\right|}^{2}}\underset{x_{i},x_{j}\in S}{\sum }I\left(x_{i},x_{j}\right). \end{array}$$

In the above formulas (1) and (2), *S* is the feature set, *x* is the feature, and *c* is the classification.

An algorithm which combined max relevance and minimal redundancy was named mRMR and was defined as

(3) $$\begin{array}{c} \max\Phi \left(D,R\right),\;\Phi =D-R. \end{array}$$

The mRMR algorithm routine was downloaded from website (http://home.penglab.com/proj/mRMR/). Feature genes in the expression profiles were scored with the downloaded routine and ranked by the score.

### 2.3. Screening of Optimal Feature Genes

IFS was performed to further select optimal feature genes from [8]. Feature set *F* (*F* = [*f*_1_, *f*_2_, *f*_3_, ⋯., *f*_*N*_], *N* ranged from 1 to 500) was first constructed. Afterwards, the corresponding SVM classifier for each subset was constructed based on *F* using python package *sklearn*. SVM is an effective method for classifier construction [4]. The specific method is to create a decision boundary between two types in order to predict the type of input samples. Decision boundary, or the hyperplane, is a definition away from the nearest data sites (called support vectors) in each class as much as possible. The specific algorithm was shown as follows:

(4) $$\begin{array}{c} \left(x_{1},y_{1}\right),\cdots ,\left(x_{n},y_{n}\right),\;x_{i}\in R^{d},\;y_{1}\in \left(-1,+1\right). \end{array}$$

*x*_*i*_ is the feature vector and *y*_*i*_ is the class in the train set (negative or positive). The optimal hyperplane was defined as follows:

(5) $$\begin{array}{c} wx^{T}+b=0. \end{array}$$

*w* is the weight vector, *x* is the input feature vector, and *b* is the deviation. Both *w* and *b* met the following conditions:

(6) $$\begin{array}{c} \begin{array}{cc} wx_{i}^{T}+b\geq +1, & \text{ⅈf }y_{i}=+1,\\ w_{x_{i}}^{T}+b\leq -1, & \text{ⅈf }y_{i}=-1. \end{array} \end{array}$$

*w* and *b* were determined by inputting feature vectors and classes in the training set to classify the prediction set. Due to sample imbalance, the python package *imblearn* was used to amplify the number of small samples to the same as that of large samples [9]. Different feature sets were taken as the training set. Model training was undertaken to construct a SVM classifier for each set. The performance of the established classifiers was evaluated by leave-one-out cross-validation (LOOCV) and presented by the Matthews correlation coefficient (MCC). MCC is a Pearson correlation coefficient of the actual value and the predicted value computed by the confusion matrix method. The MCC value is between -1 and +1. The MCC value close to +1 means accurate prediction, close to 0 means no better than random prediction, and close to -1 means disagreement between prediction and actual observation [10]. A series of MCC values corresponding to different feature sets were obtained through IFS. The IFS curve was drawn with MCC value as the *y*-axis and the feature set as the *x*-axis. The training set with the highest MCC value in the IFS curve was chosen, and genes in this set were set as the optimal feature genes.

### 2.4. PCA

PCA is mainly applied for exploratory spatial data analysis and prediction model construction [11]. This method can reduce the dimension of high-latitude data. Simply, each data site was mapped to the latter principal components, and different levels of each data site were preserved as much as possible. In the present study, the first and second principal components of optimal feature genes were the R package *FactoMineR* [12]. Expression data of high-latitude feature genes based on the two dimensionalities were mapped to a two-dimensional plane consisting of PC1 and PC2.

### 2.5. Enrichment Analysis

Gene Ontology (GO) enrichment analysis was performed on the optimal feature genes screened by IFS by using the R package *clusterProfiler* [13]. The classification results were presented by biological process (BP), cellular component (CC), and molecular function (MF).

### 2.6. Protein-Protein Interaction (PPI) Network Analysis

PPI network analysis (minimum required interaction score = 0.7; others were default parameters) was conducted on optimal feature genes screened by IFS using the STRING (https://www.string-db.org/) database [14]. The set with the highest connectivity in the PPI network (main set) was found using the MCODE plug-in in Cytoscape. GO enrichment analysis was conducted on the main set in the PPI network by using GlueGO plug-in in Cytoscape.

## 3. Results

### 3.1. Feature Gene Selection

Gene expression profiles of negative and positive COVID-19 throat swabs were accessed from the GEO database. Altogether, 16,032 genes were acquired by standardization (Supplementary Table 1). To excavate novel biomarkers of COVID-19 from these genes, expression profile genes were ranked in terms of the expression feature by mRMR. Thereafter, the top 500 ranked genes (Supplementary Table 2) were used for the subsequent screening for optimal feature genes.

### 3.2. Screening Optimal Feature Genes and Enrichment Analysis

**Figure 2:**
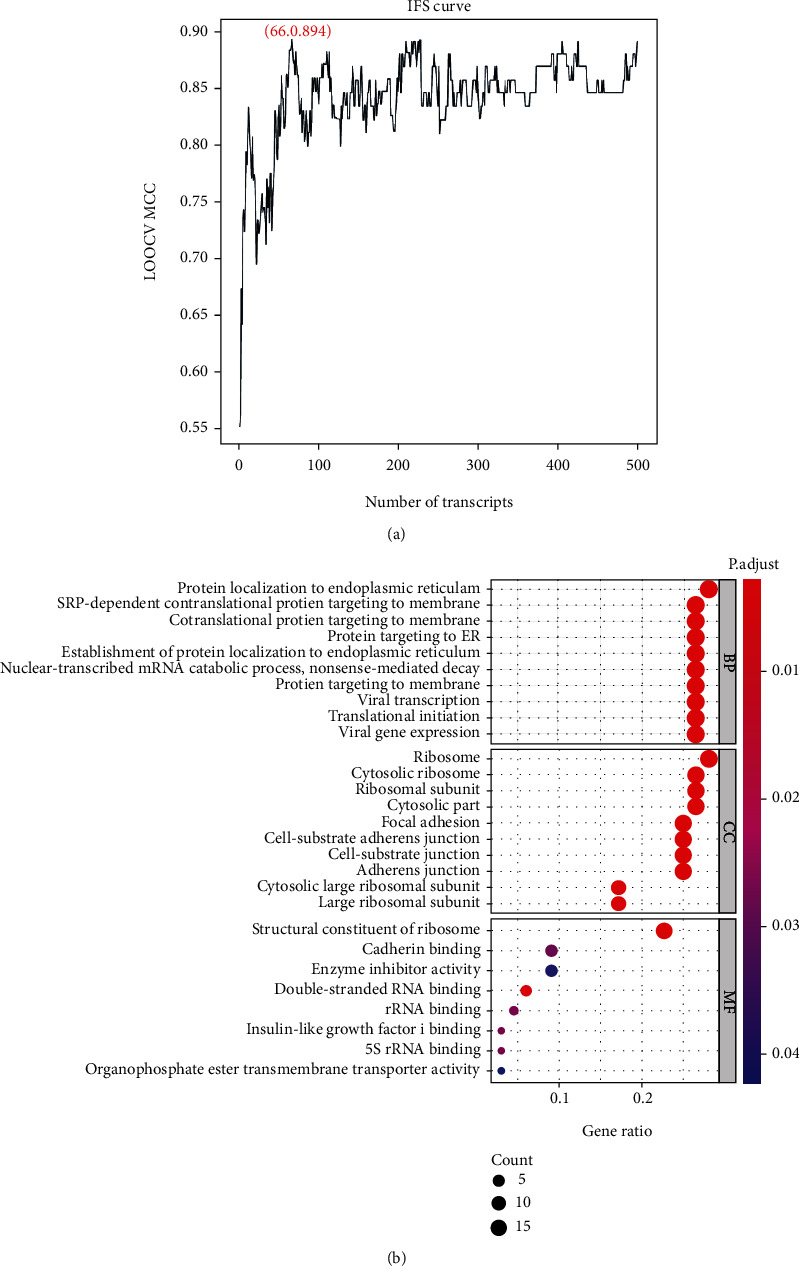
Screening of optimal feature genes. (a) IFS evaluated the performance of SVM classifiers based on different groups of feature genes. *x*-axis: feature gene number; *y*-axis: MCC value. (b) Bubble plot of GO enrichment analysis of optimal feature genes. The classification results included BP, CC, and CF.

Optimal feature genes were determined by the IFS method. Feature set *F* (*F* = [*f*_1_, *f*_2_, *f*_3_, ⋯., *f*_*N*_], *N* ranged from 1 to 500) was constructed with the 500 screened feature genes, and a SVM classifier corresponding to each set was also built. An IFS curve was drawn with the MCC value of the SVM classifier as the *y*-axis and the feature gene number as the *x*-axis (Figure 2(a)). According to the IFS curve, the MCC value of the top 66 feature genes (Supplementary Table 3) was taken as the training set. The classification effects of top 66 feature gene-based SVM classifier were presented as the MCC value: 0.894, sensitivity: 0.991, specificity: 0.889, and accuracy: 0.979. Thus, the top 66 feature genes were set as the optimal feature genes. Next, GO enrichment analysis was performed on the top 66 feature genes. The results were shown as follows: in the BP module, these genes were mainly enriched in protein localization to endoplasmic reticulum, SRP-dependent cotranslational protein targeting to membrane, and cotranslational protein targeting to membrane. In the CC module, these genes were mainly enriched in the cytosolic ribosome, ribosomal subunit, and ribosome. In the MF module, these genes were mainly enriched in the structural constituent of the ribosome (Figure 2(b)). Enrichment analysis exhibited that these feature genes were mostly relevant to ribosomal protein, protein secretion, and membrane location.

### 3.3. PCA

**Figure 3:**
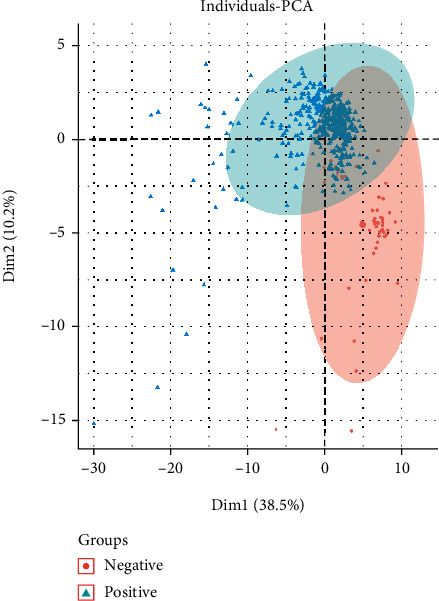
PCA. PCA of the optimal feature genes. Green triangles refer to positive samples. Red circles refer to negative samples.

PCA was conducted on samples to testify whether optimal feature genes can effectively classify negative and positive samples. Evident separation was found between positive sample clusters (green triangle) and negative sample clusters (red circle) in the two-dimensional plane consisting of PC1 and PC2 (Figure 3). It was illuminated that the optimal feature genes could effectively distinguish positive and negative COVID-19.

### 3.4. PPI Network Analysis

**Figure 4:**
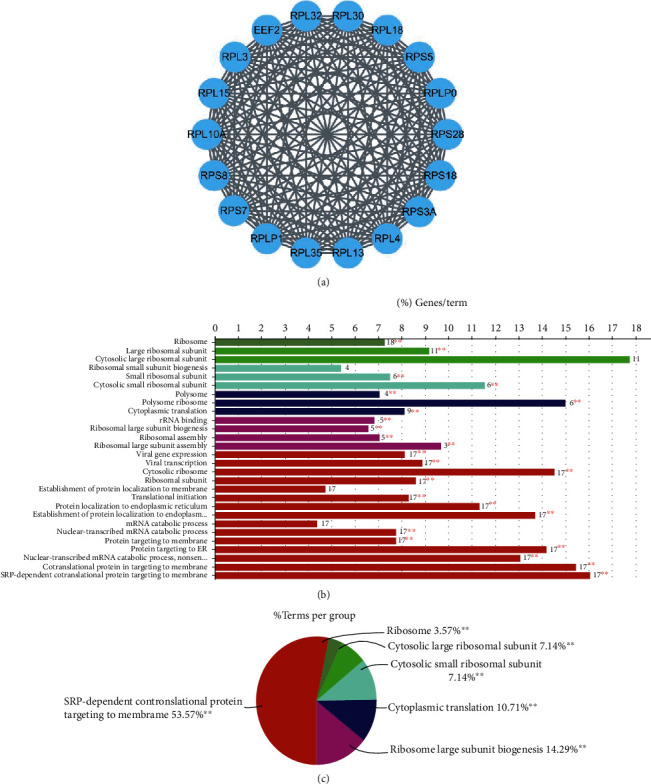
PPI network analysis. (a) Main set was selected from PPI network based on optimal feature genes by using MCODE. (b–c) Enrichment analysis was performed on selected main sets with ClueGO. ^∗∗^*p* < 0.01.

To explore the interaction between optimal feature genes, PPI network analysis was undertaken on the STRING (https://www.string-db.org/) database. The maximum set in the PPI network constructed was chosen using the MCODE plug-in (18 nodes, 153 lines) (Figure 4(a)). To further explore enriched biological functions by feature genes in the chosen set, GO analysis was performed on 18 genes in the set. It was discovered that these genes were mainly enriched in the cytosolic large ribosomal subunit, polysomal ribosome, viral gene expression, and SRP-dependent cotranslational protein targeting to the membrane. This indicated that these genes were associated with ribosomal protein-encoding, viral protein translation, and protein-membrane location (Figures 4(b) and 4(c)).

## 4. Discussion

In the present study, the mRMR feature selection method had been applied to screen the top 500 feature genes. The top 66 optimal feature genes were screened through the IFS method and worked as biomarkers of COVID-19. Combined with traditional diagnosis approaches, a relatively novel one was raised here. The traditional approaches usually detect SARS-CoV-2 nucleic acid, antigens, and antibodies. Notwithstanding, we provided a group of specifically expressed genes in human infected parts during SARS-CoV-2 infection, and these genes were taken as biomarkers of COVID-19. The differences between this diagnosis method and traditional ones were (I) novel COVID-19 markers were human genes and (II) this diagnosis method distinguished negative or positive samples through detecting several genes, while traditional means only detected a single nucleic acid fragment or antibodies of SARS-CoV-2. The following was a discussion of optimal feature genes.

*OAS2* is the top-ranked feature gene. *OAS2* belongs to the human 2′-5′-oligoadenylate synthetase family, which participates in nonspecific immunity during viral infection through interferon induction and degrades viral RNA [15]. Meanwhile, *OAS2* was reported to be highly expressed in positive COVID-19 patients and acts as a candidate drug target for COVID-19 treatment [16–18]. According to references and our bioinformatics analysis, it was speculated that *OAS2* may play an important role in SARS-CoV-2 infection. *RPLP0* and *RPL15* are also top-ranked optimal feature genes. They encode different ribosomal proteins *in vivo* to participate in synthesizing 60S and 40S ribosomal subunits. Ribosomes *in vivo* consist of a small 40S subunit, a large 60S subunit, and some ribosome RNAs, with different ribosomal proteins composing the two subunits [19]. Besides *RPLP0* and *RPL15*, there are still other ribosomal protein-encoding genes (*RPLP1*, *RPL10A*, *RPL3*, *RPL30*, *RPL13*, *RPL4*, *RPL18*, *RPL32*, and *RPL35*) that all participate in synthesizing ribosomal subunits. Several studies suggested that nonstructural proteins of SARS-CoV-2 (such as *NSP1*, *NSP16*, *NSP8*, and *NSP9*) bind ribosomal subunits or ribosomal RNA to inhibit nonspecific immunity and mRNA translation relevant to interferon secreting [2, 20, 21]. Thus, it was speculated that SARS-CoV-2 infection may largely affect the expression of ribosome-related genes to interfere with the translation and secretion of proteins related to human immune functions. It could be seen that these feature genes were associated with biological functions during SARS-CoV-2 infection, demonstrating that our screened feature genes were suitable for working as biomarkers of COVID-19.

GO enrichment analysis on the 66 feature genes and main set in the corresponding PPI network showed that these genes were mainly enriched in the large ribosomal subunit and SRP-dependent cotranslational protein targeting to membrane. SARS-CoV-2 may affect ribosome synthesis, protein translation, and protein secretion of cells in the patient's affected part on the transcriptome level. Meanwhile, the analysis results were consistent with what Banerjee et al. published in Cell Journal in November 2020 [20]. The study suggested that *NSP8* and *NSP9* proteins of SARS-CoV-2 bind signaling recognition particles (SRP) of the ribosomal large subunit and suppress the protein attaching cell membrane. Moreover, feature genes were mainly enriched in SRP-dependent cotranslational protein targeting to the membrane and large ribosomal subunit.

In particular, many studies have adopted analysis methods similar to this study. For example, Cheng et al. [22] in 2020 screened 31 possible markers using mRMR based on single-cell RNA sequencing data from malignant glioma and used SVM to testify the diagnostic performance of the 31 genes. Xu et al. [23] built a predictive model for preoperative lymph node status evaluation in intrahepatic cholangiocarcinoma using mRMR and SVM. It can be seen that this method can be widely used for the prediction or diagnosis of different diseases.

On the whole, 66 optimal feature genes were screened by multiple feature selection strategies. The validity of these genes as COVID-19 biomarkers was testified by enrichment analysis, PPI network analysis, and PCA. At the same time, some optimal feature genes were reported to be relevant to biological functions *in vivo* during SARS-CoV-2 infection. Hence, our results can be applied not only to the accurate diagnosis of COVID-19 but also to treatment guidance. However, limitations still exist in this study. For instance, we did not use abundant clinical samples to testify the classification performance of the classifier. Therefore, the application value of these genes as COVID-19 biomarkers during actual diagnosis was still vague. We plan to collect numerous clinical samples and diagnose them by traditional and novel methods to compare their effects.

## Data Availability

The [data type] data used to support the findings of this study are included within the article.

## Supplementary Materials

Supplementary 1 Supplementary Table 1: acquired genes after standardization.

Supplementary 2 Supplementary Table 2: screened top 500 feature genes after the mRMR method.

Supplementary 3 Supplementary Table 3: screened top 66 feature genes by the IFS method.
