# Supplementary material for: Identification of Novel COVID-19 Biomarkers by Multiple Feature Selection Strategies
Source: Comput Math Methods Med. 2021 Sep 27;2021:2203636. doi: 10.1155/2021/2203636 (PMC8485143; doi:10.1155/2021/2203636)
Supplement: Supplementary 1 — Supplementary Table 1: acquired genes after standardization. [file 2203636.f1.pdf]

Supplementary Table S1 Acquired genes after standardization

id
A1BG
A1CF
A2M
A2ML1
A4GALT
AAAS
AACS
AADACL2
AADACP1
AADAT
AAGAB
AAK1
AAMDC
AAMP
AAR2
AARD
AARS1
AARS2
AARSD1
AASDH
AASDHPPT
AASS
AATF
AATK
ABAT
ABCA1
ABCA10
ABCA11P
ABCA12
ABCA13
ABCA2
ABCA3
ABCA4
ABCA5
ABCA6
ABCA7
ABCA8
ABCA9
ABCB1
ABCB10
ABCB11
ABCB5
ABCB6
ABCB7
ABCB8
ABCB9
ABCC1
ABCC10
ABCC11
ABCC13
ABCC2
ABCC3
ABCC4
ABCC5
ABCC6
ABCC6P1
ABCC6P2

ABCC9
ABCD1
ABCD2
ABCD3
ABCD4
ABCE1
ABCF1
ABCF2
ABCF3
ABCG1
ABCG8
ABHD1
ABHD10
ABHD11
ABHD11-AS1
ABHD12
ABHD12B
ABHD13
ABHD14A
ABHD14B
ABHD15
ABHD16A
ABHD16B
ABHD17A
ABHD17B
ABHD17C
ABHD18
ABHD2
ABHD3
ABHD4
ABHD5
ABHD6
ABHD8
ABI1
ABI2
ABI3
ABI3BP
ABITRAM
ABL1
ABL2
ABLIM1
ABLIM3
ABO
ABR
ABRACL
ABRAXAS1
ABRAXAS2
ABT1
ABTB1
ABTB2
AC000367.1
AC001226.2
AC002543.1
AC002996.1
AC003002.2
AC003005.1
AC003080.1
AC003688.2

AC004076.1
AC004805.1
AC004890.2
AC004910.1
AC004923.1
AC004951.2
AC004980.1
AC004980.6
AC005041.1
AC005077.4
AC005154.2
AC005154.5
AC005324.3
AC005324.4
AC005476.1
AC005515.1
AC005520.3
AC005531.1
AC005622.1
AC005726.1
AC005822.1
AC005832.4
AC005833.1
AC005865.1
AC006001.3
AC006030.1
AC006042.2
AC006254.1
AC006453.3
AC006486.1
AC006978.1
AC006978.2
AC007000.3
AC007040.2
AC007068.1
AC007192.1
AC007318.1
AC007325.1
AC007325.2
AC007325.4
AC007342.3
AC007424.1
AC007906.2
AC008013.2
AC008038.1
AC008072.1
AC008073.3
AC008132.1
AC008163.1
AC008267.2
AC008565.1
AC008567.1
AC008575.1
AC008695.1
AC008752.4
AC008758.1
AC008763.2
AC008770.2

AC008878.3
AC008982.1
AC008982.3
AC009053.2
AC009093.3
AC009133.6
AC009220.2
AC009237.3
AC009299.1
AC009779.4
AC009879.1
AC009927.1
AC010132.3
AC010186.2
AC010203.1
AC010240.3
AC010255.3
AC010319.1
AC010325.1
AC010326.4
AC010422.6
AC010507.1
AC010522.1
AC010615.1
AC010886.1
AC010970.1
AC011043.1
AC011295.1
AC011295.2
AC011330.1
AC011448.1
AC011479.2
AC011497.2
AC011498.4
AC011511.1
AC011511.4
AC011939.1
AC012085.1
AC012184.2
AC012254.2
AC012435.2
AC012618.3
AC012651.1
AC013394.1
AC013470.2
AC015688.4
AC015813.2
AC015871.1
AC015911.7
AC016571.1
AC016582.1
AC016582.3
AC017083.3
AC017099.1
AC018523.2
AC018552.1
AC018635.1
AC018638.4

AC018638.5
AC018644.1
AC018755.2
AC018797.2
AC019117.4
AC020779.1
AC020915.1
AC020915.5
AC022080.2
AC022149.1
AC022182.3
AC022384.1
AC022400.7
AC022415.2
AC022973.2
AC023050.5
AC023055.1
AC024293.1
AC024560.2
AC024568.1
AC025423.2
AC026150.3
AC026348.1
AC026412.1
AC026464.4
AC026464.6
AC027796.3
AC034102.2
AC034236.1
AC036176.2
AC040162.1
AC040978.1
AC044860.1
AC046176.1
AC053481.2
AC060766.1
AC063952.1
AC063976.2
AC066616.1
AC066616.2
AC067968.1
AC068279.1
AC068491.1
AC068533.4
AC068547.1
AC068580.4
AC068620.3
AC068631.3
AC068647.1
AC068896.3
AC069368.1
AC069503.2
AC073065.1
AC073107.1
AC073135.7
AC073896.1
AC074141.1
AC078819.1

AC078927.1
AC079363.1
AC079414.2
AC079416.2
AC079447.1
AC079594.2
AC079781.1
AC079880.2
AC083899.1
AC084337.1
AC084871.1
AC087163.2
AC087289.3
AC087359.1
AC087632.2
AC087633.2
AC087894.1
AC090004.1
AC090114.3
AC090151.1
AC090227.2
AC090519.1
AC090527.3
AC090607.2
AC090950.1
AC090971.4
AC091057.1
AC091167.2
AC091390.5
AC091959.3
AC092045.1
AC092053.2
AC092070.2
AC092104.1
AC092299.1
AC092329.3
AC092418.2
AC092427.1
AC092474.1
AC092656.1
AC092718.3
AC092747.2
AC092821.2
AC092835.1
AC093323.1
AC093392.1
AC093525.2
AC093668.1
AC093752.1
AC093829.1
AC093899.2
AC097374.1
AC097376.2
AC097504.1
AC097637.1
AC098650.1
AC098934.1
AC098934.2

AC099489.1
AC099811.2
AC100821.1
AC103810.1
AC104109.3
AC104162.1
AC104332.1
AC104452.1
AC104772.1
AC104966.1
AC105052.3
AC105233.2
AC106782.1
AC106795.1
AC106795.4
AC106865.1
AC106886.6
AC107081.3
AC107871.1
AC108108.1
AC109992.1
AC110079.1
AC110373.1
AC110926.1
AC110998.1
AC113208.4
AC114490.1
AC114490.2
AC114936.1
AC117529.1
AC117529.2
AC118459.2
AC118549.1
AC119396.1
AC120057.2
AC121758.2
AC122718.1
AC122718.2
AC124242.2
AC124312.1
AC124944.2
AC124947.2
AC126755.1
AC127502.1
AC127502.3
AC131392.1
AC131392.2
AC132008.2
AC132812.1
AC132825.1
AC133548.2
AC133552.1
AC134669.1
AC135352.1
AC135977.1
AC135983.3
AC137834.1
AC138207.8

AC138392.1
AC138393.1
AC138409.1
AC138409.2
AC138696.1
AC138811.2
AC138827.1
AC138827.3
AC138866.1
AC138866.2
AC138894.1
AC138932.1
AC138969.1
AC139256.2
AC139494.1
AC139495.1
AC139495.2
AC139677.2
AC139677.4
AC139769.1
AC139795.1
AC141586.1
AC145212.1
AC146944.1
AC183084.2
AC211429.2
AC211476.10
AC211476.8
AC211485.1
AC211486.1
AC211486.6
AC233702.6
AC233968.1
AC235565.1
AC240274.1
AC241952.2
AC242426.2
AC243772.3
AC243791.3
AC243807.2
AC243919.1
AC243967.1
AC244197.3
AC244260.1
AC244489.1
AC244489.3
AC244635.2
AC244636.2
AC244669.1
AC245049.2
AC245060.4
AC245100.2
AC245100.4
AC245297.1
AC245690.2
ACAA1
ACAA2
ACACA

ACACB
ACAD10
ACAD11
ACAD8
ACAD9
ACADM
ACADS
ACADSB
ACADVL
ACAN
ACAP1
ACAP2
ACAP3
ACAT1
ACAT2
ACBD3
ACBD4
ACBD5
ACBD6
ACBD7
ACCS
ACD
ACE
ACE2
ACER2
ACER3
ACIN1
ACKR2
ACKR3
ACKR4
ACKR4P1
ACLY
ACO1
ACO2
ACOD1
ACOT1
ACOT11
ACOT13
ACOT2
ACOT4
ACOT7
ACOT8
ACOT9
ACOX1
ACOX2
ACOX3
ACOXL
ACP1
ACP2
ACP3
ACP5
ACP6
ACP7
ACSBG1
ACSF2
ACSF3
ACSL1
ACSL3

ACSL4
ACSL5
ACSL6
ACSM2A
ACSM2B
ACSM3
ACSM6
ACSS1
ACSS2
ACSS3
ACTA2
ACTB
ACTG1
ACTG1P1
ACTG1P14
ACTG1P17
ACTG1P9
ACTG2
ACTL6A
ACTN1
ACTN4
ACTR10
ACTR1A
ACTR1B
ACTR2
ACTR3
ACTR3B
ACTR3C
ACTR5
ACTR6
ACTR8
ACVR1
ACVR1B
ACVR1C
ACVR2A
ACVR2B
ACVRL1
ACY1
ACYP1
ACYP2
ADA
ADA2
ADAD2
ADAL
ADAM10
ADAM12
ADAM15
ADAM17
ADAM19
ADAM1A
ADAM1B
ADAM20P1
ADAM21
ADAM22
ADAM28
ADAM29
ADAM32
ADAM3A

ADAM8
ADAM9
ADAMDEC1
ADAMTS1
ADAMTS12
ADAMTS14
ADAMTS16
ADAMTS17
ADAMTS3
ADAMTS4
ADAMTS6
ADAMTS7P1
ADAMTS7P4
ADAMTS9
ADAMTSL1
ADAMTSL3
ADAMTSL4
ADAMTSL5
ADAP1
ADAP2
ADAR
ADARB1
ADARB2
ADAT1
ADAT2
ADAT3
ADCK1
ADCK2
ADCY1
ADCY10
ADCY10P1
ADCY2
ADCY3
ADCY4
ADCY5
ADCY6
ADCY7
ADCY9
ADD1
ADD2
ADD3
ADGB
ADGRA2
ADGRA3
ADGRB2
ADGRD1
ADGRD2
ADGRE1
ADGRE2
ADGRE3
ADGRE4P
ADGRE5
ADGRF1
ADGRF2
ADGRF3
ADGRF5
ADGRG1
ADGRG2

ADGRG3
ADGRG4
ADGRG5
ADGRG6
ADGRL1
ADGRL2
ADGRL3
ADGRV1
ADH1A
ADH1B
ADH1C
ADH4
ADH5
ADH6
ADH7
ADHFE1
ADI1
ADIPOQ
ADIPOR1
ADIPOR2
ADIRF
ADK
ADM
ADM2
ADNP
ADNP2
ADO
ADORA2A
ADORA2B
ADORA3
ADPGK
ADPRH
ADPRHL1
ADPRM
ADPRS
ADRA1A
ADRA2A
ADRA2B
ADRB1
ADRB2
ADRB3
ADRM1
ADSL
ADSS1
ADSS2
ADTRP
AEBP2
AEN
AF107885.1
AFAP1
AFAP1L1
AFAP1L2
AFDN
AFF1
AFF2
AFF3
AFF4
AFG1L

AFG3L1P
AFG3L2
AFMID
AFTPH
AGA
AGAP1
AGAP12P
AGAP2
AGAP3
AGAP4
AGAP5
AGAP6
AGAP7P
AGAP9
AGBL1
AGBL2
AGBL3
AGBL4
AGBL5
AGER
AGFG1
AGFG2
AGGF1
AGK
AGL
AGMAT
AGO1
AGO2
AGO3
AGO4
AGPAT1
AGPAT2
AGPAT3
AGPAT4
AGPAT5
AGPS
AGR2
AGR3
AGRN
AGTPBP1
AGTRAP
AGXT
AHCTF1
AHCTF1P1
AHCY
AHCYL1
AHCYL2
AHDC1
AHI1
AHNAK
AHNAK2
AHR
AHRR
AHSA1
AHSA2P
AICDA
AIDA
AIDAP2

AIF1
AIF1L
AIFM1
AIFM2
AIG1
AIM2
AIMP1
AIMP2
AIP
AIPL1
AIRE
AJAP1
AJM1
AJUBA
AK1
AK2
AK3
AK4
AK5
AK6
AK7
AK8
AK9
AKAP1
AKAP10
AKAP11
AKAP12
AKAP13
AKAP14
AKAP17A
AKAP3
AKAP5
AKAP6
AKAP7
AKAP8
AKAP8L
AKAP9
AKIP1
AKIRIN1
AKIRIN2
AKNA
AKR1A1
AKR1B1
AKR1B10
AKR1B15
AKR1C1
AKR1C2
AKR1C3
AKR1D1
AKR1E2
AKR7A2
AKR7L
AKT1
AKT1S1
AKT2
AKT3
AKTIP
AL021920.2

AL022334.1
AL022578.1
AL031282.1
AL031708.1
AL031777.2
AL035411.1
AL035456.1
AL035555.2
AL035685.1
AL049697.1
AL049697.3
AL109809.1
AL109811.3
AL109827.1
AL109918.1
AL117692.1
AL121581.1
AL121594.1
AL121845.3
AL132671.2
AL132780.3
AL133216.2
AL133268.1
AL133352.1
AL136231.1
AL136295.1
AL136295.4
AL136295.5
AL136981.1
AL136981.2
AL137161.1
AL138709.1
AL138752.2
AL138787.1
AL139011.2
AL139260.3
AL139274.1
AL139300.1
AL139353.1
AL157392.5
AL157935.3
AL162615.1
AL162726.3
AL163636.2
AL353588.1
AL353625.1
AL353729.2
AL353743.1
AL353807.3
AL354822.1
AL355315.1
AL355355.1
AL355377.1
AL355916.3
AL356234.1
AL356235.1
AL356273.4
AL356432.3

AL356968.2
AL357315.2
AL359641.1
AL359736.1
AL359955.1
AL359976.1
AL365205.1
AL390334.1
AL390719.1
AL390726.4
AL390728.4
AL390879.1
AL391058.1
AL391903.1
AL391987.2
AL445665.1
AL449283.1
AL450326.2
AL450996.1
AL512378.1
AL512506.3
AL512605.1
AL513477.1
AL513523.13
AL513523.3
AL513523.7
AL589743.1
AL589993.1
AL590065.1
AL590396.2
AL591379.1
AL591438.2
AL591499.1
AL591806.4
AL591846.2
AL604028.2
AL627309.6
AL645924.2
AL662796.1
AL669831.1
AL669831.3
AL669831.4
AL671883.2
AL671986.1
AL672207.1
AL691432.1
AL731556.1
AL731892.1
AL732372.2
AL732372.3
AL773545.3
AL807752.1
AL928654.3
ALAD
ALAS1
ALAS2
ALCAM
ALDH16A1

ALDH18A1
ALDH1A1
ALDH1A2
ALDH1A3
ALDH1B1
ALDH1L1
ALDH1L2
ALDH2
ALDH3A1
ALDH3A2
ALDH3B1
ALDH3B2
ALDH4A1
ALDH5A1
ALDH6A1
ALDH7A1
ALDH8A1
ALDH9A1
ALDOA
ALDOC
ALG1
ALG10
ALG10B
ALG11
ALG12
ALG13
ALG14
ALG1L
ALG2
ALG3
ALG5
ALG6
ALG8
ALG9
ALKBH1
ALKBH2
ALKBH3
ALKBH4
ALKBH5
ALKBH6
ALKBH7
ALKBH8
ALMS1
ALMS1P1
ALOX12
ALOX12P2
ALOX15
ALOX15B
ALOX15P1
ALOX5
ALOX5AP
ALPK1
ALPK2
ALPK3
ALPL
ALS2
ALS2CL
ALYREF

AMACR
AMBRA1
AMD1
AMD1P4
AMDHD1
AMDHD2
AMER1
AMER2
AMFR
AMIGO1
AMIGO2
AMIGO3
AMMECR1
AMMECR1L
AMN
AMN1
AMOT
AMOTL1
AMOTL2
AMPD2
AMPD3
AMT
AMY1A
AMY1B
AMY1C
AMY2B
AMZ1
AMZ2
AMZ2P1
AMZ2P2
ANAPC1
ANAPC10
ANAPC11
ANAPC13
ANAPC15
ANAPC16
ANAPC1P1
ANAPC1P2
ANAPC1P4
ANAPC2
ANAPC4
ANAPC5
ANAPC7
ANG
ANGEL1
ANGEL2
ANGPT1
ANGPT2
ANGPT4
ANGPTL1
ANGPTL2
ANGPTL4
ANK1
ANK2
ANK3
ANKAR
ANKDD1A
ANKDD1B

ANKEF1
ANKFN1
ANKFY1
ANKH
ANKHD1
ANKHD1-EIF4EBP3
ANKIB1
ANKK1
ANKLE2
ANKMY1
ANKMY2
ANKRA2
ANKRD10
ANKRD11
ANKRD12
ANKRD13A
ANKRD13C
ANKRD13D
ANKRD16
ANKRD17
ANKRD18A
ANKRD18B
ANKRD18CP
ANKRD18EP
ANKRD20A1
ANKRD20A11P
ANKRD20A19P
ANKRD20A21P
ANKRD20A3P
ANKRD20A4P
ANKRD20A5P
ANKRD20A8P
ANKRD22
ANKRD23
ANKRD24
ANKRD26
ANKRD27
ANKRD28
ANKRD29
ANKRD30B
ANKRD30BL
ANKRD30BP2
ANKRD31
ANKRD33B
ANKRD34A
ANKRD35
ANKRD36
ANKRD36B
ANKRD36BP1
ANKRD36BP2
ANKRD36C
ANKRD37
ANKRD39
ANKRD40
ANKRD40CL
ANKRD42
ANKRD44
ANKRD45

ANKRD46
ANKRD49
ANKRD49P1
ANKRD50
ANKRD52
ANKRD54
ANKRD6
ANKRD62
ANKRD65
ANKRD66
ANKRD9
ANKS1A
ANKS1B
ANKS3
ANKS4B
ANKS6
ANKUB1
ANKZF1
ANLN
ANO1
ANO10
ANO4
ANO6
ANO8
ANO9
ANOS1
ANP32A
ANP32B
ANP32E
ANPEP
ANTKMT
ANTXR1
ANTXR2
ANXA1
ANXA11
ANXA13
ANXA2
ANXA2P2
ANXA2R
ANXA3
ANXA4
ANXA5
ANXA6
ANXA7
ANXA8
ANXA8L1
ANXA9
AOAH
AOC1
AOC2
AOC3
AOPEP
AOX2P
AP000295.1
AP000311.1
AP000346.2
AP000347.1
AP000769.1

AP001885.1
AP001931.1
AP001992.1
AP002008.2
AP002414.2
AP002495.1
AP002748.5
AP002981.1
AP002990.1
AP003084.1
AP003108.2
AP003170.1
AP003419.1
AP004289.1
AP005061.2
AP005901.1
AP006222.1
AP006285.2
AP1AR
AP1B1
AP1G1
AP1G2
AP1M1
AP1M2
AP1S1
AP1S2
AP1S3
AP2A1
AP2A2
AP2B1
AP2M1
AP2S1
AP3B1
AP3B2
AP3D1
AP3M1
AP3M2
AP3S1
AP3S2
AP4B1
AP4E1
AP4M1
AP4S1
AP5B1
AP5M1
AP5S1
AP5Z1
APAF1
APBA1
APBA2
APBA3
APBB1
APBB1IP
APBB2
APBB3
APC
APC2
APCDD1

APEH
APELA
APEX1
APEX2
APH1A
APH1B
API5
APIP
APLF
APLP1
APLP2
APMAP
APOA2
APOB
APOBEC3A
APOBEC3B
APOBEC3C
APOBEC3D
APOBEC3F
APOBEC3G
APOBEC4
APOBR
APOC1
APOD
APOE
APOL1
APOL2
APOL3
APOL4
APOL6
APOLD1
APOM
APOO
APOOL
APP
APPBP2
APPL1
APPL2
APRT
APTX
AQP1
AQP11
AQP3
AQP4
AQP5
AQP6
AQP7
AQP9
AQR
AR
ARAF
ARAP1
ARAP2
ARAP3
ARC
ARCN1
AREG
AREL1

ARF1
ARF3
ARF4
ARF5
ARF6
ARFGAP1
ARFGAP2
ARFGAP3
ARFGEF1
ARFGEF2
ARFGEF3
ARFIP1
ARFIP2
ARFRP1
ARG1
ARG2
ARGFXP2
ARGLU1
ARHGAP1
ARHGAP10
ARHGAP11A
ARHGAP12
ARHGAP15
ARHGAP17
ARHGAP18
ARHGAP19
ARHGAP19-SLIT1
ARHGAP20
ARHGAP21
ARHGAP22
ARHGAP23
ARHGAP24
ARHGAP25
ARHGAP26
ARHGAP27
ARHGAP28
ARHGAP29
ARHGAP30
ARHGAP31
ARHGAP32
ARHGAP33
ARHGAP35
ARHGAP39
ARHGAP4
ARHGAP42
ARHGAP42P4
ARHGAP44
ARHGAP45
ARHGAP5
ARHGAP6
ARHGAP8
ARHGAP9
ARHGDIA
ARHGDIB
ARHGEF1
ARHGEF10
ARHGEF10L
ARHGEF11

ARHGEF12
ARHGEF16
ARHGEF17
ARHGEF18
ARHGEF19
ARHGEF2
ARHGEF26
ARHGEF28
ARHGEF3
ARHGEF33
ARHGEF34P
ARHGEF35
ARHGEF37
ARHGEF38
ARHGEF39
ARHGEF4
ARHGEF40
ARHGEF5
ARHGEF6
ARHGEF7
ARHGEF9
ARID1A
ARID1B
ARID2
ARID3A
ARID3B
ARID4A
ARID4B
ARID5A
ARID5B
ARIH1
ARIH2
ARL1
ARL10
ARL11
ARL13B
ARL14EP
ARL15
ARL16
ARL17A
ARL17B
ARL2
ARL2BP
ARL3
ARL4A
ARL4C
ARL4D
ARL5A
ARL5B
ARL6
ARL6IP1
ARL6IP4
ARL6IP5
ARL6IP6
ARL8A
ARL8B
ARMC1
ARMC10

ARMC2
ARMC3
ARMC4
ARMC5
ARMC6
ARMC7
ARMC8
ARMC9
ARMCX2
ARMCX3
ARMCX4
ARMCX5
ARMCX6
ARMCX7P
ARMH1
ARMH3
ARMH4
ARMT1
ARNT
ARNT2
ARNTL
ARNTL2
ARPC1A
ARPC1B
ARPC2
ARPC3
ARPC4
ARPC4-TTLL3
ARPC5
ARPC5L
ARPIN
ARPIN-AP3S2
ARPP19
ARRB1
ARRB2
ARRDC1
ARRDC2
ARRDC3
ARRDC4
ARSA
ARSB
ARSD
ARSG
ARSJ
ARSK
ART3
ART4
ARV1
ARVCF
ARX
AS3MT
ASAH1
ASAH2
ASAH2B
ASAP1
ASAP2
ASAP3
ASB1

ASB13
ASB14
ASB16
ASB18
ASB2
ASB3
ASB4
ASB6
ASB7
ASB8
ASB9
ASCC1
ASCC2
ASCC3
ASCL3
ASF1A
ASF1B
ASGR1
ASH1L
ASH2L
ASIC1
ASIC3
ASL
ASMTL
ASNS
ASNSD1
ASPA
ASPH
ASPHD2
ASPM
ASPRV1
ASPSCR1
ASRGL1
ASS1
ASS1P2
ASTE1
ASTL
ASTN2
ASXL1
ASXL2
ASXL3
ASZ1
ATAD1
ATAD2
ATAD2B
ATAD3A
ATAD3B
ATAD3C
ATAD5
ATAT1
ATCAY
ATE1
ATF1
ATF2
ATF3
ATF4
ATF5
ATF6

ATF6B
ATF7
ATF7-NPFF
ATF7IP
ATF7IP2
ATG10
ATG101
ATG12
ATG13
ATG14
ATG16L1
ATG16L2
ATG2A
ATG2B
ATG3
ATG4A
ATG4B
ATG4C
ATG4D
ATG5
ATG7
ATG9A
ATG9B
ATIC
ATL1
ATL2
ATL3
ATM
ATMIN
ATN1
ATOH8
ATOX1
ATP10A
ATP10B
ATP10D
ATP11A
ATP11B
ATP11C
ATP12A
ATP13A1
ATP13A2
ATP13A3
ATP13A4
ATP13A5
ATP1A1
ATP1A4
ATP1B1
ATP1B2
ATP1B3
ATP1B4
ATP23
ATP2A1
ATP2A2
ATP2A3
ATP2B1
ATP2B2
ATP2B4
ATP2C1

ATP2C2
ATP5F1A
ATP5F1AP10
ATP5F1B
ATP5F1BP1
ATP5F1C
ATP5F1D
ATP5F1E
ATP5IF1
ATP5MC1
ATP5MC2
ATP5MC3
ATP5MD
ATP5ME
ATP5MF
ATP5MG
ATP5MPL
ATP5PB
ATP5PD
ATP5PF
ATP5PO
ATP6AP1
ATP6AP1L
ATP6AP2
ATP6V0A1
ATP6V0A2
ATP6V0A4
ATP6V0B
ATP6V0C
ATP6V0D1
ATP6V0D2
ATP6V0E1
ATP6V0E2
ATP6V1A
ATP6V1B1
ATP6V1B2
ATP6V1C1
ATP6V1C2
ATP6V1D
ATP6V1E1
ATP6V1E2
ATP6V1F
ATP6V1G1
ATP6V1H
ATP7A
ATP7B
ATP8A1
ATP8A2
ATP8A2P2
ATP8B1
ATP8B2
ATP8B3
ATP8B4
ATP9A
ATP9B
ATPAF1
ATPAF2
ATPSCKMT

ATR
ATRAID
ATRIP
ATRN
ATRX
ATXN1
ATXN10
ATXN1L
ATXN2
ATXN2L
ATXN3
ATXN7
ATXN7L1
ATXN7L2
ATXN7L3
ATXN7L3B
AUH
AUNIP
AUP1
AURKA
AURKAIP1
AURKB
AUTS2
AVEN
AVIL
AVL9
AVPI1
AVPR1A
AXDND1
AXIN1
AXIN2
AXL
AZGP1
AZI2
AZIN1
AZIN2
B2M
B3GALNT1
B3GALNT2
B3GALT1
B3GALT2
B3GALT4
B3GALT5
B3GALT6
B3GAT1
B3GAT2
B3GAT3
B3GLCT
B3GNT10
B3GNT2
B3GNT3
B3GNT4
B3GNT5
B3GNT6
B3GNT7
B3GNT8
B3GNT9
B3GNTL1

B4GALNT2
B4GALNT3
B4GALT1
B4GALT2
B4GALT3
B4GALT4
B4GALT5
B4GALT6
B4GALT7
B4GAT1
B9D1
B9D2
BAALC
BABAM1
BABAM2
BACE1
BACE2
BACH1
BACH2
BAD
BAG1
BAG2
BAG3
BAG4
BAG5
BAG6
BAGE2
BAHCC1
BAHD1
BAIAP2
BAIAP2L1
BAIAP3
BAK1
BAMBI
BANF1
BANK1
BANP
BAP1
BARD1
BARX2
BASP1
BATF
BATF2
BAX
BAZ1A
BAZ1B
BAZ2A
BAZ2B
BBC3
BBIP1
BBOF1
BBOX1
BBS1
BBS12
BBS2
BBS4
BBS5
BBS7

BBS9
BBX
BCAM
BCAP29
BCAP31
BCAR1
BCAR3
BCAS1
BCAS2
BCAS2P2
BCAS3
BCAS4
BCAT1
BCAT2
BCCIP
BCDIN3D
BCKDHA
BCKDHB
BCKDK
BCL10
BCL11A
BCL11B
BCL2
BCL2A1
BCL2L1
BCL2L11
BCL2L12
BCL2L13
BCL2L14
BCL2L15
BCL2L2
BCL2L2-PABPN1
BCL3
BCL6
BCL7A
BCL7B
BCL7C
BCL9
BCL9L
BCLAF1
BCLAF3
BCO1
BCO2
BCOR
BCORL1
BCORP1
BCR
BCRP2
BCS1L
BDH1
BDH2
BDKRB1
BDKRB2
BDNF
BDP1
BEAN1
BECN1
BEGAIN

BEND2
BEND3
BEND3P1
BEND4
BEND5
BEND6
BEND7
BEST1
BEST3
BEST4
BET1
BET1L
BEX2
BEX3
BEX4
BFAR
BGN
BHLHB9
BHLHE40
BHLHE41
BHMT
BHMT2
BICC1
BICD1
BICD2
BICDL1
BICDL2
BICRA
BICRAL
BID
BIK
BIN1
BIN2
BIN3
BIRC2
BIRC3
BIRC5
BIRC6
BIVM
BLACAT1
BLCAP
BLK
BLM
BLMH
BLNK
BLOC1S1
BLOC1S2
BLOC1S3
BLOC1S4
BLOC1S5
BLOC1S6
BLVRA
BLVRB
BLZF1
BMERB1
BMF
BMI1
BMP1

BMP10
BMP2K
BMP3
BMP4
BMP6
BMP7
BMP8A
BMP8B
BMPR1A
BMPR1B
BMPR2
BMS1
BMS1P1
BMS1P17
BMS1P2
BMS1P4
BMS1P7
BMT2
BMX
BNC1
BNC2
BNIP1
BNIP2
BNIP3
BNIP3L
BNIP3P28
BNIPL
BOC
BOD1
BOD1L1
BOK
BOLA1
BOLA2
BOLA2-SMG1P6
BOLA2B
BOLA3
BOLL
BOP1
BORCS5
BORCS6
BORCS7
BORCS8
BORCS8-MEF2B
BPGM
BPHL
BPI
BPIFA1
BPIFA2
BPIFB1
BPIFB2
BPNT1
BPTF
BPTFP1
BRAF
BRAP
BRAT1
BRCA1
BRCA2

BRCC3
BRD1
BRD2
BRD3
BRD3OS
BRD4
BRD7
BRD7P4
BRD8
BRD9
BRF1
BRF2
BRI3
BRI3BP
BRIP1
BRIX1
BRK1
BRMS1
BRMS1L
BROX
BRPF1
BRPF3
BRWD1
BRWD3
BSCL2
BSDC1
BSG
BSN
BSPRY
BST1
BST2
BTAF1
BTBD1
BTBD10
BTBD11
BTBD18
BTBD19
BTBD2
BTBD3
BTBD6
BTBD7
BTBD7P1
BTBD8
BTBD9
BTC
BTD
BTF3
BTF3L4
BTG1
BTG2
BTG3
BTG4
BTK
BTLA
BTN2A1
BTN2A2
BTN2A3P
BTN3A1

BTN3A2
BTN3A3
BTNL3
BTNL8
BTNL9
BTRC
BUB1
BUB1B
BUB3
BUD13
BUD23
BUD31
BVES
BX004987.1
BX005195.1
BX284613.1
BX322639.1
BYSL
BZW1
BZW1P2
BZW2
C10orf105
C10orf53
C10orf55
C10orf67
C10orf82
C10orf88
C10orf95
C11orf1
C11orf16
C11orf21
C11orf24
C11orf45
C11orf49
C11orf52
C11orf54
C11orf58
C11orf65
C11orf68
C11orf71
C11orf80
C11orf88
C11orf95
C11orf97
C11orf98
C12orf10
C12orf29
C12orf4
C12orf40
C12orf43
C12orf45
C12orf49
C12orf50
C12orf57
C12orf60
C12orf65
C12orf66
C12orf73

C12orf75
C12orf76
C13orf42
C13orf46
C14orf119
C14orf132
C14orf28
C14orf39
C14orf93
C15orf39
C15orf40
C15orf41
C15orf48
C15orf61
C15orf62
C15orf65
C16orf46
C16orf54
C16orf58
C16orf70
C16orf71
C16orf72
C16orf74
C16orf87
C16orf89
C16orf91
C16orf95
C17orf100
C17orf107
C17orf113
C17orf49
C17orf58
C17orf67
C17orf75
C17orf80
C17orf97
C18orf21
C18orf25
C18orf32
C18orf54
C19orf12
C19orf25
C19orf33
C19orf38
C19orf44
C19orf47
C19orf48
C19orf53
C19orf54
C1D
C1GALT1
C1GALT1C1
C1GALT1C1L
C1orf109
C1orf112
C1orf115
C1orf116
C1orf122

C1orf127
C1orf131
C1orf141
C1orf146
C1orf158
C1orf159
C1orf162
C1orf167
C1orf174
C1orf189
C1orf194
C1orf198
C1orf21
C1orf210
C1orf216
C1orf226
C1orf35
C1orf43
C1orf50
C1orf52
C1orf54
C1orf56
C1orf61
C1orf74
C1orf87
C1QA
C1QB
C1QBP
C1QC
C1QL3
C1QTNF1
C1QTNF3
C1QTNF5
C1QTNF6
C1QTNF7
C1R
C1RL
C1S
C2
C20orf194
C20orf27
C20orf85
C20orf96
C21orf58
C21orf62
C21orf91
C22orf15
C22orf23
C22orf39
C22orf46
C2CD2
C2CD2L
C2CD3
C2CD4A
C2CD4B
C2CD5
C2orf15
C2orf16

C2orf27A
C2orf42
C2orf49
C2orf50
C2orf66
C2orf68
C2orf69
C2orf73
C2orf74
C2orf76
C2orf83
C2orf88
C2orf91
C2orf92
C3
C3AR1
C3orf14
C3orf18
C3orf20
C3orf33
C3orf38
C3orf52
C3orf62
C3orf67
C3orf70
C3orf86
C4A
C4B
C4B_2
C4BPA
C4BPB
C4orf17
C4orf19
C4orf3
C4orf33
C4orf36
C4orf46
C4orf47
C5
C5AR1
C5AR2
C5orf15
C5orf22
C5orf24
C5orf34
C5orf49
C5orf51
C5orf58
C5orf63
C6
C6orf118
C6orf120
C6orf132
C6orf136
C6orf141
C6orf163
C6orf223
C6orf47

C6orf62
C6orf89
C7
C7orf25
C7orf26
C7orf31
C7orf50
C7orf57
C8orf33
C8orf34
C8orf37
C8orf58
C8orf76
C8orf82
C9orf116
C9orf131
C9orf135
C9orf152
C9orf16
C9orf24
C9orf40
C9orf43
C9orf64
C9orf72
C9orf78
C9orf85
CA1
CA12
CA13
CA14
CA2
CA4
CA5A
CA5B
CA5BP1
CA6
CA8
CAAP1
CAB39
CAB39L
CABCOCO1
CABIN1
CABLES1
CABLES2
CABP1
CABP4
CABYR
CACFD1
CACHD1
CACNA1A
CACNA1B
CACNA1D
CACNA1E
CACNA1F
CACNA2D1
CACNA2D2
CACNA2D3
CACNB1

CACNB2
CACNB3
CACNB4
CACNG4
CACNG6
CACNG8
CACTIN
CACUL1
CACYBP
CAD
CADM1
CADM4
CADPS
CADPS2
CALB1
CALCOCO1
CALCOCO2
CALCRL
CALD1
CALHM2
CALHM5
CALHM6
CALM1
CALM2
CALM3
CALML3
CALML4
CALN1
CALR
CALU
CAMK1
CAMK1D
CAMK1G
CAMK2B
CAMK2D
CAMK2G
CAMK2N1
CAMK4
CAMKK1
CAMKK2
CAMKMT
CAMLG
CAMSAP1
CAMSAP2
CAMSAP3
CAMTA1
CAMTA2
CAND1
CAND2
CANT1
CANX
CAP1
CAP2
CAPG
CAPN1
CAPN10
CAPN12
CAPN13

CAPN14
CAPN15
CAPN2
CAPN3
CAPN5
CAPN7
CAPN8
CAPN9
CAPNS1
CAPRIN1
CAPRIN2
CAPS
CAPS2
CAPSL
CAPZA1
CAPZA2
CAPZB
CARD10
CARD11
CARD14
CARD16
CARD19
CARD6
CARD8
CARD9
CARF
CARHSP1
CARM1
CARMIL1
CARMIL2
CARNMT1
CARNS1
CARS1
CARS2
CASC1
CASC3
CASC4
CASD1
CASK
CASKIN2
CASP1
CASP10
CASP14
CASP16P
CASP17P
CASP2
CASP3
CASP4
CASP5
CASP6
CASP7
CASP8
CASP8AP2
CASP9
CASS4
CAST
CASTOR1
CASTOR2

CASTOR3
CASZ1
CAT
CATIP
CATSPER2
CATSPER2P1
CATSPERB
CATSPERD
CATSPERE
CATSPERG
CAV1
CAV2
CAVIN1
CAVIN2
CBFA2T2
CBFA2T3
CBFB
CBL
CBLB
CBLC
CBLL1
CBLN3
CBR1
CBR3
CBR4
CBS
CBWD1
CBWD2
CBWD3
CBWD5
CBWD6
CBX1
CBX3
CBX4
CBX5
CBX6
CBX7
CBY1
CC2D1A
CC2D1B
CC2D2A
CC2D2B
CCAR1
CCAR2
CCBE1
CCDC102A
CCDC102B
CCDC103
CCDC106
CCDC107
CCDC110
CCDC112
CCDC113
CCDC114
CCDC115
CCDC117
CCDC12
CCDC120

CCDC121
CCDC122
CCDC124
CCDC125
CCDC126
CCDC127
CCDC13
CCDC130
CCDC134
CCDC136
CCDC137
CCDC138
CCDC14
CCDC141
CCDC142
CCDC144A
CCDC144B
CCDC144CP
CCDC146
CCDC148
CCDC149
CCDC15
CCDC150
CCDC151
CCDC152
CCDC153
CCDC157
CCDC158
CCDC159
CCDC160
CCDC162P
CCDC163
CCDC168
CCDC169
CCDC17
CCDC170
CCDC171
CCDC173
CCDC174
CCDC175
CCDC18
CCDC180
CCDC181
CCDC186
CCDC187
CCDC189
CCDC190
CCDC191
CCDC198
CCDC200
CCDC22
CCDC24
CCDC25
CCDC27
CCDC28A
CCDC28A-AS1
CCDC28B
CCDC30

CCDC32
CCDC33
CCDC34
CCDC36
CCDC38
CCDC39
CCDC40
CCDC43
CCDC47
CCDC50
CCDC51
CCDC57
CCDC58
CCDC59
CCDC6
CCDC60
CCDC61
CCDC62
CCDC65
CCDC66
CCDC68
CCDC69
CCDC7
CCDC71
CCDC71L
CCDC73
CCDC74A
CCDC74B
CCDC77
CCDC78
CCDC8
CCDC80
CCDC81
CCDC82
CCDC84
CCDC85B
CCDC85C
CCDC86
CCDC88A
CCDC88B
CCDC88C
CCDC89
CCDC9
CCDC90B
CCDC91
CCDC92
CCDC93
CCDC96
CCDC97
CCDC9B
CCHCR1
CCL14
CCL15
CCL15-CCL14
CCL16
CCL2
CCL20
CCL22

CCL28
CCL3
CCL3L1
CCL3L3
CCL4
CCL4L2
CCL5
CCL8
CCM2
CCN1
CCN2
CCN3
CCN4
CCN5
CCNA1
CCNA2
CCNB1
CCNB1IP1
CCNB2
CCNB3
CCNB3P1
CCNC
CCND1
CCND2
CCND3
CCNDBP1
CCNE1
CCNE2
CCNF
CCNG1
CCNG1P1
CCNG2
CCNH
CCNI
CCNI2
CCNJ
CCNJL
CCNK
CCNL1
CCNL2
CCNO
CCNQ
CCNT1
CCNT2
CCNY
CCNYL1
CCNYL2
CCP110
CCPG1
CCR1
CCR2
CCR3
CCR4
CCR5
CCR6
CCR7
CCRL2
CCS

CCSAP
CCSER1
CCSER2
CCT2
CCT3
CCT4
CCT5
CCT5P1
CCT6A
CCT6B
CCT6P1
CCT6P3
CCT7
CCT7P1
CCT8
CCT8P1
CCZ1
CCZ1B
CD101
CD109
CD14
CD151
CD160
CD163
CD163L1
CD164
CD164L2
CD177
CD180
CD19
CD1C
CD1E
CD2
CD200
CD200R1
CD200R1L
CD207
CD209
CD22
CD226
CD24
CD244
CD247
CD27
CD274
CD276
CD28
CD2AP
CD2BP2
CD300A
CD300E
CD300LB
CD300LF
CD300LG
CD302
CD320
CD33
CD34

CD36
CD37
CD38
CD3D
CD3E
CD3EAP
CD3G
CD4
CD40
CD40LG
CD44
CD46
CD46P1
CD47
CD48
CD5
CD52
CD53
CD55
CD58
CD59
CD6
CD63
CD68
CD69
CD7
CD72
CD74
CD79A
CD79B
CD80
CD81
CD82
CD83
CD84
CD86
CD8A
CD8B
CD9
CD93
CD96
CD99
CD99L2
CD99P1
CDA
CDADC1
CDAN1
CDC123
CDC14A
CDC14B
CDC16
CDC20
CDC20B
CDC23
CDC25A
CDC25B
CDC26
CDC27

CDC27P1
CDC34
CDC37
CDC37L1
CDC40
CDC42
CDC42BPA
CDC42BPB
CDC42BPG
CDC42EP1
CDC42EP2
CDC42EP3
CDC42EP4
CDC42SE1
CDC42SE2
CDC5L
CDC6
CDC7
CDC73
CDCA2
CDCA3
CDCA4
CDCA7
CDCA7L
CDCA8
CDCP1
CDH1
CDH11
CDH12
CDH13
CDH15
CDH17
CDH18
CDH19
CDH2
CDH23
CDH24
CDH26
CDH3
CDH4
CDH6
CDH7
CDH8
CDHR3
CDHR4
CDIP1
CDIPT
CDK1
CDK10
CDK11A
CDK11B
CDK12
CDK13
CDK14
CDK15
CDK16
CDK17
CDK18

CDK19
CDK2
CDK20
CDK2AP1
CDK2AP2
CDK4
CDK5
CDK5R1
CDK5RAP1
CDK5RAP2
CDK5RAP3
CDK6
CDK7
CDK8
CDK9
CDKAL1
CDKL1
CDKL2
CDKL3
CDKL5
CDKN1A
CDKN1B
CDKN1C
CDKN2A
CDKN2AIP
CDKN2AIPNL
CDKN2B
CDKN2C
CDKN2D
CDNF
CDON
CDR2
CDR2L
CDRT1
CDRT4
CDS1
CDS2
CDT1
CDV3
CDYL
CDYL2
CEACAM1
CEACAM19
CEACAM22P
CEACAM3
CEACAM4
CEACAM5
CEACAM6
CEACAM7
CEACAM8
CEBPA
CEBPB
CEBPD
CEBPG
CEBPZ
CEBPZOS
CECR2
CECR7

CEL
CELF1
CELF2
CELF5
CELF6
CELP
CELSR1
CELSR2
CELSR3
CEMIP
CEMIP2
CENPB
CENPBD1P1
CENPC
CENPE
CENPF
CENPH
CENPI
CENPJ
CENPK
CENPL
CENPM
CENPN
CENPNP1
CENPO
CENPP
CENPQ
CENPS
CENPS-CORT
CENPT
CENPU
CENPUP2
CENPV
CENPX
CEP104
CEP112
CEP120
CEP126
CEP128
CEP131
CEP135
CEP152
CEP162
CEP164
CEP164P1
CEP170
CEP170B
CEP170P1
CEP19
CEP192
CEP250
CEP290
CEP295
CEP295NL
CEP350
CEP41
CEP44
CEP55

CEP57
CEP57L1
CEP63
CEP68
CEP70
CEP72
CEP76
CEP78
CEP83
CEP85
CEP85L
CEP89
CEP95
CEP97
CEPT1
CERCAM
CERK
CERKL
CERS2
CERS3
CERS4
CERS5
CERS6
CERT1
CES1
CES1P1
CES2
CES3
CES4A
CETN2
CETN3
CFAP100
CFAP126
CFAP157
CFAP161
CFAP20
CFAP206
CFAP221
CFAP298
CFAP298-TCP10L
CFAP299
CFAP300
CFAP36
CFAP410
CFAP43
CFAP44
CFAP45
CFAP46
CFAP47
CFAP52
CFAP53
CFAP54
CFAP57
CFAP58
CFAP61
CFAP65
CFAP69
CFAP70

CFAP73
CFAP74
CFAP77
CFAP97
CFAP97D1
CFAP97D2
CFAP99
CFB
CFD
CFDP1
CFH
CFI
CFL1
CFL1P1
CFL1P5
CFL2
CFLAR
CFP
CFTR
CGAS
CGGBP1
CGN
CGNL1
CGRRF1
CH25H
CHAC1
CHAD
CHAF1A
CHAF1B
CHAMP1
CHCHD1
CHCHD10
CHCHD2
CHCHD3
CHCHD4
CHCHD5
CHCHD6
CHCHD7
CHD1
CHD1L
CHD2
CHD3
CHD4
CHD5
CHD6
CHD7
CHD8
CHD9
CHDH
CHEK1
CHEK2
CHERP
CHFR
CHGB
CHI3L1
CHI3L2
CHIAP2
CHIC1

CHIC2
CHID1
CHIT1
CHKA
CHKB
CHKB-CPT1B
CHL1
CHM
CHML
CHMP1A
CHMP1B
CHMP1B2P
CHMP2A
CHMP2B
CHMP3
CHMP4A
CHMP4B
CHMP4C
CHMP5
CHMP6
CHMP7
CHN1
CHN2
CHODL
CHORDC1
CHORDC1P3
CHORDC1P4
CHP1
CHP2
CHPF
CHPF2
CHPT1
CHRAC1
CHRDL1
CHRM3
CHRM5
CHRNA10
CHRNA2
CHRNA4
CHRNA5
CHRNA7
CHRNA9
CHRNB1
CHRNB2
CHRNB3
CHRNB4
CHST1
CHST10
CHST11
CHST12
CHST14
CHST15
CHST2
CHST3
CHST4
CHST5
CHST6
CHST7

CHST9
CHSY1
CHTF18
CHTF8
CHTOP
CHUK
CHURC1
CHURC1-FNTB
CIAO1
CIAO2A
CIAO2B
CIAO3
CIAPIN1
CIART
CIB1
CIB2
CIC
CICP14
CICP27
CIDEB
CIDECP1
CIITA
CILK1
CINP
CIP2A
CIPC
CIR1
CIRBP
CISD1
CISD2
CISD3
CISH
CIT
CITED2
CITED4
CIZ1
CKAP2
CKAP2L
CKAP4
CKAP5
CKB
CKLF
CKLF-CMTM1
CKMT1A
CKMT1B
CKS1B
CKS2
CLASP1
CLASP2
CLASRP
CLBA1
CLCA2
CLCA3P
CLCA4
CLCC1
CLCF1
CLCN2
CLCN3

CLCN4
CLCN5
CLCN6
CLCN7
CLCNKA
CLCNKB
CLDN1
CLDN10
CLDN11
CLDN12
CLDN15
CLDN16
CLDN18
CLDN22
CLDN23
CLDN3
CLDN4
CLDN7
CLDN8
CLDN9
CLDND1
CLEC12A
CLEC12B
CLEC16A
CLEC17A
CLEC18A
CLEC1A
CLEC1B
CLEC2B
CLEC2D
CLEC4A
CLEC4D
CLEC4E
CLEC5A
CLEC7A
CLEC9A
CLECL1
CLGN
CLHC1
CLIC1
CLIC2
CLIC3
CLIC4
CLIC5
CLIC6
CLINT1
CLIP1
CLIP2
CLIP4
CLK1
CLK2
CLK3
CLK4
CLMN
CLMP
CLN3
CLN5
CLN6

CLN8
CLNK
CLNS1A
CLOCK
CLP1
CLPB
CLPP
CLPTM1
CLPTM1L
CLPX
CLSPN
CLSTN1
CLSTN2
CLSTN3
CLTA
CLTB
CLTC
CLTCL1
CLU
CLUAP1
CLUH
CLUHP3
CLUL1
CLVS1
CLYBL
CMAHP
CMAS
CMBL
CMC1
CMC2
CMC4
CMIP
CMKLR1
CMPK1
CMPK2
CMSS1
CMTM2
CMTM3
CMTM4
CMTM6
CMTM7
CMTM8
CMTR1
CMTR2
CMYA5
CNBD2
CNBP
CNDP1
CNDP2
CNEP1R1
CNFN
CNGA1
CNGA3
CNGA4
CNGB1
CNIH1
CNIH3
CNIH4

CNKSR1
CNKSR3
CNN1
CNN2
CNN3
CNNM1
CNNM2
CNNM3
CNNM4
CNOT1
CNOT10
CNOT11
CNOT2
CNOT3
CNOT4
CNOT6
CNOT6L
CNOT7
CNOT8
CNOT9
CNP
CNPPD1
CNPY2
CNPY3
CNPY4
CNR1
CNR2
CNST
CNTD1
CNTF
CNTLN
CNTN2
CNTN3
CNTN4
CNTN5
CNTNAP1
CNTNAP2
CNTNAP3
CNTNAP3B
CNTNAP3C
CNTNAP5
CNTRL
CNTROB
COA1
COA3
COA4
COA5
COA6
COA7
COA8
COASY
COBL
COBLL1
COCH
COG1
COG2
COG3
COG4

COG5
COG6
COG7
COG8
COIL
COL10A1
COL12A1
COL14A1
COL16A1
COL17A1
COL18A1
COL19A1
COL1A1
COL1A2
COL21A1
COL24A1
COL27A1
COL28A1
COL3A1
COL4A2
COL4A3
COL4A4
COL4A5
COL4A6
COL5A1
COL6A1
COL6A3
COL6A6
COL7A1
COL8A1
COL8A2
COL9A1
COL9A2
COL9A3
COLCA2
COLEC10
COLEC12
COLGALT1
COLGALT2
COLQ
COMMD1
COMMD10
COMMD2
COMMD3
COMMD3-BMI1
COMMD4
COMMD5
COMMD6
COMMD7
COMMD8
COMMD9
COMP
COMT
COMTD1
COP1
COPA
COPB1
COPB2

COPE
COPG1
COPG2
COPRS
COPS2
COPS3
COPS4
COPS5
COPS6
COPS7A
COPS7B
COPS8
COPS9
COPZ1
COPZ2
COQ10A
COQ10B
COQ2
COQ3
COQ4
COQ5
COQ6
COQ7
COQ8A
COQ8B
COQ9
CORIN
CORO1A
CORO1B
CORO1C
CORO2A
CORO2B
CORO6
CORO7
CORO7-PAM16
COTL1
COX10
COX11
COX14
COX15
COX16
COX17
COX18
COX19
COX20
COX4I1
COX5A
COX5B
COX6A1
COX6B1
COX6B2
COX6C
COX7A2
COX7A2L
COX7B
COX7C
COX8A
CP

CPA3
CPA4
CPAMD8
CPD
CPEB1
CPEB2
CPEB3
CPEB4
CPED1
CPLANE1
CPLANE2
CPM
CPN2
CPNE1
CPNE2
CPNE3
CPNE4
CPNE5
CPNE8
CPOX
CPPED1
CPQ
CPS1
CPSF1
CPSF2
CPSF3
CPSF4
CPSF6
CPSF7
CPT1A
CPT1B
CPT1C
CPT2
CPTP
CPVL
CPXM2
CR1
CR1L
CR2
CR382285.1
CR383656.13
CR759762.1
CR788268.1
CRABP2
CRACD
CRACR2A
CRACR2B
CRADD
CRAMP1
CRAT
CRB1
CRB2
CRB3
CRBN
CRCP
CREB1
CREB3
CREB3L1

CREB3L2
CREB3L4
CREB5
CREBBP
CREBL2
CREBRF
CREBZF
CREG1
CREG2
CRELD1
CRELD2
CREM
CRIM1
CRIP1
CRIP2
CRIPT
CRISP3
CRISPLD1
CRISPLD2
CRK
CRKL
CRLF1
CRLF3
CRLS1
CRMP1
CRNKL1
CRNN
CROCC
CROCC2
CROCCP2
CROCCP3
CROT
CRPPA
CRTAM
CRTAP
CRTC1
CRTC2
CRTC3
CRX
CRY1
CRY2
CRYBB2P1
CRYBG1
CRYBG2
CRYBG3
CRYL1
CRYM
CRYM-AS1
CRYZ
CRYZL1
CRYZL2P
CS
CSAD
CSDE1
CSE1L
CSF1
CSF1R
CSF2RA

CSF2RB
CSF3
CSF3R
CSGALNACT1
CSGALNACT2
CSK
CSKMT
CSMD1
CSMD2
CSMD3
CSNK1A1
CSNK1D
CSNK1E
CSNK1G1
CSNK1G2
CSNK1G3
CSNK2A1
CSNK2A2
CSNK2B
CSPG4
CSPG4P10
CSPG4P11
CSPG4P12
CSPP1
CSRNP1
CSRNP2
CSRNP3
CSRP1
CSRP2
CST1
CST3
CST7
CSTA
CSTB
CSTF1
CSTF2
CSTF2T
CSTF3
CT75
CTAGE3P
CTAGE4
CTBP1
CTBP2
CTBS
CTC1
CTCF
CTCFL
CTDNEP1
CTDP1
CTDSP1
CTDSP2
CTDSPL
CTDSPL2
CTF1
CTH
CTIF
CTLA4
CTNNA1

CTNNAL1
CTNNB1
CTNNBIP1
CTNNBL1
CTNND1
CTNS
CTPS1
CTPS2
CTR9
CTRC
CTRL
CTSA
CTSB
CTSC
CTSD
CTSE
CTSF
CTSH
CTSK
CTSL
CTSO
CTSS
CTSV
CTSW
CTSZ
CTTN
CTTNBP2
CTTNBP2NL
CTU2
CTXN1
CTXND1
CUBN
CUEDC1
CUEDC2
CUL1
CUL2
CUL3
CUL4A
CUL4B
CUL5
CUL7
CUL9
CUTA
CUTALP
CUTC
CUX1
CWC15
CWC22
CWC25
CWC27
CWF19L1
CWF19L2
CWH43
CX3CL1
CX3CR1
CXADR
CXCL1
CXCL10

CXCL11
CXCL12
CXCL13
CXCL16
CXCL17
CXCL2
CXCL3
CXCL5
CXCL6
CXCL8
CXCL9
CXCR1
CXCR2
CXCR2P1
CXCR3
CXCR4
CXCR5
CXCR6
CXorf21
CXorf38
CXorf56
CXXC1
CXXC4
CXXC5
CYB561
CYB561A3
CYB561D1
CYB561D2
CYB5A
CYB5AP2
CYB5B
CYB5D1
CYB5D2
CYB5R1
CYB5R2
CYB5R3
CYB5R4
CYB5RL
CYBA
CYBB
CYBC1
CYBRD1
CYC1
CYCS
CYFIP1
CYFIP2
CYGB
CYHR1
CYLD
CYP11A1
CYP19A1
CYP1A2
CYP1B1
CYP20A1
CYP21A1P
CYP24A1
CYP26A1
CYP27A1

CYP27C1
CYP2A13
CYP2A6
CYP2A7
CYP2B6
CYP2B7P
CYP2C18
CYP2C19
CYP2C8
CYP2C9
CYP2E1
CYP2F1
CYP2G1P
CYP2G2P
CYP2J2
CYP2R1
CYP2S1
CYP2U1
CYP2W1
CYP39A1
CYP3A43
CYP3A5
CYP46A1
CYP4A11
CYP4A22
CYP4B1
CYP4F11
CYP4F12
CYP4F3
CYP4F35P
CYP4F8
CYP4V2
CYP4X1
CYP4Z1
CYP4Z2P
CYP51A1
CYP7B1
CYP8B1
CYREN
CYRIA
CYRIB
CYSLTR1
CYSLTR2
CYSTM1
CYTH1
CYTH2
CYTH3
CYTH4
CYTIP
CYYR1
CZIB
D2HGDH
DAAM1
DAAM2
DAB1
DAB2
DAB2IP
DACH1

DACT2
DACT3
DAD1
DAG1
DAGLA
DAGLB
DALRD3
DAND5
DAP
DAP3
DAPK1
DAPK2
DAPK3
DAPL1
DAPP1
DARS1
DARS2
DAW1
DAXX
DAZAP1
DAZAP2
DBF4
DBF4B
DBI
DBN1
DBNDD1
DBNDD2
DBNL
DBP
DBR1
DBT
DCAF1
DCAF10
DCAF11
DCAF12
DCAF13
DCAF15
DCAF16
DCAF17
DCAF4
DCAF4L1
DCAF5
DCAF6
DCAF7
DCAF8
DCAKD
DCBLD1
DCBLD2
DCC
DCDC1
DCDC2
DCDC2B
DCHS2
DCK
DCLK1
DCLK3
DCLRE1A
DCLRE1B

DCLRE1C
DCN
DCP1A
DCP1B
DCP2
DCPS
DCST1
DCST2
DCTD
DCTN1
DCTN2
DCTN3
DCTN4
DCTN5
DCTN6
DCTPP1
DCUN1D1
DCUN1D2
DCUN1D3
DCUN1D4
DCUN1D5
DCX
DCXR
DDA1
DDAH1
DDAH2
DDB1
DDB2
DDHD1
DDHD2
DDI2
DDIAS
DDIT3
DDIT4
DDIT4L
DDO
DDOST
DDR1
DDR2
DDRGK1
DDT
DDTL
DDX1
DDX10
DDX10P1
DDX11
DDX17
DDX18
DDX18P1
DDX18P5
DDX19A
DDX19B
DDX20
DDX21
DDX23
DDX24
DDX25
DDX27

DDX28
DDX31
DDX39A
DDX39B
DDX3P1
DDX3X
DDX3Y
DDX41
DDX42
DDX43
DDX46
DDX47
DDX49
DDX5
DDX50
DDX50P1
DDX51
DDX52
DDX54
DDX55
DDX56
DDX58
DDX59
DDX6
DDX60
DDX60L
DEAF1
DECR1
DECR2
DEDD
DEDD2
DEF6
DEF8
DEGS1
DEGS2
DEK
DELE1
DELEC1
DENND10
DENND10P1
DENND11
DENND1A
DENND1B
DENND1C
DENND2A
DENND2B
DENND2C
DENND2D
DENND3
DENND4A
DENND4B
DENND4C
DENND5A
DENND5B
DENND6A
DENND6B
DENR
DEPDC1

DEPDC1B
DEPDC4
DEPDC5
DEPP1
DEPTOR
DERA
DERL1
DERL2
DERL3
DESI1
DESI2
DET1
DEUP1
DEXI
DFFA
DFFB
DGAT1
DGAT2
DGCR2
DGCR5
DGCR6
DGCR6L
DGCR8
DGKA
DGKB
DGKD
DGKE
DGKG
DGKH
DGKI
DGKQ
DGKZ
DGKZP1
DGLUCY
DGUOK
DHCR24
DHCR7
DHDDS
DHFR
DHFR2
DHODH
DHPS
DHRS1
DHRS11
DHRS12
DHRS13
DHRS2
DHRS3
DHRS4
DHRS4L2
DHRS7
DHRS7B
DHRS9
DHRSX
DHTKD1
DHX15
DHX16
DHX29

DHX30
DHX32
DHX33
DHX34
DHX35
DHX36
DHX37
DHX38
DHX40
DHX57
DHX58
DHX8
DHX9
DIABLO
DIAPH1
DIAPH2
DIAPH3
DICER1
DIDO1
DIMT1
DIO1
DIO2
DIP2A
DIP2B
DIP2C
DIPK1A
DIPK2A
DIPK2B
DIRC1
DIS3
DIS3L
DIS3L2
DISC1
DISP1
DISP2
DIXDC1
DKC1
DKK1
DKK3
DLAT
DLC1
DLD
DLEC1
DLEU7
DLG1
DLG2
DLG3
DLG5
DLGAP1
DLGAP2
DLGAP3
DLGAP4
DLGAP5
DLK2
DLL1
DLST
DMAC1
DMAC2

DMAC2L
DMAP1
DMBT1
DMC1
DMD
DMGDH
DMKN
DMPK
DMRT2
DMRT3
DMRTA1
DMRTA2
DMTF1
DMTN
DMWD
DMXL1
DMXL2
DNA2
DNAAF1
DNAAF2
DNAAF3
DNAAF4
DNAAF5
DNAH1
DNAH10
DNAH11
DNAH12
DNAH14
DNAH17
DNAH2
DNAH3
DNAH5
DNAH6
DNAH7
DNAH8
DNAH9
DNAI1
DNAI2
DNAJA1
DNAJA2
DNAJA3
DNAJA4
DNAJB1
DNAJB11
DNAJB12
DNAJB13
DNAJB14
DNAJB2
DNAJB4
DNAJB5
DNAJB6
DNAJB7
DNAJB9
DNAJC1
DNAJC10
DNAJC11
DNAJC13
DNAJC14

DNAJC15
DNAJC16
DNAJC17
DNAJC18
DNAJC19
DNAJC2
DNAJC21
DNAJC22
DNAJC24
DNAJC25
DNAJC25-GNG10
DNAJC27
DNAJC28
DNAJC3
DNAJC30
DNAJC4
DNAJC5
DNAJC5B
DNAJC6
DNAJC7
DNAJC8
DNAJC9
DNAL1
DNAL4
DNALI1
DNASE1
DNASE1L1
DNASE1L3
DNASE2
DND1
DNER
DNHD1
DNM1
DNM1L
DNM1P46
DNM1P47
DNM1P51
DNM2
DNM3
DNMBP
DNMT1
DNMT3A
DNMT3B
DNPEP
DNPH1
DNTTIP1
DNTTIP2
DOC2A
DOC2B
DOCK1
DOCK10
DOCK11
DOCK2
DOCK3
DOCK4
DOCK5
DOCK6
DOCK7

DOCK8
DOCK8-AS1
DOCK9
DOHH
DOK1
DOK2
DOK3
DOK4
DOK6
DOLK
DOLPP1
DONSON
DOP1A
DOP1B
DOT1L
DPAGT1
DPCD
DPEP2
DPF1
DPF2
DPF3
DPH1
DPH2
DPH3
DPH5
DPH6
DPH7
DPM1
DPM2
DPM3
DPP10
DPP3
DPP4
DPP7
DPP8
DPP9
DPPA4
DPY19L1
DPY19L1P1
DPY19L1P2
DPY19L2
DPY19L2P1
DPY19L2P2
DPY19L2P3
DPY19L2P4
DPY19L3
DPY19L4
DPY30
DPYD
DPYS
DPYSL2
DPYSL3
DPYSL5
DR1
DRAM1
DRAM2
DRAP1
DRAXIN

DRC1
DRC3
DRC7
DRD2
DRG1
DRG1P1
DRG2
DROSHA
DSC2
DSC3
DSCAML1
DSE
DSEL
DSG2
DSG3
DSN1
DSP
DST
DSTN
DSTNP2
DSTYK
DTD1
DTD2
DTHD1
DTL
DTNA
DTNB
DTNBP1
DTWD1
DTWD2
DTX1
DTX2
DTX2P1
DTX3
DTX3L
DTX4
DTYMK
DUOX1
DUOX2
DUOXA1
DUOXA2
DUS1L
DUS2
DUS3L
DUS4L
DUSP1
DUSP10
DUSP11
DUSP12
DUSP14
DUSP16
DUSP18
DUSP19
DUSP2
DUSP22
DUSP23
DUSP28
DUSP3

DUSP4
DUSP5
DUSP6
DUSP7
DUSP8
DUT
DUX4
DUX4L19
DUX4L26
DUXAP9
DVL1
DVL2
DVL3
DXO
DYDC1
DYDC2
DYM
DYNAP
DYNC1H1
DYNC1I1
DYNC1I2
DYNC1LI1
DYNC1LI2
DYNC2H1
DYNC2LI1
DYNLL1
DYNLL2
DYNLRB1
DYNLRB2
DYNLT1
DYNLT3
DYRK1A
DYRK1B
DYRK2
DYRK3
DYRK4
DYSF
DZANK1
DZIP1
DZIP1L
DZIP3
E2F2
E2F3
E2F3P2
E2F4
E2F5
E2F6
E2F7
E2F8
E4F1
EAF1
EAF2
EAPP
EARS2
EBAG9
EBF1
EBF2
EBF4

EBLN2
EBNA1BP2
EBP
EBPL
ECD
ECE1
ECE2
ECH1
ECHDC1
ECHDC2
ECHDC3
ECHS1
ECI1
ECI2
ECM1
ECM2
ECPAS
ECRG4
ECSIT
ECT2
ECT2L
EDA
EDAR
EDARADD
EDC3
EDC4
EDEM1
EDEM2
EDEM3
EDF1
EDIL3
EDN1
EDRF1
EEA1
EED
EEF1A1
EEF1A1P3
EEF1A1P33
EEF1A1P38
EEF1A1P4
EEF1A1P5
EEF1A1P6
EEF1AKMT1
EEF1AKMT2
EEF1AKMT3
EEF1AKNMT
EEF1B2
EEF1D
EEF1DP3
EEF1E1
EEF1G
EEF2
EEF2K
EEF2KMT
EEFSEC
EEPD1
EFCAB1
EFCAB10

EFCAB11
EFCAB12
EFCAB13
EFCAB14
EFCAB2
EFCAB3
EFCAB5
EFCAB6
EFCAB7
EFCC1
EFEMP1
EFEMP2
EFHB
EFHC1
EFHC2
EFHD1
EFHD2
EFL1
EFL1P1
EFNA1
EFNA4
EFNA5
EFNB1
EFNB2
EFNB3
EFR3A
EFR3B
EFS
EFTUD2
EGF
EGFL6
EGFL8
EGFR
EGLN1
EGLN2
EGLN3
EGR1
EGR2
EGR3
EHBP1
EHBP1L1
EHD1
EHD2
EHD3
EHD4
EHF
EHHADH
EHMT1
EHMT2
EI24
EID1
EID2
EID2B
EID3
EIF1
EIF1AD
EIF1AX
EIF1AY

EIF1B
EIF2A
EIF2AK1
EIF2AK2
EIF2AK3
EIF2AK4
EIF2B1
EIF2B2
EIF2B3
EIF2B4
EIF2B5
EIF2D
EIF2S1
EIF2S2
EIF2S2P4
EIF2S3
EIF2S3B
EIF3A
EIF3B
EIF3C
EIF3CL
EIF3D
EIF3E
EIF3F
EIF3FP3
EIF3G
EIF3H
EIF3I
EIF3J
EIF3K
EIF3L
EIF3M
EIF4A1
EIF4A2
EIF4A3
EIF4B
EIF4E
EIF4E2
EIF4E3
EIF4EBP1
EIF4EBP2
EIF4EBP3
EIF4ENIF1
EIF4EP1
EIF4EP2
EIF4G1
EIF4G2
EIF4G3
EIF4H
EIF5
EIF5A
EIF5A2
EIF5AL1
EIF5B
EIF6
EIPR1
ELAC1
ELAC2

ELAVL1
ELF1
ELF2
ELF3
ELF4
ELF5
ELFN1
ELK1
ELK3
ELK4
ELL
ELL2
ELL3
ELMO1
ELMO2
ELMO3
ELMOD1
ELMOD2
ELMOD3
ELN
ELOA
ELOA3D
ELOB
ELOC
ELOF1
ELOVL1
ELOVL5
ELOVL6
ELOVL7
ELP1
ELP2
ELP3
ELP4
ELP5
ELP6
EMB
EMBP1
EMC1
EMC10
EMC2
EMC3
EMC3-AS1
EMC4
EMC6
EMC7
EMC8
EMC9
EMCN
EMD
EME1
EME2
EMG1
EMILIN2
EML1
EML2
EML3
EML4
EML5

EML6
EMP1
EMP2
EMP3
EMSY
EMX2
ENAH
ENAM
ENC1
ENDOD1
ENDOG
ENDOV
ENG
ENGASE
ENKD1
ENKUR
ENO1
ENO1P1
ENO1P4
ENO2
ENO3
ENO4
ENOPH1
ENOSF1
ENOX1
ENOX2
ENPP2
ENPP3
ENPP4
ENPP5
ENPP6
ENSA
ENTPD1
ENTPD3
ENTPD4
ENTPD5
ENTPD6
ENTPD7
ENTR1
ENY2
EOGT
EOLA1
EOLA2
EOMES
EP300
EP400
EP400P1
EPAS1
EPB41
EPB41L1
EPB41L2
EPB41L3
EPB41L4A
EPB41L4B
EPB41L5
EPC1
EPC2
EPCAM

EPDR1
EPG5
EPHA1
EPHA10
EPHA2
EPHA4
EPHB1
EPHB2
EPHB3
EPHB4
EPHB6
EPHX1
EPHX2
EPHX3
EPM2AIP1
EPN1
EPN2
EPN3
EPOR
EPPIN
EPPIN-WFDC6
EPPK1
EPRS1
EPS15
EPS15L1
EPS15P1
EPS8
EPS8L1
EPS8L2
EPS8L3
EPSTI1
ERAL1
ERAP1
ERAP2
ERBB2
ERBB3
ERBB4
ERBIN
ERC1
ERC2
ERCC1
ERCC2
ERCC3
ERCC4
ERCC5
ERCC6
ERCC6L
ERCC6L2
ERCC8
EREG
ERF
ERG28
ERGIC1
ERGIC2
ERGIC3
ERH
ERI1
ERI2

ERI3
ERICH1
ERICH2
ERICH3
ERICH5
ERLEC1
ERLIN1
ERLIN2
ERMAP
ERMARD
ERMN
ERMP1
ERN1
ERN2
ERO1A
ERO1B
ERP27
ERP29
ERP44
ERRFI1
ERV3-1
ERVFRD-1
ERVK3-1
ERVW-1
ESCO1
ESCO2
ESD
ESF1
ESPL1
ESPN
ESPNP
ESR1
ESR2
ESRP1
ESRP2
ESRRA
ESRRG
ESS2
ESYT1
ESYT2
ESYT3
ETAA1
ETF1
ETF1P2
ETFA
ETFB
ETFBKMT
ETFDH
ETFRF1
ETHE1
ETNK1
ETNK2
ETS1
ETS2
ETV1
ETV3
ETV4
ETV5

ETV6
ETV7
EVA1B
EVA1C
EVC
EVC2
EVI2A
EVI2B
EVI5
EVI5L
EVL
EVPL
EWSR1
EXD2
EXD3
EXO1
EXO5
EXOC1
EXOC2
EXOC3
EXOC3L4
EXOC4
EXOC5
EXOC6
EXOC6B
EXOC7
EXOC8
EXOG
EXOSC1
EXOSC10
EXOSC2
EXOSC3
EXOSC4
EXOSC5
EXOSC6
EXOSC7
EXOSC8
EXOSC9
EXPH5
EXT1
EXT2
EXTL2
EXTL3
EYA1
EYA2
EYA3
EYA4
EYS
EZH1
EZH2
EZR
F11R
F13A1
F2R
F2RL1
F2RL2
F2RL3
F3

F5
F8
F8A1
F8A3
FA2H
FAAH
FAAH2
FAAP100
FAAP20
FAAP24
FABP2
FABP5
FABP5P7
FABP6
FABP7
FADD
FADS1
FADS2
FADS3
FADS6
FAF1
FAF2
FAH
FAHD1
FAHD2A
FAHD2B
FAHD2CP
FAIM
FAM102A
FAM102B
FAM104A
FAM104B
FAM107A
FAM107B
FAM110A
FAM110B
FAM110C
FAM110D
FAM111A
FAM111B
FAM114A1
FAM114A2
FAM117A
FAM117B
FAM118A
FAM118B
FAM120A
FAM120AOS
FAM120B
FAM120C
FAM122A
FAM122B
FAM122C
FAM124A
FAM124B
FAM126A
FAM126B
FAM131A

FAM133B
FAM135A
FAM135B
FAM136A
FAM13A
FAM13B
FAM13C
FAM149A
FAM149B1
FAM151B
FAM153A
FAM153B
FAM153CP
FAM155A
FAM156A
FAM156B
FAM157A
FAM157B
FAM160A1
FAM160A2
FAM160B1
FAM160B2
FAM161A
FAM161B
FAM162A
FAM163A
FAM166A
FAM166B
FAM166C
FAM167A
FAM168A
FAM168B
FAM169A
FAM169B
FAM171A1
FAM171B
FAM172A
FAM174A
FAM174B
FAM174C
FAM177A1
FAM177B
FAM178B
FAM180B
FAM181A
FAM181B
FAM183A
FAM184A
FAM184B
FAM185A
FAM186A
FAM186B
FAM187A
FAM189A2
FAM189B
FAM193A
FAM193B
FAM199X

FAM200A
FAM200B
FAM204A
FAM207A
FAM209A
FAM20A
FAM20B
FAM20C
FAM210A
FAM210B
FAM214A
FAM214B
FAM216A
FAM216B
FAM217B
FAM219A
FAM219B
FAM220A
FAM221A
FAM221B
FAM222A
FAM222B
FAM227A
FAM227B
FAM228B
FAM229A
FAM229B
FAM234A
FAM234B
FAM237B
FAM241A
FAM32A
FAM3A
FAM3B
FAM3C
FAM3C2
FAM3D
FAM43A
FAM47E
FAM47E-STBD1
FAM50A
FAM50B
FAM53B
FAM53C
FAM71A
FAM72A
FAM74A4
FAM74A6
FAM76A
FAM76B
FAM78A
FAM78B
FAM81A
FAM81B
FAM83A
FAM83B
FAM83C
FAM83D

FAM83E
FAM83F
FAM83G
FAM83H
FAM86B1
FAM86B3P
FAM86C1
FAM86C2P
FAM86DP
FAM86EP
FAM86JP
FAM89B
FAM8A1
FAM8A4P
FAM90A24P
FAM91A1
FAM92A
FAM92B
FAM95C
FAM98A
FAM98B
FAM98C
FAM9B
FAM9C
FAN1
FANCA
FANCC
FANCD2
FANCE
FANCF
FANCG
FANCI
FANCL
FANCM
FANK1
FAP
FAR1
FAR2
FAR2P1
FAR2P2
FARP1
FARP2
FARS2
FARSA
FARSB
FAS
FASLG
FASN
FASTK
FASTKD1
FASTKD2
FASTKD3
FASTKD5
FAT1
FAT2
FAT3
FAT4
FAU

FAXC
FAXDC2
FBF1
FBH1
FBL
FBLIM1
FBLN1
FBLN5
FBLN7
FBN1
FBN2
FBP1
FBRS
FBRSL1
FBXL12
FBXL13
FBXL14
FBXL15
FBXL16
FBXL17
FBXL18
FBXL19
FBXL2
FBXL20
FBXL22
FBXL3
FBXL4
FBXL5
FBXL6
FBXL7
FBXL8
FBXO10
FBXO11
FBXO15
FBXO16
FBXO17
FBXO2
FBXO21
FBXO22
FBXO24
FBXO25
FBXO27
FBXO28
FBXO3
FBXO30
FBXO31
FBXO32
FBXO33
FBXO34
FBXO36
FBXO38
FBXO39
FBXO4
FBXO40
FBXO41
FBXO42
FBXO43
FBXO44

FBXO45
FBXO46
FBXO48
FBXO5
FBXO6
FBXO7
FBXO8
FBXO9
FBXW10
FBXW11
FBXW12
FBXW2
FBXW4
FBXW4P1
FBXW5
FBXW7
FBXW8
FBXW9
FCAR
FCER1A
FCER1G
FCER2
FCF1
FCGBP
FCGR1A
FCGR1B
FCGR1CP
FCGR2A
FCGR2B
FCGR2C
FCGR3A
FCGR3B
FCGRT
FCHO1
FCHO2
FCHSD1
FCHSD2
FCMR
FCN1
FCRL1
FCRL2
FCRL3
FCRL4
FCRL5
FCRL6
FCRLA
FCSK
FDFT1
FDPS
FDPSP2
FDPSP3
FDPSP7
FDX1
FDX2
FDXACB1
FDXR
FECH
FEM1A

FEM1B
FEM1C
FEN1
FER
FER1L4
FER1L5
FER1L6
FERMT1
FERMT2
FERMT3
FES
FEZ1
FEZ2
FEZF1
FFAR1
FFAR2
FFAR4
FGD1
FGD2
FGD3
FGD4
FGD5
FGD6
FGF1
FGF11
FGF12
FGF13
FGF14
FGF23
FGF5
FGF7
FGF7P6
FGF9
FGFBP1
FGFBP3
FGFR1
FGFR1OP
FGFR1OP2
FGFR2
FGFR3
FGFRL1
FGGY
FGL2
FGR
FH
FHAD1
FHDC1
FHIT
FHL1
FHL2
FHL3
FHOD1
FHOD3
FIBP
FICD
FIG4
FIGN
FIGNL1

FILIP1
FILIP1L
FIP1L1
FIS1
FITM2
FIZ1
FKBP11
FKBP14
FKBP15
FKBP1A
FKBP1B
FKBP2
FKBP3
FKBP4
FKBP5
FKBP6
FKBP7
FKBP8
FKBP9
FKBP9P1
FKBPL
FKRP
FKTN
FLACC1
FLAD1
FLCN
FLG
FLG2
FLI1
FLII
FLNA
FLNB
FLNC
FLOT1
FLOT2
FLRT2
FLRT3
FLT1
FLT1P1
FLT3LG
FLT4
FLVCR1
FLVCR2
FLYWCH1
FLYWCH2
FMC1
FMC1-LUC7L2
FMN1
FMN2
FMNL1
FMNL2
FMNL3
FMO2
FMO3
FMO4
FMO5
FMO6P
FMR1

FN1
FN3K
FN3KRP
FNBP1
FNBP1L
FNBP1P1
FNBP4
FNDC10
FNDC11
FNDC3A
FNDC3B
FNDC9
FNIP1
FNIP2
FNTA
FNTB
FO681492.1
FOCAD
FOLH1
FOLR1
FOLR3
FOPNL
FOS
FOSB
FOSL1
FOSL2
FOXA1
FOXA3
FOXC1
FOXD4L5
FOXE1
FOXG1
FOXI1
FOXI2
FOXJ1
FOXJ2
FOXJ3
FOXK1
FOXK2
FOXL2NB
FOXM1
FOXN2
FOXN3
FOXN4
FOXO1
FOXO3
FOXO3B
FOXO4
FOXP1
FOXP2
FOXP3
FOXP4
FOXQ1
FOXRED1
FOXRED2
FP565260.1
FP565260.3
FP565260.6

FPGS
FPGT
FPGT-TNNI3K
FPR1
FPR2
FPR3
FRA10AC1
FRAS1
FRAT1
FRAT2
FREM1
FREM2
FRG1
FRG1BP
FRG1HP
FRK
FRMD3
FRMD4A
FRMD4B
FRMD5
FRMD6
FRMD8
FRMPD1
FRMPD2
FRMPD2B
FRMPD3
FRRS1
FRRS1L
FRS2
FRS3
FRY
FRYL
FSBP
FSCN1
FSD1L
FSD2
FSIP1
FSIP2
FSTL1
FSTL4
FSTL5
FTH1
FTL
FTO
FTSJ1
FTSJ3
FUBP1
FUBP3
FUCA1
FUCA2
FUNDC1
FUNDC2
FURIN
FUS
FUT1
FUT10
FUT11
FUT2

FUT3
FUT4
FUT6
FUT8
FUT9
FUZ
FXN
FXR1
FXR2
FXYD1
FXYD3
FXYD5
FXYD6
FYB1
FYB2
FYCO1
FYN
FYTTD1
FZD1
FZD3
FZD4
FZD5
FZD6
FZD8
FZR1
G0S2
G2E3
G3BP1
G3BP2
G6PC
G6PC3
G6PD
GAA
GAB1
GAB2
GAB3
GABARAP
GABARAPL1
GABARAPL2
GABBR1
GABPA
GABPAP
GABPB1
GABPB2
GABRA1
GABRA2
GABRA4
GABRB2
GABRB3
GABRE
GABRG3
GABRP
GAD1
GADD45A
GADD45B
GADD45G
GADD45GIP1
GADL1

GAK
GAL3ST2
GAL3ST4
GALC
GALE
GALK1
GALK2
GALM
GALNS
GALNT1
GALNT10
GALNT11
GALNT12
GALNT13
GALNT14
GALNT15
GALNT16
GALNT17
GALNT18
GALNT2
GALNT3
GALNT4
GALNT5
GALNT6
GALNT7
GALR1
GALT
GAMT
GAN
GANAB
GANC
GAPDH
GAPDHP24
GAPT
GAPVD1
GAR1
GAREM1
GAREM2
GARNL3
GARS1
GART
GAS1
GAS2
GAS2L1
GAS2L2
GAS2L3
GAS6
GAS7
GAS8
GASK1B
GATA3
GATA6
GATAD1
GATAD2A
GATAD2B
GATB
GATC
GATD1

GATD3A
GATD3B
GATM
GBA
GBA2
GBAP1
GBE1
GBF1
GBGT1
GBP1
GBP1P1
GBP2
GBP3
GBP4
GBP5
GBP6
GCA
GCAT
GCC1
GCC2
GCDH
GCFC2
GCH1
GCHFR
GCLC
GCLM
GCM1
GCN1
GCNA
GCNT1
GCNT1P1
GCNT2
GCNT3
GCNT4
GCNT7
GCOM1
GCSAM
GCSAML
GCSH
GDA
GDAP1
GDAP2
GDE1
GDF11
GDF15
GDF6
GDF7
GDF9
GDI1
GDI2
GDNF
GDPD1
GDPD3
GDPD5
GDPGP1
GEM
GEMIN2
GEMIN4

GEMIN5
GEMIN6
GEMIN7
GEMIN8
GEN1
GET1
GET3
GET4
GFAP
GFER
GFI1
GFI1B
GFM1
GFM2
GFOD1
GFOD2
GFPT1
GFRA1
GGA1
GGA2
GGA3
GGACT
GGCT
GGCX
GGH
GGNBP1
GGNBP2
GGPS1
GGT1
GGT5
GGT6
GGT7
GGTA1P
GHDC
GHITM
GHR
GID4
GID8
GIGYF1
GIGYF2
GIMAP1
GIMAP1-GIMAP5
GIMAP2
GIMAP4
GIMAP5
GIMAP6
GIMAP7
GIMAP8
GIN1
GINM1
GINS1
GINS2
GINS3
GINS4
GIPC1
GIPC2
GIPR
GIT1

GIT2
GJA9
GJB2
GJB3
GJB4
GJB5
GJB7
GJC1
GK
GK3P
GK5
GKAP1
GLA
GLB1
GLB1L
GLB1L2
GLB1L3
GLCCI1
GLCE
GLDN
GLE1
GLG1
GLI1
GLI2
GLI3
GLI4
GLIPR1
GLIPR1L2
GLIPR2
GLIS2
GLIS3
GLMN
GLMP
GLO1
GLOD4
GLRA3
GLRX
GLRX3
GLRX5
GLS
GLS2
GLT1D1
GLT8D1
GLTP
GLUD1
GLUL
GLYATL2
GLYCTK
GLYR1
GM2A
GMCL1
GMDS
GMEB1
GMEB2
GMFB
GMFG
GMIP
GMNC

GMNN
GMPPA
GMPPB
GMPR
GMPR2
GMPS
GNA11
GNA12
GNA13
GNA14
GNA15
GNAI1
GNAI2
GNAI3
GNAL
GNAO1
GNAQ
GNAS
GNAZ
GNB1
GNB1L
GNB2
GNB4
GNB5
GNE
GNG10
GNG12
GNG2
GNG4
GNG5
GNG7
GNGT1
GNGT2
GNL1
GNL2
GNL3
GNL3L
GNLY
GNPAT
GNPDA1
GNPDA2
GNPNAT1
GNPTAB
GNPTG
GNRH1
GNRHR2
GNS
GOLGA1
GOLGA2
GOLGA2P10
GOLGA2P11
GOLGA2P5
GOLGA2P7
GOLGA3
GOLGA4
GOLGA5
GOLGA6A
GOLGA6D

GOLGA6L10
GOLGA6L17P
GOLGA6L22
GOLGA6L4
GOLGA6L5P
GOLGA6L9
GOLGA7
GOLGA7B
GOLGA8A
GOLGA8B
GOLGA8J
GOLGA8N
GOLGA8Q
GOLGA8R
GOLGB1
GOLIM4
GOLM1
GOLPH3
GOLPH3L
GOLT1B
GON4L
GON7
GOPC
GORAB
GORASP1
GORASP2
GOSR1
GOSR2
GOT1
GOT2
GP2
GP5
GP6
GPAA1
GPALPP1
GPAM
GPANK1
GPAT2
GPAT3
GPAT4
GPATCH1
GPATCH11
GPATCH2
GPATCH2L
GPATCH3
GPATCH4
GPATCH8
GPBAR1
GPBP1
GPBP1L1
GPC1
GPC2
GPC4
GPCPD1
GPD1L
GPD2
GPHN
GPI

GPKOW
GPLD1
GPM6A
GPM6B
GPN1
GPN2
GPN3
GPNMB
GPR107
GPR108
GPR132
GPR135
GPR137
GPR137B
GPR137C
GPR141
GPR153
GPR155
GPR156
GPR157
GPR160
GPR161
GPR162
GPR171
GPR173
GPR18
GPR180
GPR183
GPR27
GPR34
GPR35
GPR37L1
GPR4
GPR61
GPR63
GPR65
GPR68
GPR75
GPR82
GPR83
GPR84
GPR85
GPR87
GPR89A
GPR89B
GPRASP1
GPRASP2
GPRC5A
GPRC5B
GPRC5C
GPRC5D
GPRIN3
GPS1
GPS2
GPSM2
GPSM3
GPT2
GPX1

GPX2
GPX3
GPX4
GPX6
GPX7
GPX8
GRAMD1A
GRAMD1B
GRAMD1C
GRAMD2A
GRAMD2B
GRAMD4
GRAMD4P3
GRAMD4P7
GRAP
GRAP2
GRASP
GRB10
GRB2
GRB7
GREB1
GREB1L
GREM1
GRHL1
GRHL2
GRHL3
GRHPR
GRIA1
GRIA4
GRIK2
GRIN2A
GRIN2B
GRIN2C
GRIN3A
GRIN3B
GRINA
GRIP1
GRIP2
GRIPAP1
GRK2
GRK3
GRK4
GRK5
GRK6
GRM2
GRM4
GRM5
GRM7
GRN
GRPEL1
GRPEL2
GRSF1
GRTP1
GRWD1
GSAP
GSDMA
GSDMB
GSDMC

GSDMD
GSDME
GSE1
GSG1
GSG1L
GSK3A
GSK3B
GSKIP
GSN
GSPT1
GSPT2
GSR
GSTA1
GSTA2
GSTA3
GSTA4
GSTA8P
GSTCD
GSTK1
GSTM1
GSTM2
GSTM3
GSTM4
GSTO1
GSTO2
GSTP1
GSTT1
GSTT4
GSTZ1
GTDC1
GTF2A1
GTF2A2
GTF2B
GTF2E1
GTF2E2
GTF2F1
GTF2F2
GTF2H1
GTF2H2
GTF2H2B
GTF2H2C
GTF2H2C_2
GTF2H3
GTF2H4
GTF2H5
GTF2I
GTF2IP1
GTF2IP12
GTF2IP13
GTF2IP20
GTF2IP4
GTF2IP7
GTF2IRD1
GTF2IRD1P1
GTF2IRD2
GTF2IRD2B
GTF2IRD2P1
GTF3A

GTF3C1
GTF3C2
GTF3C3
GTF3C4
GTF3C5
GTF3C6
GTPBP1
GTPBP10
GTPBP2
GTPBP3
GTPBP4
GTPBP6
GTPBP8
GTSE1
GTSF1
GUCA1B
GUCD1
GUCY1A1
GUCY1A2
GUCY1B1
GUCY1B2
GUCY2C
GUCY2F
GUF1
GUK1
GULP1
GUSB
GUSBP1
GUSBP2
GUSBP3
GUSBP6
GUSBP9
GVINP1
GVQW3
GXYLT1
GXYLT2
GYG1
GYG2
GYG2P1
GYPC
GYPE
GYS1
GZF1
GZMA
GZMB
GZMH
H1-0
H1-10
H1-2
H1-3
H1-4
H1-5
H2AC11
H2AC18
H2AC19
H2AC20
H2AC6
H2AC8

H2AJ
H2AX
H2AZ1
H2AZ2
H2BC11
H2BC15
H2BC18
H2BC20P
H2BC21
H2BC4
H2BC5
H2BC6
H2BC7
H2BC8
H2BC9
H2BU1
H3-3A
H3-3B
H3C10
H3C12
H3C14
H3C15
H3C6
H3C8
H3P37
H3P6
H4-16
H4C14
H4C15
H4C2
H4C3
H4C5
H4C8
H4C9
H6PD
HAAO
HABP4
HACD1
HACD2
HACD3
HACD4
HACE1
HACL1
HADH
HADHA
HADHB
HAGH
HAGHL
HAL
HAMP
HAP1
HAPLN3
HAPLN4
HARBI1
HARS1
HARS2
HAS2
HAS3

HASPIN
HAT1
HAUS1
HAUS2
HAUS3
HAUS4
HAUS5
HAUS6
HAUS7
HAUS8
HAVCR2
HAX1
HBA1
HBA2
HBB
HBEGF
HBG2
HBP1
HBS1L
HCAR1
HCAR2
HCAR3
HCCS
HCFC1
HCFC1R1
HCFC2
HCK
HCLS1
HCN1
HCN3
HCN4
HDAC1
HDAC10
HDAC11
HDAC1P2
HDAC2
HDAC3
HDAC4
HDAC5
HDAC6
HDAC7
HDAC8
HDAC9
HDC
HDDC2
HDDC3
HDGF
HDGFL2
HDGFL3
HDHD2
HDHD3
HDHD5
HDLBP
HDX
HEATR1
HEATR3
HEATR4
HEATR5A

HEATR5B
HEATR6
HEATR9
HEBP1
HEBP2
HECA
HECTD1
HECTD2
HECTD3
HECTD4
HECW1
HECW2
HEG1
HELB
HELLS
HELQ
HELZ
HELZ2
HEMGN
HEMK1
HENMT1
HEPACAM2
HEPHL1
HERC1
HERC2
HERC2P2
HERC2P3
HERC2P4
HERC2P5
HERC2P8
HERC2P9
HERC3
HERC4
HERC5
HERC6
HERPUD1
HERPUD2
HES1
HES2
HES4
HES6
HESX1
HEXA
HEXB
HEXD
HEXIM1
HEXIM2
HEY1
HEY2
HFE
HGD
HGF
HGH1
HGS
HGSNAT
HHAT
HHATL
HHEX

HHIP
HHIPL1
HHLA1
HHLA2
HHLA3
HIBADH
HIBCH
HIC1
HIC2
HID1
HIF1A
HIF1AN
HIF3A
HIGD1A
HIGD2A
HIKESHI
HILPDA
HINFP
HINT1
HINT2
HINT3
HIP1
HIP1R
HIPK1
HIPK2
HIPK3
HIRA
HIRIP3
HIVEP1
HIVEP2
HIVEP3
HJURP
HK1
HK2
HK3
HLA-A
HLA-B
HLA-C
HLA-DMA
HLA-DMB
HLA-DOA
HLA-DOB
HLA-DPA1
HLA-DPB1
HLA-DPB2
HLA-DQA1
HLA-DQA2
HLA-DQB1
HLA-DQB2
HLA-DRA
HLA-DRB1
HLA-DRB3
HLA-DRB4
HLA-DRB5
HLA-E
HLA-F
HLA-H
HLA-J

HLA-L
HLA-V
HLCS
HLF
HLTF
HLX
HM13
HMBOX1
HMBS
HMCES
HMCN1
HMCN2
HMG20A
HMG20B
HMGA1
HMGA2
HMGB1
HMGB1P3
HMGB1P39
HMGB1P5
HMGB1P6
HMGB1P8
HMGB2
HMGB3
HMGB3P9
HMGCL
HMGCR
HMGCS1
HMGCS2
HMGN1
HMGN2
HMGN2P46
HMGN3
HMGN4
HMGN5
HMGXB3
HMGXB4
HMMR
HMOX1
HMOX2
HNF1B
HNF4A
HNF4G
HNMT
HNRNPA0
HNRNPA1
HNRNPA1L2
HNRNPA1P40
HNRNPA1P48
HNRNPA1P54
HNRNPA2B1
HNRNPA3
HNRNPA3P15
HNRNPA3P2
HNRNPA3P6
HNRNPAB
HNRNPC
HNRNPD

HNRNPDL
HNRNPF
HNRNPH1
HNRNPH1P1
HNRNPH2
HNRNPH3
HNRNPK
HNRNPL
HNRNPLL
HNRNPM
HNRNPR
HNRNPU
HNRNPUL1
HNRNPUL2
HNRNPUL2-BSCL2
HOGA1
HOMER1
HOMER2
HOMER3
HOMEZ
HOOK1
HOOK2
HOOK3
HOPX
HORMAD1
HOXB13
HOXB3
HP
HP1BP3
HPCAL1
HPF1
HPGD
HPRT1
HPS1
HPS3
HPS4
HPS5
HPS6
HPSE
HPX
HR
HRAS
HRG
HRH1
HRH2
HRH4
HRK
HRNR
HS1BP3
HS2ST1
HS3ST1
HS3ST2
HS3ST3B1
HS3ST6
HS6ST1
HSBP1
HSBP1L1
HSCB

HSD11B1L
HSD11B2
HSD17B1
HSD17B10
HSD17B11
HSD17B12
HSD17B13
HSD17B2
HSD17B4
HSD17B7
HSD17B7P2
HSD17B8
HSD3B7
HSDL1
HSDL2
HSF1
HSF2
HSF4
HSF5
HSH2D
HSP90AA1
HSP90AB1
HSP90AB2P
HSP90AB3P
HSP90AB4P
HSP90AB7P
HSP90B1
HSPA12A
HSPA13
HSPA14
HSPA1A
HSPA1B
HSPA1L
HSPA2
HSPA4
HSPA4L
HSPA5
HSPA6
HSPA7
HSPA8
HSPA9
HSPB1
HSPB11
HSPB8
HSPBAP1
HSPBP1
HSPD1
HSPE1
HSPE1-MOB4
HSPG2
HSPH1
HTATIP2
HTATSF1
HTATSF1P2
HTD2
HTN1
HTN3
HTR1D

HTR2A
HTR3B
HTR7P1
HTRA1
HTRA2
HTRA4
HTT
HUNK
HUS1
HUWE1
HVCN1
HYAL1
HYAL2
HYAL4
HYDIN
HYDIN2
HYI
HYKK
HYLS1
HYOU1
HYPK
IAH1
IAPP
IARS1
IARS2
IARS2P1
IBA57
IBTK
ICA1
ICA1L
ICAM1
ICAM2
ICAM3
ICAM4
ICAM5
ICE1
ICE2
ICMT
ICOS
ICOSLG
ID1
ID2
ID3
ID4
IDE
IDH1
IDH2
IDH3A
IDH3B
IDH3G
IDI1
IDNK
IDO1
IDO2
IDS
IDUA
IER2
IER3

IER3IP1
IER5
IER5L
IFFO1
IFFO2
IFI16
IFI27
IFI27L2
IFI30
IFI35
IFI44
IFI44L
IFI6
IFIH1
IFIT1
IFIT1B
IFIT2
IFIT3
IFIT5
IFITM1
IFITM10
IFITM2
IFITM3
IFNAR1
IFNAR2
IFNG
IFNGR1
IFNGR2
IFNLR1
IFRD1
IFRD2
IFT122
IFT140
IFT172
IFT20
IFT22
IFT27
IFT43
IFT46
IFT52
IFT57
IFT74
IFT80
IFT81
IFT88
IFTAP
IGBP1
IGF1
IGF1R
IGF2
IGF2BP1
IGF2BP2
IGF2BP3
IGF2R
IGFBP2
IGFBP3
IGFBP4
IGFBP5

IGFBP7
IGFBPL1
IGFL2
IGFL4
IGFLR1
IGHA1
IGHA2
IGHD
IGHEP2
IGHG1
IGHG2
IGHG3
IGHG4
IGHGP
IGHM
IGHMBP2
IGIP
IGKC
IGKV1-33
IGKV1-39
IGKV1-5
IGKV1D-33
IGKV2-28
IGKV2D-28
IGKV3-11
IGKV3-15
IGKV3-20
IGKV4-1
IGLC1
IGLC2
IGLC3
IGLL5
IGLON5
IGLV1-44
IGLV1-51
IGLV2-14
IGLV2-23
IGLV2-8
IGLV3-1
IGLV3-21
IGSF1
IGSF10
IGSF11
IGSF3
IGSF5
IGSF6
IGSF8
IGSF9
IK
IKBIP
IKBKB
IKBKE
IKBKG
IKBKGP1
IKZF1
IKZF2
IKZF3
IKZF4

IKZF5
IL10RA
IL10RB
IL11RA
IL12A
IL12B
IL12RB1
IL12RB2
IL13RA1
IL15
IL15RA
IL16
IL17RA
IL17RB
IL17RC
IL17RD
IL17RE
IL17REL
IL18
IL18BP
IL18R1
IL18RAP
IL19
IL1A
IL1B
IL1R1
IL1R2
IL1RAP
IL1RL1
IL1RL2
IL1RN
IL20RA
IL20RB
IL21R
IL22RA1
IL23A
IL23R
IL24
IL27RA
IL2RA
IL2RB
IL2RG
IL32
IL33
IL36G
IL3RA
IL4I1
IL4R
IL5RA
IL6
IL6R
IL6ST
IL7
IL7R
ILDR1
ILDR2
ILF2
ILF3

ILK
ILKAP
ILRUN
ILVBL
IMMP1L
IMMP2L
IMMT
IMP3
IMP4
IMPA1
IMPA2
IMPACT
IMPAD1
IMPDH1
IMPDH1P10
IMPDH2
IMPG1
IMPG2
INA
INAVA
INCA1
INCENP
INF2
ING1
ING2
ING3
ING4
ING5
INHBA
INHBB
INHBE
INIP
INKA2
INMT
INMT-MINDY4
INO80
INO80B
INO80B-WBP1
INO80C
INO80D
INO80E
INPP1
INPP4A
INPP4B
INPP5A
INPP5B
INPP5D
INPP5E
INPP5F
INPP5J
INPP5K
INPPL1
INSIG1
INSIG2
INSL3
INSL4
INSL6
INSR

INSYN1
INSYN2A
INSYN2B
INTS1
INTS10
INTS11
INTS12
INTS13
INTS14
INTS2
INTS3
INTS4
INTS4P1
INTS4P2
INTS5
INTS6
INTS6L
INTS7
INTS8
INTS9
INTU
INVS
IP6K1
IP6K2
IP6K3
IPCEF1
IPMK
IPO11
IPO13
IPO4
IPO5
IPO5P1
IPO7
IPO7P2
IPO8
IPO9
IPP
IPPK
IQANK1
IQCA1
IQCB1
IQCC
IQCD
IQCE
IQCF3
IQCG
IQCH
IQCK
IQCM
IQCN
IQGAP1
IQGAP2
IQGAP3
IQSEC1
IQSEC2
IQUB
IRAK1
IRAK1BP1

IRAK2
IRAK3
IRAK4
IREB2
IRF1
IRF2
IRF2BP1
IRF2BP2
IRF2BPL
IRF3
IRF4
IRF5
IRF6
IRF7
IRF8
IRF9
IRGM
IRGQ
IRS1
IRS2
IRS3P
IRX3
IRX5
ISCA1
ISCA2
ISCU
ISG15
ISG20
ISG20L2
ISL1
ISLR2
ISOC1
ISOC2
IST1
ISY1
ISY1-RAB43
ISYNA1
ITCH
ITFG1
ITFG2
ITGA1
ITGA10
ITGA11
ITGA2
ITGA3
ITGA4
ITGA5
ITGA6
ITGA7
ITGA8
ITGA9
ITGAD
ITGAE
ITGAL
ITGAM
ITGAV
ITGAX
ITGB1

ITGB1BP1
ITGB2
ITGB3BP
ITGB4
ITGB5
ITGB6
ITGB7
ITGB8
ITGBL1
ITIH4
ITIH5
ITIH6
ITK
ITM2A
ITM2B
ITM2C
ITPA
ITPK1
ITPKB
ITPKC
ITPR1
ITPR2
ITPR3
ITPRID1
ITPRID2
ITPRIP
ITPRIPL1
ITPRIPL2
ITSN1
ITSN2
IVD
IVL
IVNS1ABP
IWS1
IYD
IZUMO1
IZUMO2
IZUMO4
JADE1
JADE2
JADE3
JAG1
JAG2
JAGN1
JAK1
JAK2
JAK3
JAKMIP2
JAKMIP3
JAM2
JAM3
JAML
JARID2
JAZF1
JCAD
JCHAIN
JDP2
JHY

JKAMP
JMJD1C
JMJD4
JMJD6
JMJD7
JMJD7-PLA2G4B
JMJD8
JMY
JOSD1
JOSD2
JPH1
JPH3
JPT1
JPT2
JRK
JRKL
JSRP1
JTB
JUN
JUNB
JUND
JUP
KALRN
KANK1
KANK2
KANSL1
KANSL1L
KANSL2
KANSL3
KANTR
KARS1
KAT14
KAT2A
KAT2B
KAT5
KAT6A
KAT6B
KAT7
KAT8
KATNA1
KATNAL1
KATNAL2
KATNB1
KATNBL1
KAZN
KBTBD11
KBTBD12
KBTBD2
KBTBD3
KBTBD4
KBTBD6
KBTBD7
KBTBD8
KCMF1
KCNA1
KCNA3
KCNA7
KCNAB1

KCNAB2
KCNAB3
KCNB1
KCNC3
KCNC4
KCND1
KCND3
KCNE1
KCNE1B
KCNE3
KCNE4
KCNG1
KCNG4
KCNH3
KCNH6
KCNH7
KCNIP4
KCNJ1
KCNJ13
KCNJ14
KCNJ15
KCNJ16
KCNJ2
KCNJ3
KCNJ5
KCNJ6
KCNK1
KCNK10
KCNK12
KCNK2
KCNK3
KCNK5
KCNK6
KCNMA1
KCNMB1
KCNMB2
KCNMB3
KCNMB4
KCNN2
KCNN3
KCNN4
KCNQ1
KCNQ3
KCNQ5
KCNRG
KCNS1
KCNS3
KCNT1
KCNV1
KCP
KCTD1
KCTD10
KCTD11
KCTD12
KCTD13
KCTD14
KCTD15
KCTD17

KCTD18
KCTD19
KCTD2
KCTD20
KCTD21
KCTD3
KCTD5
KCTD6
KCTD7
KCTD9
KDELR1
KDELR2
KDELR3
KDF1
KDM1A
KDM1B
KDM2A
KDM2B
KDM3A
KDM3B
KDM4A
KDM4B
KDM4C
KDM4D
KDM5A
KDM5B
KDM5C
KDM5D
KDM6A
KDM6B
KDM7A
KDM8
KDSR
KEAP1
KHDC1
KHDC4
KHDRBS1
KHDRBS3
KHNYN
KHSRP
KIAA0040
KIAA0100
KIAA0232
KIAA0319
KIAA0319L
KIAA0355
KIAA0408
KIAA0513
KIAA0556
KIAA0586
KIAA0753
KIAA0825
KIAA0895
KIAA0895L
KIAA0930
KIAA1109
KIAA1143
KIAA1191

KIAA1211L
KIAA1217
KIAA1257
KIAA1324
KIAA1324L
KIAA1328
KIAA1522
KIAA1549
KIAA1549L
KIAA1586
KIAA1614
KIAA1671
KIAA1841
KIAA1958
KIAA2012
KIAA2013
KIAA2026
KIDINS220
KIF11
KIF13A
KIF13B
KIF14
KIF15
KIF16B
KIF17
KIF18A
KIF18B
KIF19
KIF1B
KIF1C
KIF20A
KIF20B
KIF21A
KIF21B
KIF22
KIF23
KIF24
KIF26B
KIF27
KIF28P
KIF2A
KIF2C
KIF3A
KIF3B
KIF3C
KIF4A
KIF5B
KIF5C
KIF6
KIF9
KIFAP3
KIFBP
KIFC1
KIFC2
KIFC3
KIN
KIR3DX1
KIRREL3

KIT
KITLG
KIZ
KLB
KLC1
KLC2
KLC3
KLC4
KLF10
KLF11
KLF12
KLF13
KLF15
KLF16
KLF17
KLF2
KLF2P4
KLF3
KLF4
KLF5
KLF6
KLF7
KLF8
KLF9
KLHDC1
KLHDC10
KLHDC2
KLHDC3
KLHDC4
KLHDC7A
KLHDC7B
KLHDC8A
KLHDC8B
KLHDC9
KLHL11
KLHL12
KLHL13
KLHL14
KLHL15
KLHL17
KLHL18
KLHL2
KLHL20
KLHL21
KLHL22
KLHL23
KLHL24
KLHL25
KLHL26
KLHL28
KLHL29
KLHL3
KLHL30
KLHL31
KLHL32
KLHL33
KLHL35
KLHL36

KLHL41
KLHL42
KLHL5
KLHL6
KLHL7
KLHL8
KLHL9
KLK10
KLK11
KLK13
KLK6
KLK7
KLKB1
KLLN
KLRA1P
KLRB1
KLRC1
KLRC2
KLRC4-KLRK1
KLRD1
KLRF1
KLRG1
KLRK1
KMO
KMT2A
KMT2B
KMT2C
KMT2D
KMT2E
KMT5A
KMT5B
KMT5C
KNDC1
KNL1
KNOP1
KNSTRN
KNTC1
KPNA1
KPNA2
KPNA2P1
KPNA2P3
KPNA3
KPNA4
KPNA5
KPNA6
KPNA7
KPNB1
KPTN
KRAS
KRBA1
KRBA2
KRBOX4
KRCC1
KREMEN1
KRI1
KRIT1
KRR1
KRT10

KRT13
KRT14
KRT15
KRT16
KRT17
KRT18
KRT18P31
KRT18P57
KRT19
KRT23
KRT24
KRT39
KRT4
KRT40
KRT42P
KRT5
KRT6A
KRT6B
KRT7
KRT78
KRT8
KRT80
KRT87P
KRT8P12
KRT8P15
KRT8P33
KRT8P43
KRT8P46
KRTCAP2
KRTCAP3
KSR1
KSR2
KTI12
KTN1
KXD1
KY
KYAT1
KYAT3
KYNU
L1TD1
L2HGDH
L3HYPDH
L3MBTL1
L3MBTL2
L3MBTL3
L3MBTL4
LACC1
LACTB
LACTB2
LAD1
LAG3
LAGE3
LAIR1
LAMA1
LAMA2
LAMA3
LAMA4
LAMA5

LAMB1
LAMB2
LAMB3
LAMB4
LAMC1
LAMC2
LAMC3
LAMP1
LAMP2
LAMP3
LAMTOR1
LAMTOR2
LAMTOR3
LAMTOR4
LAMTOR5
LANCL1
LANCL2
LANCL3
LAP3
LAPTM4A
LAPTM4B
LAPTM5
LARGE1
LARGE2
LARP1
LARP1B
LARP4
LARP4B
LARP6
LARP7
LARS1
LARS2
LAS1L
LASP1
LAT
LAT2
LATS1
LATS2
LAX1
LAYN
LBH
LBHD1
LBR
LCA5
LCA5L
LCAT
LCK
LCLAT1
LCMT1
LCMT2
LCN15
LCN2
LCOR
LCORL
LCP1
LCP2
LCT
LCTL

LDAH
LDB1
LDB3
LDHA
LDHAL6A
LDHAL6B
LDHAL6CP
LDHB
LDHBP1
LDHC
LDHD
LDLR
LDLRAD1
LDLRAD3
LDLRAD4
LDLRAP1
LDOC1
LEAP2
LECT2
LEF1
LEKR1
LEMD2
LEMD3
LENG1
LENG8
LENG9
LEO1
LEPR
LEPROT
LEPROTL1
LETM1
LETM2
LETMD1
LFNG
LGALS1
LGALS3
LGALS3BP
LGALS8
LGALS9
LGALS9B
LGALS9C
LGALSL
LGI1
LGMN
LGR4
LGR5
LGR6
LGSN
LHFPL2
LHFPL5
LHFPL6
LHPP
LHX4
LIAS
LIF
LIFR
LIG1
LIG3

LIG4
LILRA1
LILRA2
LILRA3
LILRA4
LILRA5
LILRA6
LILRB1
LILRB2
LILRB3
LILRB4
LILRB5
LIMA1
LIMCH1
LIMD1
LIMD2
LIME1
LIMK1
LIMK2
LIMS1
LIMS3
LIN28A
LIN28B
LIN37
LIN52
LIN54
LIN7A
LIN7B
LIN7C
LIN9
LINC00643
LINC00672
LINC00674
LINC00680
LINC00869
LINC00888
LINC00933
LINC01145
LINC01347
LINC01667
LINC01881
LINC02210
LINC02495
LINC02618
LINC02693
LINGO2
LINGO3
LINS1
LIPA
LIPC
LIPE
LIPG
LIPH
LIPN
LIPT1
LIPT2
LITAF
LITAFD

LIX1
LIX1L
LLGL1
LLGL2
LLPH
LMAN1
LMAN2
LMAN2L
LMBR1
LMBR1L
LMBRD1
LMBRD2
LMCD1
LMF1
LMF2
LMLN
LMNA
LMNB1
LMNB2
LMNTD1
LMNTD2
LMO2
LMO3
LMO4
LMO7
LMOD3
LMTK2
LMX1B
LNPEP
LNPK
LNX1
LNX2
LONP1
LONP2
LONRF1
LONRF2
LONRF3
LOX
LOXHD1
LOXL1
LOXL2
LOXL3
LOXL4
LPA
LPAL2
LPAR1
LPAR2
LPAR3
LPAR5
LPAR6
LPCAT1
LPCAT2
LPCAT3
LPCAT4
LPGAT1
LPIN1
LPIN2
LPIN3

LPO
LPP
LPXN
LRAT
LRATD1
LRATD2
LRBA
LRCH1
LRCH3
LRCH4
LRFN1
LRFN3
LRFN4
LRG1
LRGUK
LRIF1
LRIG1
LRIG2
LRIG3
LRMDA
LRMP
LRP1
LRP10
LRP11
LRP12
LRP1B
LRP2
LRP2BP
LRP3
LRP4
LRP5
LRP5L
LRP6
LRP8
LRPAP1
LRPPRC
LRR1
LRRC1
LRRC10
LRRC10B
LRRC14
LRRC17
LRRC18
LRRC19
LRRC2
LRRC20
LRRC23
LRRC25
LRRC27
LRRC28
LRRC29
LRRC34
LRRC36
LRRC37A
LRRC37A11P
LRRC37A15P
LRRC37A16P
LRRC37A17P

LRRC37A2
LRRC37A3
LRRC37A4P
LRRC37A5P
LRRC37A6P
LRRC37A7P
LRRC37A9P
LRRC37B
LRRC37BP1
LRRC39
LRRC4
LRRC40
LRRC41
LRRC42
LRRC43
LRRC45
LRRC46
LRRC47
LRRC49
LRRC56
LRRC57
LRRC58
LRRC59
LRRC6
LRRC61
LRRC63
LRRC66
LRRC69
LRRC70
LRRC71
LRRC73
LRRC74B
LRRC75A
LRRC8A
LRRC8B
LRRC8C
LRRC8D
LRRC8E
LRRCC1
LRRD1
LRRFIP1
LRRFIP1P1
LRRFIP2
LRRIQ1
LRRIQ3
LRRK1
LRRK2
LRRN1
LRRN2
LRRN3
LRRN4CL
LRRTM2
LRRTM4
LRSAM1
LRTOMT
LRWD1
LSAMP
LSG1

LSM1
LSM10
LSM11
LSM12
LSM12P1
LSM14A
LSM14B
LSM2
LSM3
LSM4
LSM5
LSM6
LSM7
LSM8
LSMEM1
LSP1
LSP1P4
LSR
LSS
LST1
LTA
LTA4H
LTB
LTB4R
LTB4R2
LTBP1
LTBP2
LTBP3
LTBP4
LTBR
LTF
LTK
LTN1
LTO1
LTV1
LUC7L
LUC7L2
LUC7L3
LURAP1
LURAP1L
LUZP1
LUZP2
LXN
LY6D
LY6E
LY6G5B
LY6G5C
LY6K
LY75
LY75-CD302
LY86
LY9
LY96
LYAR
LYL1
LYN
LYNX1
LYPD2

LYPD3
LYPD5
LYPD6
LYPD6B
LYPD9P
LYPLA1
LYPLA2
LYPLAL1
LYRM1
LYRM2
LYRM4
LYRM7
LYRM9
LYSMD1
LYSMD2
LYSMD3
LYSMD4
LYST
LYVE1
LYZ
LZIC
LZTFL1
LZTR1
LZTS1
LZTS2
LZTS3
M1AP
M6PR
MAATS1
MAB21L1
MAB21L2
MAB21L3
MAB21L4
MACC1
MACF1
MACIR
MACO1
MACROD1
MACROD2
MACROH2A1
MACROH2A2
MAD1L1
MAD2L1
MAD2L1BP
MAD2L2
MADD
MAEA
MAF
MAF1
MAFB
MAFF
MAFG
MAFK
MAGEA10
MAGED1
MAGED2
MAGEF1
MAGEH1

MAGI1
MAGI2
MAGI3
MAGIX
MAGOH
MAGOHB
MAGT1
MAIP1
MAK
MAK16
MAL
MAL2
MALL
MALSU1
MALT1
MAMDC2
MAMDC4
MAML1
MAML2
MAML3
MAMLD1
MAN1A1
MAN1A2
MAN1B1
MAN1C1
MAN2A1
MAN2A2
MAN2B1
MAN2B2
MAN2C1
MANBA
MANBAL
MANEA
MANEAL
MANF
MANSC1
MAOA
MAOB
MAP10
MAP11
MAP1A
MAP1B
MAP1LC3A
MAP1LC3B
MAP1LC3B2
MAP1S
MAP2
MAP2K1
MAP2K2
MAP2K3
MAP2K4
MAP2K5
MAP2K6
MAP2K7
MAP3K1
MAP3K10
MAP3K11
MAP3K12

MAP3K13
MAP3K14
MAP3K15
MAP3K19
MAP3K2
MAP3K20
MAP3K21
MAP3K3
MAP3K4
MAP3K5
MAP3K6
MAP3K7
MAP3K7CL
MAP3K8
MAP3K9
MAP4
MAP4K1
MAP4K2
MAP4K3
MAP4K4
MAP4K5
MAP6
MAP7
MAP7D1
MAP7D2
MAP7D3
MAP9
MAPK1
MAPK10
MAPK12
MAPK13
MAPK14
MAPK15
MAPK1IP1L
MAPK3
MAPK6
MAPK6P3
MAPK7
MAPK8
MAPK8IP1
MAPK8IP2
MAPK8IP3
MAPK9
MAPKAP1
MAPKAPK2
MAPKAPK3
MAPKAPK5
MAPKBP1
MAPRE1
MAPRE2
MAPRE3
MAPT
MARCHF1
MARCHF10
MARCHF2
MARCHF3
MARCHF4
MARCHF5

MARCHF6
MARCHF7
MARCHF8
MARCHF9
MARCKS
MARCKSL1
MARCKSL1P2
MARF1
MARK1
MARK2
MARK2P8
MARK3
MARK4
MARS1
MARS2
MARVELD1
MARVELD2
MARVELD3
MAS1
MASP1
MASP2
MAST1
MAST2
MAST3
MAST4
MASTL
MAT1A
MAT2A
MAT2B
MATK
MATN1
MATN2
MATR3
MAU2
MAVS
MAX
MAZ
MB
MB21D2
MBD1
MBD2
MBD3
MBD3L1
MBD4
MBD5
MBD6
MBIP
MBLAC2
MBNL1
MBNL2
MBNL3
MBOAT1
MBOAT2
MBOAT7
MBP
MBTD1
MBTPS1
MBTPS2

MC1R
MC2R
MCAM
MCAT
MCC
MCCC1
MCCC2
MCEE
MCEMP1
MCF2L
MCF2L2
MCFD2
MCIDAS
MCL1
MCM10
MCM2
MCM3
MCM3AP
MCM4
MCM5
MCM6
MCM7
MCM8
MCM9
MCMBP
MCMDC2
MCOLN1
MCOLN2
MCOLN3
MCPH1
MCRIP1
MCRIP2
MCRS1
MCTP1
MCTP2
MCTS1
MCU
MCUB
MCUR1
MDC1
MDFI
MDFIC
MDGA1
MDH1
MDH1B
MDH2
MDK
MDM1
MDM2
MDM4
MDN1
ME1
ME2
ME3
MEA1
MEAF6
MEAK7
MECOM

MECP2
MECR
MED1
MED10
MED11
MED12
MED12L
MED13
MED13L
MED14
MED15
MED15P9
MED16
MED17
MED18
MED19
MED20
MED21
MED22
MED23
MED24
MED25
MED26
MED27
MED28
MED29
MED30
MED31
MED4
MED6
MED7
MED8
MED9
MEF2A
MEF2C
MEF2D
MEFV
MEGF10
MEGF11
MEGF6
MEGF8
MEGF9
MEI1
MEIG1
MEIOC
MEIS1
MEIS2
MEIS3
MEIS3P1
MEIS3P2
MELK
MELTF
MEMO1
MEN1
MEP1A
MEPCE
MERTK
MESD

MEST
MET
METAP1
METAP1D
METAP2
METRN
METRNL
METTL1
METTL14
METTL15
METTL15P1
METTL16
METTL17
METTL18
METTL21A
METTL21EP
METTL22
METTL23
METTL25
METTL26
METTL27
METTL2A
METTL2B
METTL3
METTL4
METTL5
METTL6
METTL7A
METTL8
METTL9
MEX3A
MEX3C
MEX3D
MFAP1
MFAP2
MFAP3
MFAP3L
MFAP5
MFF
MFGE8
MFHAS1
MFN1
MFN2
MFNG
MFSD1
MFSD10
MFSD11
MFSD12
MFSD13A
MFSD14A
MFSD14B
MFSD14C
MFSD1P1
MFSD2A
MFSD2B
MFSD3
MFSD4A
MFSD4B

MFSD5
MFSD6
MFSD8
MFSD9
MGA
MGAM
MGAM2
MGAT1
MGAT2
MGAT3
MGAT4A
MGAT4B
MGAT4C
MGAT5
MGLL
MGME1
MGMT
MGP
MGRN1
MGST1
MGST2
MGST3
MIA-RAB4B
MIA2
MIA3
MIB1
MIB2
MICA
MICAL1
MICAL2
MICAL3
MICALL1
MICALL2
MICB
MICOS10
MICOS10-NBL1
MICOS13
MICU1
MICU2
MICU3
MID1
MID1IP1
MID2
MIDEAS
MIDN
MIEF1
MIEF2
MIEN1
MIER1
MIER2
MIER3
MIF
MIF4GD
MIGA1
MIGA2
MIIP
MILR1
MINAR1

MINDY1
MINDY2
MINDY3
MINDY4
MINK1
MINPP1
MIOS
MIPEP
MIPOL1
MIR1915HG
MIS12
MIS18A
MIS18BP1
MISP
MISP3
MITD1
MITF
MIXL1
MKI67
MKKS
MKLN1
MKNK1
MKNK2
MKRN1
MKRN2
MKRN2OS
MKRN3
MKRN5P
MKS1
MKX
MLANA
MLEC
MLF1
MLF2
MLH1
MLH3
MLIP
MLKL
MLLT1
MLLT10
MLLT11
MLLT3
MLLT6
MLPH
MLST8
MLX
MLXIP
MLXIPL
MLYCD
MMAA
MMAB
MMACHC
MMADHC
MMD
MME
MMGT1
MMP1
MMP10

MMP12
MMP13
MMP14
MMP15
MMP19
MMP24
MMP24OS
MMP25
MMP7
MMP8
MMP9
MMRN2
MMS19
MMS22L
MMUT
MNAT1
MND1
MNDA
MNS1
MNT
MOAP1
MOB1A
MOB1B
MOB2
MOB3A
MOB3B
MOB3C
MOB4
MOBP
MOCOS
MOCS2
MOCS3
MOG
MOGAT3
MOGS
MOK
MON1A
MON1B
MON2
MORC2
MORC3
MORC4
MORF4L1
MORF4L2
MORN1
MORN2
MORN3
MORN4
MORN5
MOSMO
MOSPD1
MOSPD2
MOSPD3
MOV10
MOV10L1
MPC1
MPC2
MPDU1

MPDZ
MPEG1
MPG
MPHOSPH10
MPHOSPH6
MPHOSPH8
MPHOSPH9
MPI
MPIG6B
MPLKIP
MPND
MPP1
MPP4
MPP5
MPP6
MPP7
MPPE1
MPPED2
MPRIP
MPST
MPV17
MPV17L
MPV17L2
MPZL1
MPZL2
MPZL3
MR1
MRAP2
MRAS
MRC1
MRC2
MRE11
MREG
MRFAP1
MRFAP1L1
MRGBP
MRI1
MRLN
MRM2
MRM3
MRNIP
MRO
MROH1
MROH3P
MROH6
MROH7
MROH7-TTC4
MROH8
MROH9
MRPL1
MRPL10
MRPL11
MRPL12
MRPL13
MRPL14
MRPL15
MRPL16
MRPL17

MRPL18
MRPL19
MRPL2
MRPL20
MRPL21
MRPL22
MRPL23
MRPL24
MRPL27
MRPL28
MRPL3
MRPL30
MRPL32
MRPL33
MRPL34
MRPL35
MRPL36
MRPL37
MRPL38
MRPL39
MRPL4
MRPL40
MRPL41
MRPL42
MRPL42P6
MRPL43
MRPL44
MRPL45
MRPL45P2
MRPL46
MRPL47
MRPL48
MRPL49
MRPL50
MRPL51
MRPL52
MRPL53
MRPL54
MRPL55
MRPL57
MRPL58
MRPL9
MRPS10
MRPS11
MRPS12
MRPS14
MRPS15
MRPS16
MRPS17
MRPS17P1
MRPS18A
MRPS18B
MRPS18C
MRPS2
MRPS21
MRPS22
MRPS23
MRPS24

MRPS25
MRPS26
MRPS27
MRPS28
MRPS30
MRPS31
MRPS31P4
MRPS31P5
MRPS33
MRPS34
MRPS35
MRPS36
MRPS5
MRPS6
MRPS7
MRPS9
MRRF
MRS2
MRTFA
MRTFB
MRTO4
MRVI1
MS4A1
MS4A10
MS4A14
MS4A2
MS4A4A
MS4A6A
MS4A7
MS4A8
MSANTD1
MSANTD2
MSANTD3
MSANTD4
MSH2
MSH3
MSH5
MSH5-SAPCD1
MSH6
MSI2
MSL1
MSL2
MSL3
MSL3P1
MSLN
MSMB
MSMO1
MSN
MSR1
MSRA
MSRB1
MSRB2
MSRB3
MSS51
MST1
MST1L
MST1R
MSTO1

MT-ATP6
MT-CO1
MT-CO2
MT-CO3
MT-CYB
MT-ND1
MT-ND2
MT-ND3
MT-ND4
MT-ND4L
MT-ND5
MT-ND6
MT1E
MT1F
MT1X
MT2A
MT3
MTA1
MTA2
MTA3
MTAP
MTARC1
MTARC2
MTATP6P1
MTATP6P11
MTATP6P2
MTATP6P26
MTBP
MTCH1
MTCH2
MTCL1
MTCO1P1
MTCO1P11
MTCO1P12
MTCO1P15
MTCO1P2
MTCO1P25
MTCO1P40
MTCO1P42
MTCO1P53
MTCO2P12
MTCO2P22
MTCO3P12
MTCO3P13
MTCO3P22
MTCO3P43
MTCYBP18
MTCYBP23
MTCYBP3
MTCYBP35
MTDH
MTERF1
MTERF2
MTERF3
MTERF4
MTF1
MTF2
MTFMT

MTFP1
MTFR1
MTFR1L
MTFR2
MTG1
MTG2
MTHFD1
MTHFD1L
MTHFD1P1
MTHFD2
MTHFD2L
MTHFD2P1
MTHFR
MTHFS
MTHFSD
MTIF2
MTIF3
MTLN
MTM1
MTMR1
MTMR10
MTMR11
MTMR12
MTMR14
MTMR2
MTMR3
MTMR4
MTMR6
MTMR7
MTMR8
MTMR9
MTMR9LP
MTND1P11
MTND1P23
MTND1P8
MTND2P12
MTND2P28
MTND2P5
MTND2P9
MTND4P12
MTND4P14
MTND4P16
MTND4P22
MTND4P23
MTND4P24
MTND4P26
MTND4P35
MTND4P6
MTND5P10
MTND5P11
MTND5P12
MTND5P14
MTND5P2
MTND5P24
MTND5P28
MTND5P32
MTND6P22
MTND6P3

MTND6P4
MTO1
MTOR
MTPAP
MTPN
MTR
MTRES1
MTREX
MTRF1
MTRF1L
MTRNR2L1
MTRNR2L10
MTRNR2L11
MTRNR2L12
MTRNR2L13
MTRNR2L3
MTRNR2L4
MTRNR2L5
MTRNR2L6
MTRNR2L7
MTRNR2L8
MTRR
MTSS1
MTSS2
MTURN
MTUS1
MTUS2
MTX1
MTX2
MTX3
MUC1
MUC12
MUC13
MUC15
MUC16
MUC17
MUC19
MUC2
MUC20
MUC20P1
MUC21
MUC22
MUC3A
MUC4
MUC5AC
MUC5B
MUC6
MUC7
MUCL1
MUCL3
MUL1
MUS81
MUTYH
MVB12A
MVB12B
MVD
MVK
MVP

MX1
MX2
MXD1
MXD3
MXD4
MXI1
MXRA5
MXRA7
MYADM
MYB
MYBBP1A
MYBL1
MYBL2
MYBPC1
MYBPC3
MYC
MYCBP
MYCBP2
MYCBPAP
MYCL
MYCT1
MYD88
MYDGF
MYEF2
MYEOV
MYH10
MYH11
MYH14
MYH15
MYH16
MYH3
MYH7B
MYH9
MYL12A
MYL12B
MYL5
MYL6
MYL6B
MYL9
MYLIP
MYLK
MYLK3
MYNN
MYO10
MYO15A
MYO15B
MYO16
MYO18A
MYO19
MYO1B
MYO1C
MYO1D
MYO1E
MYO1F
MYO1G
MYO1H
MYO3B
MYO5A

MYO5B
MYO5C
MYO6
MYO7A
MYO7B
MYO9A
MYO9B
MYOCOS
MYOF
MYOM1
MYOM2
MYOM3
MYORG
MYOT
MYOZ3
MYPOP
MYRF
MYRFL
MYRIP
MYSM1
MYT1L
MYZAP
MZB1
MZF1
MZT1
MZT2A
MZT2B
N4BP1
N4BP2
N4BP2L1
N4BP2L2
N4BP3
N6AMT1
NAA10
NAA15
NAA16
NAA20
NAA25
NAA30
NAA35
NAA38
NAA40
NAA50
NAA60
NAA80
NAAA
NAALADL2
NAB1
NAB2
NABP1
NABP2
NACA
NACA4P
NACC1
NACC2
NADK
NADK2
NADSYN1

NAE1
NAF1
NAGA
NAGK
NAGLU
NAGPA
NAIF1
NAIP
NAIPP1
NAIPP2
NAIPP3
NAIPP4
NALCN
NAMPT
NAMPTP1
NANOG
NANOGP1
NANOS1
NANP
NANS
NAP1L1
NAP1L4
NAP1L4P1
NAP1L5
NAPA
NAPB
NAPEPLD
NAPG
NAPRT
NAPSB
NARF
NARS1
NARS2
NASP
NAT1
NAT10
NAT14
NAT8
NAT9
NATD1
NAV1
NAV2
NAV3
NAXD
NAXE
NBAS
NBDY
NBEA
NBEAL1
NBEAL2
NBEAP1
NBL1
NBN
NBPF1
NBPF10
NBPF11
NBPF12
NBPF14

NBPF15
NBPF19
NBPF20
NBPF25P
NBPF26
NBPF2P
NBPF3
NBPF8
NBPF9
NBR1
NCALD
NCAM1
NCAM2
NCAPD2
NCAPD3
NCAPG
NCAPG2
NCAPH
NCAPH2
NCBP1
NCBP2
NCBP2AS2
NCBP3
NCCRP1
NCDN
NCEH1
NCF1
NCF1B
NCF1C
NCF2
NCF4
NCK1
NCK2
NCKAP1
NCKAP1L
NCKAP5
NCKAP5L
NCKIPSD
NCL
NCLN
NCLP1
NCMAP
NCOA1
NCOA2
NCOA3
NCOA4
NCOA5
NCOA6
NCOA7
NCOR1
NCOR2
NCR1
NCR3LG1
NCS1
NCSTN
NDC1
NDC80
NDE1

NDEL1
NDFIP1
NDFIP2
NDOR1
NDRG1
NDRG2
NDRG3
NDRG4
NDST1
NDST2
NDST3
NDUFA1
NDUFA10
NDUFA11
NDUFA12
NDUFA13
NDUFA2
NDUFA3
NDUFA4
NDUFA5
NDUFA6
NDUFA7
NDUFA8
NDUFA9
NDUFAB1
NDUFAF1
NDUFAF2
NDUFAF3
NDUFAF4
NDUFAF5
NDUFAF6
NDUFAF7
NDUFAF8
NDUFB1
NDUFB10
NDUFB11
NDUFB2
NDUFB3
NDUFB4
NDUFB4P11
NDUFB5
NDUFB6
NDUFB7
NDUFB8
NDUFB9
NDUFC1
NDUFC2
NDUFC2-KCTD14
NDUFS1
NDUFS2
NDUFS3
NDUFS4
NDUFS5
NDUFS6
NDUFS7
NDUFS8
NDUFV1
NDUFV2

NDUFV2P1
NDUFV3
NEB
NEBL
NECAB1
NECAB3
NECAP1
NECAP2
NECTIN1
NECTIN2
NECTIN3
NECTIN4
NEDD1
NEDD4
NEDD4L
NEDD8
NEDD9
NEGR1
NEIL1
NEIL2
NEIL3
NEK1
NEK10
NEK11
NEK2
NEK3
NEK4
NEK5
NEK6
NEK7
NEK8
NEK9
NELFA
NELFB
NELFCD
NELFE
NELL2
NEMF
NEMP1
NEMP2
NENF
NEO1
NEPRO
NET1
NETO2
NEU1
NEU3
NEURL1
NEURL1B
NEURL3
NEURL4
NEXMIF
NEXN
NF1
NF2
NFAM1
NFASC
NFAT5

NFATC1
NFATC2
NFATC2IP
NFATC3
NFE2
NFE2L1
NFE2L2
NFE2L3
NFE4
NFIA
NFIB
NFIC
NFIL3
NFIX
NFKB1
NFKB2
NFKBIA
NFKBIB
NFKBID
NFKBIE
NFKBIL1
NFKBIZ
NFRKB
NFS1
NFU1
NFX1
NFXL1
NFYA
NFYB
NFYC
NGDN
NGEF
NGLY1
NGRN
NHEJ1
NHLRC1
NHLRC2
NHLRC3
NHLRC4
NHP2
NHS
NHSL1
NHSL2
NIBAN1
NIBAN2
NIBAN3
NICN1
NID1
NID2
NIF3L1
NIFK
NIM1K
NIN
NINJ1
NINJ2
NINL
NIP7
NIPA1

NIPA2
NIPAL1
NIPAL2
NIPAL3
NIPAL4
NIPBL
NIPSNAP1
NIPSNAP2
NIPSNAP3A
NIPSNAP3B
NISCH
NIT1
NIT2
NKAP
NKAPD1
NKAPL
NKAPP1
NKD1
NKG7
NKIRAS1
NKIRAS2
NKRF
NKTR
NKX3-1
NLE1
NLGN3
NLGN4X
NLGN4Y
NLK
NLN
NLRC3
NLRC4
NLRC5
NLRP1
NLRP10
NLRP12
NLRP14
NLRP2
NLRP3
NLRP6
NLRP7
NLRP9P1
NLRX1
NMB
NMD3
NME1
NME2
NME3
NME4
NME5
NME6
NME7
NME9
NMI
NMNAT1
NMNAT2
NMNAT3
NMRAL1

NMRK1
NMT1
NMT2
NMUR1
NNT
NOA1
NOB1
NOC2L
NOC3L
NOC4L
NOCT
NOD1
NOD2
NOL10
NOL11
NOL12
NOL3
NOL4L
NOL6
NOL7
NOL8
NOL9
NOLC1
NOM1
NOMO1
NOMO2
NOMO3
NONO
NONOP2
NOP10
NOP14
NOP16
NOP2
NOP53
NOP56
NOP58
NOP9
NOS1
NOS1AP
NOS2
NOS2P1
NOS2P3
NOS3
NOSIP
NOSTRIN
NOTCH1
NOTCH2
NOTCH2NLA
NOTCH3
NOTCH4
NOVA1
NOX4
NOX5
NOXA1
NOXRED1
NPAP1
NPAS2
NPAS3

NPAT
NPB
NPBWR1
NPC1
NPC2
NPDC1
NPEPL1
NPEPPS
NPFFR1
NPHP1
NPHP3
NPHP3-ACAD11
NPHP4
NPHS1
NPIPA1
NPIPA3
NPIPA5
NPIPA7
NPIPA8
NPIPA9
NPIPB11
NPIPB12
NPIPB13
NPIPB14P
NPIPB15
NPIPB2
NPIPB3
NPIPB4
NPIPB5
NPIPB6
NPIPB9
NPIPP1
NPL
NPLOC4
NPM1
NPM1P13
NPM1P14
NPM1P25
NPM1P26
NPM1P27
NPM1P29
NPM1P35
NPM1P39
NPM2
NPM3
NPNT
NPR2
NPR3
NPRL2
NPRL3
NPTN
NPTX1
NPTXR
NPY6R
NQO1
NQO2
NR1D1
NR1D2

NR1H2
NR1H3
NR1I2
NR1I3
NR2C1
NR2C2
NR2C2AP
NR2E1
NR2F2
NR2F6
NR3C1
NR3C2
NR4A1
NR4A2
NR4A3
NR5A2
NR6A1
NRARP
NRAS
NRBF2
NRBP1
NRBP2
NRCAM
NRDC
NRDE2
NREP
NRF1
NRG2
NRG4
NRIP1
NRIP2
NRIP3
NRM
NRP1
NRP2
NRROS
NRSN1
NRSN2
NRXN1
NRXN3
NSA2
NSD1
NSD2
NSD3
NSDHL
NSF
NSFL1C
NSFP1
NSG1
NSL1
NSMAF
NSMCE1
NSMCE2
NSMCE3
NSMCE4A
NSMF
NSRP1
NSRP1P1

NSUN2
NSUN3
NSUN4
NSUN5
NSUN5P1
NSUN5P2
NSUN6
NSUN7
NT5C
NT5C1B
NT5C2
NT5C3A
NT5C3AP1
NT5C3B
NT5DC1
NT5DC2
NT5DC3
NT5E
NTAN1
NTMT1
NTN1
NTN4
NTNG2
NTPCR
NTRK2
NTRK3
NTS
NUAK1
NUAK2
NUB1
NUBP1
NUBP2
NUBPL
NUCB1
NUCB2
NUCKS1
NUDC
NUDCD1
NUDCD2
NUDCD3
NUDT1
NUDT12
NUDT13
NUDT14
NUDT15
NUDT16
NUDT16L1
NUDT16P1
NUDT17
NUDT18
NUDT19
NUDT2
NUDT21
NUDT22
NUDT3
NUDT4
NUDT4B
NUDT4P2

NUDT5
NUDT6
NUDT7
NUDT8
NUDT9
NUFIP1
NUFIP2
NUGGC
NUMA1
NUMB
NUMBL
NUP107
NUP133
NUP153
NUP155
NUP160
NUP188
NUP205
NUP210
NUP210L
NUP214
NUP35
NUP37
NUP42
NUP43
NUP50
NUP54
NUP58
NUP62
NUP62CL
NUP85
NUP88
NUP93
NUP98
NUPR1
NUS1
NUS1P1
NUSAP1
NUTF2
NUTM2A
NUTM2B
NUTM2D
NUTM2G
NVL
NWD1
NXF1
NXF2
NXF2B
NXF3
NXN
NXNL2
NXPE3
NXT1
NXT2
NYNRIN
OAF
OARD1
OAS1

OAS2
OAS3
OASL
OAT
OAZ1
OAZ2
OAZ3
OBI1
OBSCN
OBSL1
OCEL1
OCIAD1
OCIAD2
OCLN
OCRL
ODAM
ODAPH
ODC1
ODCP
ODF2
ODF2L
ODF3B
ODR4
OFD1
OFD1P17
OGA
OGDH
OGFOD1
OGFOD2
OGFOD3
OGFR
OGFRL1
OGG1
OGN
OGT
OLA1
OLA1P2
OLA1P3
OLAH
OLFM4
OLFM5P
OLFML1
OLFML2A
OLFML2B
OLFML3
OLR1
OMA1
OMG
ONECUT1
ONECUT2
ONECUT3
OPA1
OPA3
OPHN1
OPLAH
OPN3
OPRD1
OPRL1

OPRM1
OPTN
OR11A1
OR13K1P
OR1F2P
OR2A4
OR2A9P
OR2C3
OR4F15
OR51B5
OR52A1
OR52B6
OR52I1
OR52K1
OR52K3P
OR5A2
OR5BA1P
OR6J1
OR7A5
OR7D2
OR7E104P
OR7E122P
OR7E14P
OR7E22P
OR7E36P
OR7E38P
OR7E47P
OR7E94P
ORAI1
ORAI2
ORAI3
ORC1
ORC2
ORC3
ORC4
ORC5
ORC6
ORMDL1
ORMDL2
ORMDL3
OS9
OSBP
OSBP2
OSBPL10
OSBPL11
OSBPL1A
OSBPL2
OSBPL3
OSBPL5
OSBPL6
OSBPL7
OSBPL8
OSBPL9
OSCAR
OSCP1
OSER1
OSGEP
OSGEPL1

OSGIN1
OSGIN2
OSM
OSMR
OSR1
OST4
OSTC
OSTF1
OSTM1
OTOA
OTOAP1
OTOF
OTUB1
OTUB2
OTUD1
OTUD3
OTUD4
OTUD4P1
OTUD5
OTUD6A
OTUD6B
OTUD7A
OTUD7B
OTULIN
OTULINL
OTX1
OTX2
OVCA2
OVGP1
OVOL1
OVOL2
OXA1L
OXCT1
OXLD1
OXNAD1
OXR1
OXSM
OXSR1
OXTR
P2RX1
P2RX4
P2RX5
P2RX5-TAX1BP3
P2RX6
P2RX7
P2RY1
P2RY10
P2RY11
P2RY12
P2RY13
P2RY14
P2RY2
P2RY6
P2RY8
P3H1
P3H2
P3H3
P3H4

P3R3URF-PIK3R3
P4HA1
P4HA2
P4HA3
P4HB
P4HTM
PA2G4
PA2G4P4
PA2G4P6
PAAF1
PABPC1
PABPC1L
PABPC1P4
PABPC4
PABPN1
PACC1
PACRG
PACRGL
PACS1
PACS2
PACSIN1
PACSIN2
PACSIN3
PADI1
PADI2
PADI3
PADI4
PAF1
PAFAH1B1
PAFAH1B2
PAFAH1B3
PAFAH2
PAG1
PAGR1
PAICS
PAIP1
PAIP2
PAIP2B
PAK1
PAK1IP1
PAK2
PAK3
PAK4
PAK6
PALB2
PALD1
PALLD
PALM
PALM2AKAP2
PALMD
PAM
PAM16
PAMR1
PAN2
PAN3
PANK1
PANK2
PANK3

PANK4
PANO1
PANX1
PANX2
PAOX
PAPLN
PAPOLA
PAPOLG
PAPPA
PAPPA2
PAPSS1
PAPSS2
PAQR3
PAQR4
PAQR5
PAQR7
PAQR8
PARD3
PARD3B
PARD6A
PARD6B
PARD6G
PARG
PARGP1
PARK7
PARL
PARM1
PARN
PARP1
PARP10
PARP11
PARP12
PARP14
PARP15
PARP16
PARP2
PARP3
PARP4
PARP4P2
PARP6
PARP8
PARP9
PARPBP
PARS2
PARVA
PARVB
PARVG
PASK
PATE2
PATE4
PATJ
PATL1
PATL2
PATZ1
PAWR
PAX3
PAX5
PAX6

PAX7
PAX8
PAX9
PAXBP1
PAXIP1
PAXX
PBDC1
PBLD
PBOV1
PBRM1
PBX1
PBX2
PBX3
PBX4
PBXIP1
PC
PCARE
PCBD1
PCBD2
PCBP1
PCBP2
PCBP3
PCBP4
PCCA
PCCB
PCDH1
PCDH10
PCDH11X
PCDH11Y
PCDH12
PCDH15
PCDH17
PCDH19
PCDH20
PCDH7
PCDH9
PCDHA10
PCDHA4
PCDHA9
PCDHAC1
PCDHAC2
PCDHB1
PCDHB11
PCDHB13
PCDHB14
PCDHB16
PCDHB2
PCDHB9
PCDHGA1
PCDHGA10
PCDHGA11
PCDHGA6
PCDHGA8
PCDHGA9
PCDHGB4
PCDHGB6
PCDHGB7
PCDHGB8P

PCDHGC3
PCDHGC4
PCED1A
PCED1B
PCF11
PCGF1
PCGF2
PCGF3
PCGF5
PCGF6
PCID2
PCIF1
PCK1
PCK2
PCLAF
PCLO
PCM1
PCMT1
PCMTD1
PCMTD2
PCNA
PCNP
PCNT
PCNX1
PCNX2
PCNX3
PCNX4
PCOLCE
PCP4L1
PCSK1
PCSK1N
PCSK2
PCSK4
PCSK5
PCSK6
PCSK7
PCTP
PCYOX1
PCYOX1L
PCYT1A
PCYT1B
PCYT2
PDAP1
PDCD1
PDCD10
PDCD11
PDCD1LG2
PDCD2
PDCD2L
PDCD4
PDCD5
PDCD6
PDCD6IP
PDCD6IPP1
PDCD6IPP2
PDCD7
PDCL
PDCL3

PDCL3P4
PDE10A
PDE11A
PDE12
PDE1A
PDE1B
PDE1C
PDE2A
PDE3A
PDE3B
PDE4A
PDE4B
PDE4C
PDE4D
PDE4DIP
PDE5A
PDE6A
PDE6B
PDE6D
PDE7A
PDE7B
PDE8A
PDE8B
PDE9A
PDF
PDGFA
PDGFC
PDGFD
PDGFRA
PDGFRL
PDHA1
PDHB
PDHX
PDIA3
PDIA3P1
PDIA4
PDIA5
PDIA6
PDIK1L
PDK1
PDK2
PDK3
PDK4
PDLIM1
PDLIM2
PDLIM3
PDLIM4
PDLIM5
PDLIM7
PDP1
PDP2
PDPK1
PDPK2P
PDPN
PDPR
PDRG1
PDS5A
PDS5B

PDSS1
PDSS1P1
PDSS2
PDXDC1
PDXDC2P
PDXK
PDXP
PDZD11
PDZD2
PDZD7
PDZD8
PDZD9
PDZK1
PDZK1IP1
PEA15
PEAK1
PEAK3
PEBP1
PECAM1
PECR
PEF1
PEG10
PELI1
PELI2
PELI3
PELO
PELP1
PEMT
PEPD
PER1
PER2
PER3
PERM1
PERP
PES1
PET100
PET117
PEX1
PEX10
PEX11A
PEX11B
PEX11G
PEX12
PEX13
PEX14
PEX16
PEX19
PEX2
PEX26
PEX3
PEX5
PEX5L
PEX6
PEX7
PFAS
PFDN1
PFDN2
PFDN4

PFDN5
PFDN6
PFKFB2
PFKFB3
PFKFB4
PFKL
PFKM
PFKP
PFN1
PFN1P2
PFN2
PGA4
PGAM1
PGAM1P5
PGAM1P8
PGAM5
PGAP1
PGAP2
PGAP3
PGAP4
PGAP6
PGBD1
PGBD2
PGBD4
PGBD5
PGD
PGF
PGGHG
PGGT1B
PGK1
PGLS
PGM1
PGM2
PGM2L1
PGM3
PGM5
PGM5P2
PGP
PGPEP1
PGR
PGRMC1
PGRMC2
PGS1
PHACTR1
PHACTR2
PHACTR3
PHACTR4
PHAX
PHB
PHB2
PHBP12
PHC1
PHC1P1
PHC2
PHC3
PHETA1
PHETA2
PHEX

PHF1
PHF10
PHF11
PHF12
PHF13
PHF14
PHF19
PHF2
PHF20
PHF20L1
PHF21A
PHF23
PHF24
PHF3
PHF5A
PHF6
PHF7
PHF8
PHGDH
PHIP
PHKA1
PHKA1P1
PHKA2
PHKB
PHKG1
PHKG2
PHLDA1
PHLDA2
PHLDA3
PHLDB1
PHLDB2
PHLDB3
PHLPP1
PHLPP2
PHOSPHO1
PHOSPHO2
PHPT1
PHRF1
PHTF1
PHTF2
PHYH
PHYHD1
PHYKPL
PI3
PI4K2A
PI4K2B
PI4KA
PI4KAP1
PI4KAP2
PI4KB
PIAS1
PIAS2
PIAS3
PIAS4
PIBF1
PICALM
PICK1
PID1

PIDD1
PIEZO1
PIEZO2
PIF1
PIFO
PIGA
PIGB
PIGBOS1
PIGC
PIGCP1
PIGF
PIGG
PIGH
PIGK
PIGL
PIGM
PIGN
PIGO
PIGP
PIGQ
PIGR
PIGS
PIGT
PIGU
PIGV
PIGW
PIGX
PIGZ
PIH1D1
PIH1D2
PIH1D3
PIK3AP1
PIK3C2A
PIK3C2B
PIK3C2G
PIK3C3
PIK3CA
PIK3CB
PIK3CD
PIK3CG
PIK3IP1
PIK3R1
PIK3R2
PIK3R3
PIK3R4
PIK3R5
PIK3R6
PIKFYVE
PILRA
PILRB
PIM1
PIM2
PIM3
PIN1
PIN4
PINK1
PINLYP
PINX1

PIP
PIP4K2A
PIP4K2B
PIP4K2C
PIP4P1
PIP4P2
PIP5K1A
PIP5K1B
PIP5K1C
PIP5KL1
PIPOX
PIPSL
PIR
PISD
PITHD1
PITPNA
PITPNB
PITPNC1
PITPNM1
PITPNM2
PITPNM3
PITRM1
PITX1
PIWIL2
PIWIL4
PJA1
PJA2
PKD1
PKD1L1
PKD1L2
PKD1L3
PKD1P1
PKD1P5
PKD1P6
PKD2
PKD2L2
PKDREJ
PKHD1
PKHD1L1
PKIA
PKIB
PKIG
PKM
PKMYT1
PKN1
PKN2
PKN3
PKNOX1
PKNOX2
PKP1
PKP2
PKP3
PKP4
PLA1A
PLA2G10
PLA2G12A
PLA2G15
PLA2G2C

PLA2G4A
PLA2G4B
PLA2G4C
PLA2G4D
PLA2G4E
PLA2G4F
PLA2G6
PLA2G7
PLA2R1
PLAA
PLAAT2
PLAAT3
PLAAT4
PLAAT5
PLAC8
PLAG1
PLAGL1
PLAGL2
PLAT
PLAU
PLAUR
PLB1
PLBD1
PLBD2
PLCB1
PLCB2
PLCB3
PLCB4
PLCD1
PLCD3
PLCD4
PLCE1
PLCG1
PLCG2
PLCH1
PLCH2
PLCL1
PLCL2
PLCXD1
PLCXD2
PLCXD3
PLD1
PLD2
PLD3
PLD4
PLD6
PLEC
PLEK
PLEK2
PLEKHA1
PLEKHA2
PLEKHA3
PLEKHA4
PLEKHA5
PLEKHA6
PLEKHA7
PLEKHA8
PLEKHA8P1

PLEKHB1
PLEKHB2
PLEKHD1
PLEKHF1
PLEKHF2
PLEKHG1
PLEKHG2
PLEKHG3
PLEKHG4
PLEKHG4B
PLEKHG5
PLEKHG6
PLEKHG7
PLEKHH1
PLEKHH2
PLEKHH3
PLEKHJ1
PLEKHM1
PLEKHM1P1
PLEKHM2
PLEKHM3
PLEKHN1
PLEKHO1
PLEKHO2
PLEKHS1
PLG
PLGLB1
PLGLB2
PLGRKT
PLIN2
PLIN3
PLIN4
PLIN5
PLK1
PLK2
PLK3
PLK4
PLK5
PLLP
PLN
PLOD1
PLOD2
PLOD3
PLP2
PLPBP
PLPP1
PLPP2
PLPP3
PLPP5
PLPP6
PLPPR2
PLPPR3
PLRG1
PLS1
PLS3
PLSCR1
PLSCR2
PLSCR3

PLSCR4
PLTP
PLVAP
PLXDC1
PLXDC2
PLXNA1
PLXNA2
PLXNA3
PLXNA4
PLXNB1
PLXNB2
PLXNB3
PLXNC1
PLXND1
PM20D2
PMAIP1
PMEL
PMEPA1
PMF1
PMFBP1
PML
PMM1
PMM2
PMP2
PMP22
PMPCA
PMPCB
PMS1
PMS2
PMS2CL
PMS2P1
PMS2P10
PMS2P2
PMS2P3
PMS2P6
PMS2P7
PMS2P9
PMVK
PNISR
PNKD
PNKP
PNLDC1
PNLIPRP1
PNMA1
PNMA2
PNMA8A
PNMA8C
PNN
PNO1
PNP
PNPLA1
PNPLA2
PNPLA3
PNPLA4
PNPLA6
PNPLA7
PNPLA8
PNPO

PNPT1
PNRC1
PNRC2
POC1A
POC1B
POC1B-GALNT4
POC5
PODNL1
PODXL
PODXL2
POF1B
POFUT1
POFUT2
POGK
POGLUT1
POGLUT3
POGZ
POLA1
POLA2
POLB
POLD1
POLD2
POLD3
POLD4
POLDIP2
POLDIP3
POLE
POLE2
POLE3
POLE4
POLG
POLG2
POLH
POLI
POLK
POLL
POLM
POLN
POLQ
POLR1A
POLR1B
POLR1C
POLR1D
POLR1E
POLR2A
POLR2B
POLR2C
POLR2D
POLR2E
POLR2F
POLR2G
POLR2H
POLR2I
POLR2J
POLR2J2
POLR2J3
POLR2J4
POLR2K

POLR2L
POLR2M
POLR3A
POLR3B
POLR3C
POLR3D
POLR3E
POLR3F
POLR3G
POLR3GL
POLR3H
POLR3K
POLRMT
POM121
POM121B
POM121C
POM121L9P
POMGNT1
POMGNT2
POMK
POMP
POMT1
POMT2
POMZP3
PON2
PON3
POP1
POP4
POP5
POP7
POPDC2
POR
PORCN
POSTN
POT1
POTEC
POTEH
POTEI
POU2AF1
POU2F1
POU2F2
POU2F3
POU5F1
POU5F1B
POU5F2
POU6F1
POU6F2
PP2D1
PPA1
PPA2
PPAN
PPAN-P2RY11
PPARA
PPARD
PPARG
PPARGC1A
PPARGC1B
PPAT

PPCDC
PPCS
PPDPF
PPEF2
PPFIA1
PPFIBP1
PPFIBP2
PPHLN1
PPIA
PPIAL4G
PPIAP22
PPIAP46
PPIAP51
PPIB
PPIC
PPID
PPIE
PPIF
PPIG
PPIH
PPIL1
PPIL2
PPIL3
PPIL4
PPIL6
PPIP5K1
PPIP5K2
PPL
PPM1A
PPM1B
PPM1D
PPM1E
PPM1F
PPM1G
PPM1H
PPM1J
PPM1K
PPM1L
PPM1M
PPM1N
PPME1
PPOX
PPP1CA
PPP1CB
PPP1CC
PPP1R10
PPP1R11
PPP1R12A
PPP1R12B
PPP1R12C
PPP1R13B
PPP1R13L
PPP1R14B
PPP1R14C
PPP1R15A
PPP1R15B
PPP1R16A
PPP1R16B

PPP1R18
PPP1R1B
PPP1R1C
PPP1R2
PPP1R21
PPP1R26
PPP1R32
PPP1R35
PPP1R36
PPP1R37
PPP1R3B
PPP1R3C
PPP1R3D
PPP1R3E
PPP1R3F
PPP1R3G
PPP1R42
PPP1R7
PPP1R8
PPP1R9A
PPP1R9B
PPP2CA
PPP2CB
PPP2R1A
PPP2R1B
PPP2R2A
PPP2R2B
PPP2R2C
PPP2R2D
PPP2R3A
PPP2R3B
PPP2R3C
PPP2R5A
PPP2R5B
PPP2R5C
PPP2R5D
PPP2R5E
PPP3CA
PPP3CB
PPP3CC
PPP3R1
PPP4C
PPP4R1
PPP4R1L
PPP4R2
PPP4R3A
PPP4R3B
PPP4R4
PPP5C
PPP5D1
PPP6C
PPP6R1
PPP6R2
PPP6R3
PPRC1
PPT1
PPT2
PPTC7

PPWD1
PQBP1
PRADC1
PRAF2
PRAG1
PRAM1
PRB3
PRB4
PRC1
PRCC
PRCP
PRDM1
PRDM10
PRDM11
PRDM15
PRDM2
PRDM4
PRDM5
PRDM6
PRDM7
PRDM8
PRDX1
PRDX2
PRDX3
PRDX3P1
PRDX4
PRDX5
PRDX6
PREB
PRELID1
PRELID1P1
PRELID2
PRELID3B
PRELP
PREP
PREPL
PREX1
PREX2
PRF1
PRICKLE1
PRICKLE2
PRICKLE3
PRIM1
PRIM2
PRIMPOL
PRKAA1
PRKAA2
PRKAB1
PRKAB2
PRKACA
PRKACB
PRKAG1
PRKAG2
PRKAR1A
PRKAR1B
PRKAR2A
PRKAR2B
PRKCA

PRKCB
PRKCD
PRKCE
PRKCH
PRKCI
PRKCQ
PRKCSH
PRKCZ
PRKD2
PRKD3
PRKDC
PRKG2
PRKN
PRKRA
PRKRIP1
PRKX
PRKXP1
PRKY
PRLHR
PRLR
PRMT1
PRMT2
PRMT3
PRMT5
PRMT6
PRMT7
PRMT8
PRMT9
PRND
PRNP
PROB1
PROC
PROCA1
PROCR
PRODH
PROK2
PROM1
PROM2
PRORP
PRORSD1P
PROS1
PROSER1
PROSER2
PROSER3
PROX1
PROX2
PRPF18
PRPF19
PRPF3
PRPF31
PRPF38A
PRPF38B
PRPF39
PRPF4
PRPF40A
PRPF40B
PRPF4B
PRPF6

PRPF8
PRPH
PRPS1
PRPS1P2
PRPS2
PRPSAP1
PRPSAP2
PRR11
PRR12
PRR13
PRR14
PRR14L
PRR15
PRR15L
PRR18
PRR19
PRR26
PRR27
PRR29
PRR3
PRR4
PRR5
PRR5L
PRR7
PRRC1
PRRC2A
PRRC2B
PRRC2C
PRRG1
PRRG2
PRRG3
PRRG4
PRRT2
PRRT3
PRRX1
PRSS12
PRSS16
PRSS22
PRSS23
PRSS27
PRSS53
PRSS54
PRSS8
PRTG
PRUNE1
PRUNE2
PRX
PRXL2A
PRXL2B
PRXL2C
PSAP
PSAT1
PSCA
PSD
PSD3
PSD4
PSEN1
PSEN2

PSENEN
PSG2
PSIP1
PSKH1
PSMA1
PSMA2
PSMA3
PSMA4
PSMA5
PSMA6
PSMA7
PSMB1
PSMB10
PSMB2
PSMB3
PSMB4
PSMB5
PSMB6
PSMB7
PSMB8
PSMB9
PSMC1
PSMC1P1
PSMC2
PSMC3
PSMC3IP
PSMC4
PSMC5
PSMC6
PSMD1
PSMD10
PSMD10P1
PSMD11
PSMD12
PSMD13
PSMD14
PSMD2
PSMD3
PSMD4
PSMD5
PSMD6
PSMD7
PSMD8
PSMD9
PSME1
PSME2
PSME3
PSME3IP1
PSME4
PSMF1
PSMG1
PSMG2
PSMG3
PSMG4
PSPC1
PSPH
PSPN
PSTK

PSTPIP1
PSTPIP2
PTAFR
PTAR1
PTBP1
PTBP2
PTBP3
PTCD1
PTCD2
PTCD3
PTCH1
PTCH2
PTCHD1
PTCHD4
PTDSS1
PTDSS2
PTEN
PTENP1
PTER
PTGDR
PTGDS
PTGER2
PTGER3
PTGER4
PTGES
PTGES2
PTGES3
PTGES3L
PTGFR
PTGFRN
PTGIR
PTGIS
PTGR1
PTGR2
PTGS1
PTGS2
PTK2
PTK2B
PTK6
PTK7
PTMA
PTMS
PTN
PTOV1
PTP4A1
PTP4A2
PTP4A3
PTPA
PTPDC1
PTPMT1
PTPN1
PTPN11
PTPN12
PTPN13
PTPN14
PTPN18
PTPN2
PTPN20

PTPN21
PTPN22
PTPN23
PTPN3
PTPN4
PTPN6
PTPN7
PTPN9
PTPRA
PTPRB
PTPRC
PTPRCAP
PTPRD
PTPRE
PTPRF
PTPRG
PTPRH
PTPRJ
PTPRK
PTPRM
PTPRN2
PTPRO
PTPRQ
PTPRS
PTPRT
PTPRU
PTPRVP
PTPRZ1
PTRH1
PTRH2
PTRHD1
PTS
PTTG1
PTTG1IP
PTX3
PUDP
PUF60
PUM1
PUM2
PUM3
PURA
PURB
PUS1
PUS10
PUS3
PUS7
PUS7L
PUSL1
PVR
PVRIG
PVRIG2P
PWP1
PWP2
PWWP2A
PWWP2B
PWWP3A
PXDC1
PXDN

PXK
PXMP2
PXMP4
PXN
PXYLP1
PYCARD
PYCR1
PYCR2
PYCR3
PYGB
PYGL
PYGM
PYGO1
PYGO2
PYHIN1
PYM1
PYROXD1
PYROXD2
PYURF
PZP
QARS1
QDPR
QKI
QPCT
QPCTL
QPRT
QRICH1
QRICH2
QRSL1
QRSL1P3
QSER1
QSOX1
QSOX2
QTRT1
QTRT2
R3HCC1
R3HCC1L
R3HDM1
R3HDM2
R3HDM4
RAB10
RAB11A
RAB11B
RAB11FIP1
RAB11FIP1P1
RAB11FIP2
RAB11FIP3
RAB11FIP4
RAB11FIP5
RAB12
RAB13
RAB14
RAB15
RAB17
RAB18
RAB19
RAB1A
RAB1B

RAB20
RAB21
RAB22A
RAB23
RAB24
RAB25
RAB26
RAB27A
RAB27B
RAB28
RAB29
RAB2A
RAB2B
RAB30
RAB31
RAB32
RAB33B
RAB34
RAB35
RAB36
RAB37
RAB38
RAB39A
RAB39B
RAB3B
RAB3D
RAB3GAP1
RAB3GAP2
RAB3IP
RAB40A
RAB40B
RAB40C
RAB42
RAB43
RAB44
RAB4A
RAB4B
RAB4B-EGLN2
RAB5A
RAB5B
RAB5C
RAB5IF
RAB6A
RAB6B
RAB7A
RAB8A
RAB8B
RAB9A
RABAC1
RABEP1
RABEP2
RABEPK
RABGAP1
RABGAP1L
RABGEF1
RABGGTA
RABGGTB
RABIF

RABL2A
RABL2B
RABL3
RABL6
RAC1
RAC1P2
RAC2
RACGAP1
RACK1
RAD1
RAD17
RAD17P2
RAD18
RAD21
RAD23A
RAD23B
RAD50
RAD51
RAD51AP1
RAD51B
RAD51C
RAD51D
RAD52
RAD54B
RAD54L
RAD54L2
RAD9A
RAD9B
RADIL
RADX
RAE1
RAET1E
RAET1G
RAF1
RAG1
RAI1
RAI14
RAI2
RALA
RALB
RALBP1
RALGAPA1
RALGAPA1P1
RALGAPA2
RALGAPB
RALGDS
RALGPS1
RALGPS2
RALY
RAMAC
RAMP1
RAN
RANBP1
RANBP10
RANBP17
RANBP2
RANBP3
RANBP6

RANBP9
RANGAP1
RANGRF
RAP1A
RAP1B
RAP1GAP
RAP1GAP2
RAP1GDS1
RAP2A
RAP2B
RAP2C
RAPGEF1
RAPGEF2
RAPGEF3
RAPGEF4
RAPGEF5
RAPGEF6
RAPGEFL1
RAPH1
RARA
RARB
RARG
RARRES1
RARRES2
RARS1
RARS2
RASA1
RASA2
RASA3
RASA4
RASA4B
RASA4CP
RASAL1
RASAL2
RASAL3
RASD1
RASEF
RASGEF1A
RASGEF1B
RASGRF1
RASGRF2
RASGRP1
RASGRP2
RASGRP3
RASGRP4
RASIP1
RASL10B
RASL11A
RASSF1
RASSF10
RASSF2
RASSF3
RASSF4
RASSF5
RASSF6
RASSF7
RASSF8
RASSF9

RAVER1
RAVER2
RB1
RB1CC1
RBAK
RBBP4
RBBP5
RBBP6
RBBP7
RBBP8
RBBP8NL
RBBP9
RBCK1
RBFA
RBFOX2
RBFOX3
RBIS
RBKS
RBL1
RBL2
RBM10
RBM11
RBM12
RBM12B
RBM14
RBM14-RBM4
RBM15
RBM15B
RBM17
RBM18
RBM19
RBM20
RBM22
RBM22P2
RBM23
RBM24
RBM25
RBM26
RBM27
RBM28
RBM3
RBM33
RBM34
RBM38
RBM39
RBM4
RBM41
RBM42
RBM43
RBM44
RBM45
RBM47
RBM48
RBM4B
RBM5
RBM6
RBM7
RBM8A

RBMS1
RBMS1P1
RBMS2
RBMS2P1
RBMS3
RBMX
RBMX2
RBMXL1
RBP1
RBPJ
RBPMS
RBSN
RBX1
RC3H1
RC3H2
RCAN1
RCAN2
RCAN3
RCBTB1
RCBTB2
RCC1
RCC1L
RCC2
RCCD1
RCE1
RCHY1
RCL1
RCN1
RCN1P2
RCN2
RCOR1
RCOR3
RCSD1
RCVRN
RD3
RDH10
RDH11
RDH13
RDH14
RDH16
RDH5
RDM1P5
RDX
REC8
RECK
RECQL
RECQL4
RECQL5
REEP1
REEP2
REEP3
REEP4
REEP5
REEP6
REL
RELA
RELB
RELCH

RELL1
RELL2
RELN
RELT
REM1
REM2
RENBP
REPIN1
REPS1
REPS2
RER1
RERE
RERG
RESF1
REST
RET
RETREG1
RETREG2
RETREG3
RETSAT
REV1
REV3L
REX1BD
REXO1
REXO1L1P
REXO2
REXO4
REXO5
RFC1
RFC2
RFC3
RFC4
RFC5
RFFL
RFK
RFLNB
RFNG
RFT1
RFTN1
RFTN2
RFWD3
RFX1
RFX2
RFX3
RFX5
RFX7
RFXANK
RFXAP
RGCC
RGL1
RGL2
RGL3
RGL4
RGMA
RGMB
RGP1
RGPD1
RGPD2

RGPD3
RGPD5
RGPD6
RGPD8
RGR
RGS1
RGS10
RGS12
RGS13
RGS14
RGS17
RGS18
RGS19
RGS2
RGS22
RGS3
RGS4
RGS5
RGS7BP
RGS9
RGSL1
RHBDD1
RHBDD2
RHBDD3
RHBDF1
RHBDF2
RHBDL2
RHBG
RHCG
RHD
RHEB
RHEBL1
RHEX
RHNO1
RHOA
RHOB
RHOBTB1
RHOBTB2
RHOBTB3
RHOC
RHOD
RHOF
RHOG
RHOH
RHOJ
RHOQ
RHOT1
RHOT2
RHOU
RHOV
RHOXF2B
RHPN1
RHPN2
RIBC1
RIBC2
RIC1
RIC3
RIC8A

RIC8B
RICTOR
RIDA
RIF1
RIIAD1
RILP
RILPL1
RILPL2
RIMBP3
RIMBP3C
RIMKLA
RIMKLB
RIMKLBP1
RIMS1
RIMS2
RIMS3
RIN1
RIN2
RIN3
RING1
RINL
RINT1
RIOK1
RIOK2
RIOK3
RIOX1
RIOX2
RIPK1
RIPK2
RIPK3
RIPK4
RIPOR1
RIPOR2
RIPOR3
RIPPLY3
RIT1
RITA1
RLF
RLIM
RLIMP1
RMC1
RMDN1
RMDN2
RMDN3
RMI1
RMI2
RMND1
RMND5A
RMND5B
RNASE1
RNASE10
RNASE4
RNASE6
RNASE7
RNASEH1
RNASEH2A
RNASEH2B
RNASEH2C

RNASEK
RNASEK-C17orf49
RNASEL
RNASET2
RND1
RND2
RND3
RNF10
RNF103
RNF103-CHMP3
RNF11
RNF111
RNF113A
RNF114
RNF115
RNF121
RNF122
RNF123
RNF125
RNF126
RNF13
RNF130
RNF135
RNF138
RNF139
RNF14
RNF141
RNF144A
RNF144B
RNF145
RNF146
RNF149
RNF150
RNF152
RNF157
RNF165
RNF166
RNF167
RNF168
RNF169
RNF170
RNF175
RNF180
RNF181
RNF183
RNF185
RNF187
RNF19A
RNF19B
RNF2
RNF20
RNF207
RNF212
RNF213
RNF214
RNF215
RNF216
RNF216P1

RNF217
RNF220
RNF222
RNF223
RNF227
RNF24
RNF25
RNF26
RNF31
RNF32
RNF34
RNF38
RNF39
RNF4
RNF40
RNF41
RNF43
RNF44
RNF5
RNF6
RNF7
RNF8
RNFT1
RNFT2
RNGTT
RNH1
RNLS
RNMT
RNPC3
RNPEP
RNPEPL1
RNPS1
RO60
ROBO1
ROBO2
ROCK1
ROCK1P1
ROCK2
ROGDI
ROMO1
ROPN1B
ROPN1L
ROR1
RORA
RORB
RORC
RP1
RP1L1
RP2
RP9
RP9P
RPA1
RPA2
RPA3
RPAIN
RPAP1
RPAP2
RPAP3

RPE
RPF1
RPF2
RPGR
RPGRIP1
RPGRIP1L
RPH3A
RPH3AL
RPIA
RPL10
RPL10A
RPL10P9
RPL11
RPL11P3
RPL12
RPL12P38
RPL13
RPL13A
RPL13AP5
RPL13P12
RPL13P5
RPL14
RPL15
RPL17
RPL17-C18orf32
RPL18
RPL18A
RPL18AP3
RPL19
RPL21
RPL21P119
RPL21P16
RPL22
RPL22L1
RPL22P1
RPL23
RPL23A
RPL23AP1
RPL23AP35
RPL23AP53
RPL23AP7
RPL23AP82
RPL24
RPL26
RPL26L1
RPL26P30
RPL27
RPL27A
RPL28
RPL29
RPL3
RPL30
RPL31
RPL32
RPL32P3
RPL34
RPL35
RPL35A

RPL36
RPL36A
RPL36A-HNRNPH2
RPL36AL
RPL37
RPL37A
RPL38
RPL39
RPL39L
RPL3L
RPL3P4
RPL3P6
RPL4
RPL41
RPL41P2
RPL4P7
RPL5
RPL5P30
RPL5P34
RPL5P4
RPL6
RPL6P27
RPL7
RPL7A
RPL7L1
RPL7P1
RPL7P18
RPL7P19
RPL7P41
RPL7P9
RPL8
RPL9
RPLP0
RPLP0P2
RPLP1
RPLP2
RPN1
RPN2
RPP14
RPP21
RPP25
RPP25L
RPP30
RPP38
RPRD1A
RPRD1B
RPRD2
RPRM
RPS10
RPS10-NUDT3
RPS10P7
RPS11
RPS12
RPS13
RPS14
RPS15
RPS15A
RPS16

RPS17
RPS18
RPS19
RPS19BP1
RPS2
RPS20
RPS20P22
RPS21
RPS23
RPS24
RPS25
RPS26
RPS27
RPS27A
RPS27L
RPS28
RPS29
RPS2P36
RPS2P5
RPS3
RPS3A
RPS3AP16
RPS3AP34
RPS4X
RPS4XP5
RPS4XP6
RPS4Y1
RPS5
RPS6
RPS6KA1
RPS6KA2
RPS6KA3
RPS6KA4
RPS6KA5
RPS6KA6
RPS6KB1
RPS6KB2
RPS6KC1
RPS6KL1
RPS6P20
RPS7
RPS8
RPS9
RPSA
RPSAP58
RPTN
RPTOR
RPUSD1
RPUSD2
RPUSD3
RPUSD4
RRAD
RRAGA
RRAGB
RRAGC
RRAGD
RRAS
RRAS2

RRBP1
RREB1
RRM1
RRM2
RRM2B
RRM2P3
RRN3
RRN3P1
RRN3P2
RRN3P3
RRN3P4
RRNAD1
RRP1
RRP12
RRP15
RRP1B
RRP36
RRP7A
RRP7BP
RRP8
RRP9
RRS1
RSAD1
RSAD2
RSBN1
RSBN1L
RSC1A1
RSF1
RSKR
RSL1D1
RSL24D1
RSPH1
RSPH10B
RSPH10B2
RSPH14
RSPH3
RSPH4A
RSPH9
RSPRY1
RSRC1
RSRC2
RSRP1
RSU1
RTCA
RTCB
RTEL1
RTEL1-TNFRSF6B
RTEL1P1
RTF1
RTF2
RTKN
RTKN2
RTL10
RTL5
RTL6
RTL8A
RTL8B
RTL8C

RTN1
RTN2
RTN3
RTN4
RTN4IP1
RTN4RL1
RTP4
RTRAF
RTTN
RUBCN
RUBCNL
RUFY1
RUFY2
RUFY3
RUFY4
RUNDC1
RUNDC3A
RUNX1
RUNX1T1
RUNX2
RUNX3
RUSC1
RUSC2
RUVBL1
RUVBL2
RWDD1
RWDD2A
RWDD2B
RWDD3
RWDD4
RXRA
RXRB
RXYLT1
RYBP
RYK
RYR1
RYR2
RYR3
S100A10
S100A11
S100A12
S100A13
S100A14
S100A16
S100A2
S100A4
S100A6
S100A7
S100A8
S100A9
S100B
S100P
S100PBP
S1PR3
S1PR4
SAA1
SAA2
SAA2-SAA4

SAA4
SAAL1
SACM1L
SACS
SAE1
SAFB
SAFB2
SAG
SALL2
SALL4
SALL4P7
SAMD1
SAMD10
SAMD12
SAMD14
SAMD15
SAMD3
SAMD4A
SAMD4B
SAMD5
SAMD8
SAMD9
SAMD9L
SAMHD1
SAMM50
SAMSN1
SAP130
SAP18
SAP25
SAP30
SAP30BP
SAP30L
SAPCD1
SAPCD2
SAR1A
SAR1B
SARAF
SARDH
SARM1
SARNP
SARS1
SARS2
SART1
SART3
SASH1
SASH3
SASS6
SAT1
SAT2
SATB1
SATB2
SAV1
SAXO2
SAYSD1
SBDS
SBDSP1
SBF1
SBF2

SBK1
SBNO1
SBNO2
SBSPON
SC5D
SCAF1
SCAF11
SCAF4
SCAF8
SCAI
SCAMP1
SCAMP2
SCAMP3
SCAMP4
SCAMP5
SCAND1
SCAND2P
SCAP
SCAPER
SCARA3
SCARB1
SCARB2
SCARF1
SCARF2
SCART1
SCCPDH
SCD
SCD5
SCEL
SCFD1
SCFD2
SCG3
SCGB1A1
SCGB2A1
SCGB2B2
SCGB3A1
SCGB3A2
SCHIP1
SCIMP
SCIN
SCLT1
SCLY
SCMH1
SCML1
SCML2
SCML4
SCN11A
SCN1A
SCN1B
SCN2A
SCN2B
SCN3A
SCN3B
SCN4B
SCN7A
SCN8A
SCN9A
SCNM1

SCNN1A
SCNN1B
SCNN1D
SCNN1G
SCO1
SCOC
SCP2
SCPEP1
SCRG1
SCRIB
SCRN1
SCRN2
SCRN3
SCUBE1
SCUBE2
SCUBE3
SCYL1
SCYL2
SCYL2P1
SCYL3
SDAD1
SDAD1P1
SDC1
SDC2
SDC3
SDC4
SDCBP
SDCBP2
SDCBPP1
SDCCAG8
SDE2
SDF2
SDF2L1
SDF4
SDHA
SDHAF1
SDHAF2
SDHAF3
SDHAF4
SDHAP1
SDHAP2
SDHB
SDHC
SDHD
SDK1
SDK2
SDR16C5
SDR39U1
SDR42E1
SDS
SDSL
SEC11A
SEC11C
SEC13
SEC14L1
SEC14L1P1
SEC14L2
SEC14L3

SEC14L4
SEC14L5
SEC14L6
SEC16A
SEC16B
SEC22A
SEC22B
SEC22B2P
SEC22C
SEC23A
SEC23B
SEC23IP
SEC24A
SEC24B
SEC24C
SEC24D
SEC31A
SEC31B
SEC61A1
SEC61A2
SEC61B
SEC61G
SEC62
SEC63
SEC63P1
SECISBP2
SECISBP2L
SECTM1
SEH1L
SEL1L
SEL1L3
SELENBP1
SELENOF
SELENOH
SELENOI
SELENOK
SELENOM
SELENON
SELENOO
SELENOP
SELENOS
SELENOT
SELENOW
SELL
SELP
SELPLG
SEM1
SEMA3A
SEMA3B
SEMA3C
SEMA3D
SEMA3E
SEMA3F
SEMA4A
SEMA4B
SEMA4C
SEMA4D
SEMA4F

SEMA4G
SEMA5A
SEMA6A
SEMA6B
SEMA6C
SEMA6D
SEMA7A
SEMG2
SENP1
SENP2
SENP3
SENP3-EIF4A1
SENP5
SENP6
SENP7
SENP8
SEPHS1
SEPHS2
SEPSECS
SEPTIN1
SEPTIN10
SEPTIN11
SEPTIN14
SEPTIN14P21
SEPTIN2
SEPTIN3
SEPTIN4
SEPTIN5
SEPTIN6
SEPTIN7
SEPTIN7P2
SEPTIN7P6
SEPTIN8
SEPTIN9
SERAC1
SERBP1
SERF1A
SERF1B
SERF2
SERGEF
SERHL2
SERINC1
SERINC2
SERINC3
SERINC4
SERINC5
SERP1
SERPINA1
SERPINA10
SERPINA2
SERPINA3
SERPINA9
SERPINB1
SERPINB10
SERPINB11
SERPINB13
SERPINB2
SERPINB3

SERPINB4
SERPINB5
SERPINB6
SERPINB7
SERPINB8
SERPINB9
SERPINE1
SERPINE2
SERPINF1
SERPING1
SERPINH1
SERPINI2
SERTAD1
SERTAD2
SERTAD3
SERTAD4
SESN1
SESN2
SESN3
SESTD1
SET
SETBP1
SETD1A
SETD1B
SETD2
SETD3
SETD4
SETD5
SETD6
SETD7
SETD9
SETDB1
SETDB2
SETMAR
SETX
SEZ6
SEZ6L2
SF1
SF3A1
SF3A2
SF3A3
SF3B1
SF3B2
SF3B3
SF3B4
SF3B5
SF3B6
SFI1
SFMBT1
SFMBT2
SFN
SFPQ
SFR1
SFSWAP
SFT2D1
SFT2D2
SFT2D3
SFTPB

SFXN1
SFXN2
SFXN3
SFXN4
SFXN5
SGCB
SGCD
SGF29
SGIP1
SGK1
SGK3
SGMS1
SGMS2
SGO1
SGO2
SGPL1
SGPP1
SGPP2
SGSH
SGSM1
SGSM2
SGSM3
SGTA
SGTB
SH2B1
SH2B2
SH2B3
SH2D1A
SH2D1B
SH2D2A
SH2D3A
SH2D3C
SH2D4A
SH2D6
SH3BGR
SH3BGRL
SH3BGRL2
SH3BGRL3
SH3BP1
SH3BP2
SH3BP4
SH3BP5
SH3BP5L
SH3D19
SH3D21
SH3GL1
SH3GL1P1
SH3GLB1
SH3GLB2
SH3KBP1
SH3PXD2A
SH3PXD2B
SH3RF1
SH3RF2
SH3RF3
SH3TC1
SH3TC2
SH3YL1

SHANK1
SHANK2
SHANK3
SHARPIN
SHB
SHC1
SHC3
SHC4
SHCBP1
SHE
SHF
SHFL
SHH
SHISA2
SHISA5
SHISA6
SHISA9
SHISAL2A
SHKBP1
SHLD1
SHLD2
SHLD2P1
SHLD2P2
SHLD2P3
SHLD3
SHMT1
SHMT2
SHOC1
SHOC2
SHOX
SHPK
SHPRH
SHQ1
SHQ1P1
SHROOM1
SHROOM2
SHROOM3
SHROOM4
SHTN1
SIAE
SIAH1
SIAH2
SIAH3
SIDT1
SIDT2
SIGIRR
SIGLEC1
SIGLEC10
SIGLEC11
SIGLEC14
SIGLEC15
SIGLEC5
SIGLEC6
SIGLEC7
SIGLEC8
SIGLEC9
SIGMAR1
SIK1

SIK1B
SIK2
SIK3
SIKE1
SIL1
SIM1
SIM2
SIMC1
SIN3A
SIN3B
SINHCAF
SIPA1
SIPA1L1
SIPA1L2
SIPA1L3
SIRPA
SIRPB1
SIRPB2
SIRPG
SIRT1
SIRT2
SIRT3
SIRT5
SIRT6
SIRT7
SIT1
SIVA1
SIX1
SIX2
SIX3
SIX4
SIX5
SKA1
SKA2
SKA3
SKAP1
SKAP2
SKI
SKIDA1
SKIL
SKINT1L
SKIV2L
SKP1
SKP2
SLA
SLA2
SLAIN1
SLAIN2
SLAMF1
SLAMF6
SLAMF7
SLAMF8
SLBP
SLC10A1
SLC10A3
SLC10A5
SLC10A7
SLC11A1

SLC11A2
SLC12A1
SLC12A2
SLC12A3
SLC12A4
SLC12A6
SLC12A7
SLC12A8
SLC12A9
SLC13A2
SLC13A3
SLC13A4
SLC13A5
SLC14A1
SLC14A2
SLC15A1
SLC15A2
SLC15A3
SLC15A4
SLC16A1
SLC16A10
SLC16A12
SLC16A13
SLC16A14
SLC16A2
SLC16A3
SLC16A4
SLC16A5
SLC16A6
SLC16A6P1
SLC16A7
SLC16A9
SLC17A5
SLC17A7
SLC17A9
SLC18A1
SLC18B1
SLC19A1
SLC19A2
SLC19A3
SLC1A1
SLC1A2
SLC1A3
SLC1A4
SLC1A5
SLC1A6
SLC20A1
SLC20A2
SLC22A1
SLC22A15
SLC22A16
SLC22A18
SLC22A20P
SLC22A23
SLC22A25
SLC22A4
SLC22A5
SLC22A9

SLC23A1
SLC23A2
SLC24A1
SLC24A2
SLC24A3
SLC24A4
SLC25A1
SLC25A10
SLC25A11
SLC25A12
SLC25A13
SLC25A14
SLC25A15
SLC25A16
SLC25A17
SLC25A19
SLC25A20
SLC25A21
SLC25A22
SLC25A23
SLC25A24
SLC25A25
SLC25A26
SLC25A27
SLC25A28
SLC25A29
SLC25A3
SLC25A30
SLC25A32
SLC25A33
SLC25A34
SLC25A35
SLC25A36
SLC25A37
SLC25A39
SLC25A4
SLC25A40
SLC25A42
SLC25A43
SLC25A44
SLC25A45
SLC25A46
SLC25A5
SLC25A51
SLC25A51P4
SLC25A53
SLC25A6
SLC26A1
SLC26A11
SLC26A2
SLC26A4
SLC26A6
SLC26A7
SLC26A8
SLC27A1
SLC27A2
SLC27A3
SLC27A4

SLC27A5
SLC28A2
SLC28A3
SLC29A1
SLC29A2
SLC29A4
SLC2A1
SLC2A10
SLC2A11
SLC2A12
SLC2A13
SLC2A14
SLC2A3
SLC2A3P1
SLC2A3P4
SLC2A4
SLC2A4RG
SLC2A5
SLC2A6
SLC2A8
SLC2A9
SLC30A1
SLC30A2
SLC30A3
SLC30A4
SLC30A5
SLC30A6
SLC30A7
SLC30A8
SLC30A9
SLC31A1
SLC31A2
SLC33A1
SLC34A2
SLC35A1
SLC35A2
SLC35A3
SLC35A4
SLC35A5
SLC35B1
SLC35B2
SLC35B4
SLC35C1
SLC35C2
SLC35D1
SLC35D2
SLC35E1
SLC35E2A
SLC35E2B
SLC35E3
SLC35E4
SLC35F2
SLC35F4
SLC35F5
SLC35F6
SLC35G1
SLC35G2
SLC35G5

SLC36A1
SLC36A2
SLC36A3
SLC36A4
SLC37A1
SLC37A2
SLC37A3
SLC37A4
SLC38A1
SLC38A10
SLC38A2
SLC38A4
SLC38A5
SLC38A6
SLC38A7
SLC38A9
SLC39A1
SLC39A10
SLC39A11
SLC39A13
SLC39A14
SLC39A3
SLC39A4
SLC39A6
SLC39A7
SLC39A8
SLC39A9
SLC3A1
SLC3A2
SLC40A1
SLC41A1
SLC41A2
SLC41A3
SLC43A2
SLC43A3
SLC44A1
SLC44A2
SLC44A3
SLC44A3-AS1
SLC44A4
SLC44A5
SLC45A3
SLC45A4
SLC46A1
SLC46A3
SLC47A1
SLC47A2
SLC48A1
SLC49A3
SLC49A4
SLC4A1
SLC4A10
SLC4A11
SLC4A1AP
SLC4A2
SLC4A4
SLC4A5
SLC4A7

SLC4A8
SLC50A1
SLC51A
SLC51B
SLC52A1
SLC52A2
SLC52A3
SLC5A1
SLC5A12
SLC5A2
SLC5A3
SLC5A5
SLC5A6
SLC5A8
SLC5A9
SLC66A1
SLC66A2
SLC66A3
SLC6A1
SLC6A13
SLC6A14
SLC6A16
SLC6A17
SLC6A20
SLC6A6
SLC6A8
SLC6A9
SLC7A1
SLC7A11
SLC7A14
SLC7A2
SLC7A4
SLC7A5
SLC7A6
SLC7A6OS
SLC7A7
SLC7A8
SLC7A9
SLC8A1
SLC8A2
SLC8B1
SLC9A1
SLC9A2
SLC9A3
SLC9A3R1
SLC9A3R2
SLC9A4
SLC9A5
SLC9A6
SLC9A7
SLC9A8
SLC9A9
SLC9B1
SLC9B2
SLC9C1
SLC9C2
SLCO1A2
SLCO1B3

SLCO2A1
SLCO2B1
SLCO3A1
SLCO4A1
SLCO4C1
SLCO5A1
SLF1
SLF2
SLFN11
SLFN12
SLFN12L
SLFN13
SLFN5
SLIRP
SLIT1
SLIT2
SLIT3
SLITRK2
SLITRK4
SLITRK5
SLK
SLMAP
SLPI
SLTM
SLU7
SLX1A
SLX1B
SLX4
SLX4IP
SMAD1
SMAD2
SMAD3
SMAD4
SMAD5
SMAD7
SMAD9
SMAGP
SMAP1
SMAP2
SMARCA1
SMARCA2
SMARCA4
SMARCA5
SMARCAD1
SMARCAL1
SMARCB1
SMARCC1
SMARCC2
SMARCD1
SMARCD2
SMARCD3
SMARCE1
SMBD1P
SMC1A
SMC2
SMC3
SMC3P1
SMC4

SMC5
SMC6
SMCHD1
SMCO4
SMCR8
SMDT1
SMG1
SMG1P1
SMG1P2
SMG1P3
SMG1P4
SMG1P5
SMG1P6
SMG1P7
SMG5
SMG6
SMG7
SMG8
SMG9
SMIM10L1
SMIM11A
SMIM11B
SMIM12
SMIM13
SMIM14
SMIM15
SMIM17
SMIM19
SMIM20
SMIM22
SMIM26
SMIM27
SMIM29
SMIM30
SMIM31
SMIM33
SMIM35
SMIM36
SMIM4
SMIM5
SMIM7
SMIM8
SMKR1
SMN1
SMN2
SMNDC1
SMO
SMOX
SMPD1
SMPD2
SMPD3
SMPD4
SMPD4BP
SMPD4P1
SMPDL3A
SMPDL3B
SMS
SMTN

SMTNL1
SMU1
SMUG1
SMURF1
SMURF2
SMYD2
SMYD3
SMYD4
SMYD5
SNAI1
SNAI2
SNAP23
SNAP29
SNAP47
SNAPC1
SNAPC2
SNAPC3
SNAPC4
SNAPC5
SNAPIN
SNCA
SNCAIP
SND1
SNED1
SNF8
SNHG28
SNHG32
SNIP1
SNN
SNRK
SNRNP200
SNRNP25
SNRNP27
SNRNP35
SNRNP40
SNRNP48
SNRNP70
SNRPA
SNRPA1
SNRPB
SNRPB2
SNRPC
SNRPD1
SNRPD2
SNRPD3
SNRPE
SNRPF
SNRPG
SNRPN
SNTA1
SNTB1
SNTB2
SNTN
SNU13
SNUPN
SNURF
SNW1
SNX1

SNX10
SNX11
SNX12
SNX13
SNX14
SNX15
SNX16
SNX17
SNX18
SNX18P3
SNX18P7
SNX19
SNX2
SNX20
SNX21
SNX22
SNX24
SNX25
SNX27
SNX29
SNX29P1
SNX29P2
SNX3
SNX30
SNX31
SNX32
SNX33
SNX4
SNX5
SNX6
SNX7
SNX8
SNX9
SOAT1
SOBP
SOCS1
SOCS2
SOCS3
SOCS4
SOCS5
SOCS6
SOCS7
SOD1
SOD2
SOGA1
SON
SORBS1
SORBS2
SORBS3
SORD
SORD2P
SORL1
SORT1
SOS1
SOS2
SOSTDC1
SOWAHB
SOWAHC

SOX11
SOX12
SOX13
SOX15
SOX2
SOX21
SOX4
SOX5
SOX6
SOX7
SOX9
SP1
SP100
SP110
SP140
SP140L
SP2
SP3
SP4
SP8
SPA17
SPACA6
SPACA7
SPACA9
SPAG1
SPAG16
SPAG17
SPAG4
SPAG5
SPAG6
SPAG7
SPAG8
SPAG9
SPARC
SPARCL1
SPART
SPAST
SPATA1
SPATA12
SPATA13
SPATA17
SPATA18
SPATA2
SPATA20
SPATA21
SPATA24
SPATA2L
SPATA31C1
SPATA32
SPATA33
SPATA4
SPATA5
SPATA5L1
SPATA6
SPATA6L
SPATA7
SPATS1
SPATS2

SPATS2L
SPC24
SPC25
SPCS1
SPCS2
SPCS2P4
SPCS3
SPDEF
SPDL1
SPDYA
SPDYE1
SPDYE10P
SPDYE16
SPDYE2
SPDYE2B
SPDYE3
SPDYE5
SPDYE6
SPDYE8
SPDYE9
SPECC1
SPECC1L
SPECC1L-ADORA2A
SPEF1
SPEF2
SPEG
SPEN
SPG11
SPG21
SPG7
SPHK1
SPHK2
SPI1
SPIB
SPICE1
SPIDR
SPIN1
SPIN2B
SPIN4
SPINDOC
SPINK5
SPINK7
SPINK9
SPINT1
SPINT2
SPIRE1
SPIRE2
SPN
SPNS1
SPNS2
SPOCD1
SPOCK1
SPOCK2
SPON1
SPON2
SPOP
SPOPL
SPOUT1

SPP1
SPP2
SPPL2A
SPPL2B
SPPL3
SPR
SPRED1
SPRED2
SPRED3
SPRR1B
SPRR2A
SPRR2D
SPRR2E
SPRR3
SPRTN
SPRY1
SPRY2
SPRY3
SPRY4
SPRYD3
SPRYD4
SPRYD7
SPSB1
SPSB2
SPSB3
SPTA1
SPTAN1
SPTB
SPTBN1
SPTBN2
SPTBN5
SPTLC1
SPTLC2
SPTLC3
SPTSSA
SPTSSB
SPTY2D1
SPTY2D1OS
SQLE
SQOR
SQSTM1
SRA1
SRARP
SRBD1
SRC
SRCAP
SRCIN1
SRD5A1
SRD5A2
SRD5A3
SREBF1
SREBF2
SREK1
SREK1IP1
SRF
SRFBP1
SRGAP1
SRGAP2

SRGAP2B
SRGAP2C
SRGAP2D
SRGAP3
SRGN
SRI
SRL
SRM
SRP14
SRP19
SRP54
SRP54-AS1
SRP68
SRP72
SRP72P2
SRP9
SRPK1
SRPK2
SRPK3
SRPRA
SRPRB
SRPX2
SRR
SRRD
SRRM1
SRRM1P3
SRRM2
SRRM4
SRRM5
SRRT
SRSF1
SRSF10
SRSF11
SRSF12
SRSF2
SRSF3
SRSF4
SRSF5
SRSF6
SRSF7
SRSF8
SRSF9
SRXN1
SS18
SS18L1
SS18L2
SSB
SSBP1
SSBP2
SSBP3
SSBP4
SSC5D
SSH1
SSH2
SSH3
SSNA1
SSPN
SSPO

SSR1
SSR2
SSR3
SSR4
SSRP1
SSTR2
SSU72
SSUH2
SSX2IP
ST13
ST14
ST18
ST20
ST20-MTHFS
ST3GAL1
ST3GAL2
ST3GAL3
ST3GAL4
ST3GAL5
ST3GAL6
ST6GAL1
ST6GAL2
ST6GALNAC1
ST6GALNAC2
ST6GALNAC3
ST6GALNAC4
ST6GALNAC5
ST6GALNAC6
ST7
ST7L
ST8SIA1
ST8SIA4
ST8SIA5
ST8SIA6
STAB1
STAC2
STAC3
STAG1
STAG2
STAG3
STAG3L1
STAG3L2
STAG3L3
STAG3L4
STAG3L5P
STAM
STAM2
STAMBP
STAMBPL1
STAP1
STAP2
STAR
STARD10
STARD13
STARD3
STARD3NL
STARD4
STARD5

STARD7
STARD8
STARD9
STAT1
STAT2
STAT3
STAT4
STAT5A
STAT5B
STAT6
STATH
STAU1
STAU2
STBD1
STC1
STEAP1
STEAP2
STEAP3
STEAP4
STIL
STIM1
STIM2
STIMATE
STIMATE-MUSTN1
STING1
STIP1
STK10
STK11
STK11IP
STK16
STK17A
STK17B
STK19
STK24
STK25
STK26
STK3
STK31
STK32C
STK33
STK35
STK36
STK38
STK38L
STK39
STK4
STK40
STMN1
STMN3
STMND1
STMP1
STN1
STOM
STOML1
STOML2
STOML3
STON1
STON1-GTF2A1L

STON2
STOX1
STOX2
STPG1
STPG2
STPG4
STRA6
STRADA
STRADB
STRAP
STRBP
STRC
STRIP1
STRIP2
STRN
STRN3
STRN4
STS
STT3A
STT3B
STUB1
STUM
STX10
STX11
STX12
STX16
STX16-NPEPL1
STX17
STX18
STX19
STX1A
STX1B
STX2
STX3
STX4
STX5
STX6
STX7
STX8
STXBP1
STXBP2
STXBP3
STXBP4
STXBP5
STXBP5L
STXBP6
STYK1
STYX
STYXL1
SUB1
SUCLA2
SUCLG1
SUCLG2
SUCLG2P4
SUCNR1
SUCO
SUDS3
SUFU

SUGP1
SUGP2
SUGT1
SUGT1P3
SULF1
SULF2
SULT1A1
SULT1A2
SULT1A3
SULT1A4
SULT1B1
SULT1C2
SULT1C2P1
SULT1C4
SULT1E1
SULT2A1
SULT2B1
SUMF1
SUMF2
SUMO1
SUMO2
SUMO3
SUN1
SUN2
SUOX
SUPT16H
SUPT20H
SUPT3H
SUPT4H1
SUPT5H
SUPT6H
SUPT7L
SUPV3L1
SURF1
SURF2
SURF4
SURF6
SUSD1
SUSD2
SUSD3
SUSD4
SUSD5
SUSD6
SUV39H1
SUV39H2
SUZ12
SUZ12P1
SV2A
SV2B
SV2C
SVBP
SVEP1
SVIL
SVIL-AS1
SVIP
SVOP
SVOPL
SWAP70

SWI5
SWSAP1
SWT1
SYAP1
SYBU
SYCP1
SYCP2
SYCP3
SYDE2
SYF2
SYK
SYMPK
SYN2
SYN3
SYNC
SYNCRIP
SYNE1
SYNE2
SYNE3
SYNE4
SYNGAP1
SYNGR1
SYNGR2
SYNJ1
SYNJ2
SYNJ2BP
SYNJ2BP-COX16
SYNM
SYNPO
SYNPO2
SYNPO2L
SYNRG
SYP
SYPL1
SYS1
SYS1-DBNDD2
SYT1
SYT11
SYT12
SYT13
SYT14
SYT15
SYT16
SYT17
SYT2
SYT5
SYT7
SYT8
SYTL1
SYTL2
SYTL3
SYTL4
SYTL5
SYVN1
SZRD1
SZT2
TAB1
TAB2

TAB3
TAC4
TACC1
TACC2
TACC3
TACO1
TACR2
TACSTD2
TADA1
TADA2A
TADA2B
TADA3
TAF1
TAF10
TAF11
TAF12
TAF13
TAF15
TAF1A
TAF1B
TAF1C
TAF1D
TAF2
TAF3
TAF4
TAF4B
TAF5
TAF5L
TAF6
TAF6L
TAF7
TAF8
TAF9
TAF9B
TAFA2
TAGAP
TAGLN
TAGLN2
TAGLN3
TAL1
TALDO1
TAMM41
TANC1
TANC2
TANGO2
TANGO6
TANK
TAOK1
TAOK2
TAOK3
TAP1
TAP2
TAPBP
TAPBPL
TAPT1
TARBP1
TARBP2
TARDBP

TARS1
TARS2
TARS3
TAS1R1
TAS2R14
TAS2R30
TAS2R4
TASOR
TASOR2
TASP1
TAT
TATDN1
TATDN1P1
TATDN2
TATDN2P2
TATDN3
TAX1BP1
TAX1BP3
TAZ
TBC1D1
TBC1D10A
TBC1D10B
TBC1D10C
TBC1D12
TBC1D13
TBC1D14
TBC1D15
TBC1D16
TBC1D17
TBC1D19
TBC1D2
TBC1D20
TBC1D22A
TBC1D22B
TBC1D23
TBC1D24
TBC1D25
TBC1D27P
TBC1D29P
TBC1D2B
TBC1D30
TBC1D31
TBC1D32
TBC1D3B
TBC1D3I
TBC1D3L
TBC1D4
TBC1D5
TBC1D7
TBC1D8
TBC1D8B
TBC1D9
TBC1D9B
TBCA
TBCB
TBCC
TBCCD1
TBCD

TBCEL
TBCK
TBK1
TBKBP1
TBL1X
TBL1XR1
TBL2
TBL3
TBP
TBPL1
TBR1
TBRG1
TBRG4
TBX1
TBX18
TBX19
TBX21
TBX6
TBXA2R
TBXAS1
TC2N
TCAF1
TCAF2
TCAIM
TCEA1
TCEA1P2
TCEA2
TCEA3
TCEAL1
TCEAL3
TCEAL4
TCEAL8
TCEAL9
TCEANC
TCEANC2
TCERG1
TCF12
TCF19
TCF20
TCF21
TCF23
TCF24
TCF25
TCF3
TCF4
TCF7
TCF7L1
TCF7L2
TCFL5
TCHH
TCHP
TCIM
TCIRG1
TCL1A
TCN1
TCN2
TCOF1
TCP1

TCP10L
TCP11
TCP11L1
TCP11L2
TCP11X1
TCP11X2
TCTA
TCTE1
TCTE3
TCTEX1D1
TCTEX1D2
TCTEX1D4
TCTN1
TCTN2
TCTN3
TDG
TDGF1
TDGF1P3
TDP1
TDP2
TDRD1
TDRD12
TDRD3
TDRD6
TDRD7
TDRD9
TDRKH
TDRP
TEAD1
TEAD3
TEC
TECPR1
TECPR2
TECR
TECRL
TECRP1
TEDC1
TEDDM1
TEF
TEFM
TEKT1
TEKT2
TEKT3
TEKT4
TEKT4P2
TELO2
TEN1
TEN1-CDK3
TENM1
TENM2
TENM4
TENT2
TENT4A
TENT4B
TENT5A
TENT5B
TENT5C
TEP1

TEPP
TEPSIN
TERB1
TERF1
TERF2
TERF2IP
TES
TESC
TESK1
TESK2
TESMIN
TESPA1
TET1
TET2
TET3
TEX10
TEX101
TEX14
TEX2
TEX26
TEX261
TEX264
TEX30
TEX35
TEX55
TEX9
TF
TFAM
TFAP2A
TFAP2C
TFAP4
TFB1M
TFB2M
TFCP2
TFCP2L1
TFDP1
TFDP2
TFE3
TFEB
TFEC
TFF3
TFG
TFIP11
TFPI
TFPT
TFR2
TFRC
TG
TGDS
TGFA
TGFB1
TGFB1I1
TGFB2
TGFB3
TGFBI
TGFBR1
TGFBR2
TGFBR3

TGFBRAP1
TGIF1
TGIF2
TGIF2-RAB5IF
TGM1
TGM2
TGM3
TGOLN2
TGS1
THADA
THAP1
THAP10
THAP11
THAP12
THAP12P7
THAP12P9
THAP2
THAP3
THAP4
THAP5
THAP6
THAP7
THAP8
THAP9
THBD
THBS1
THBS3
THBS4
THEM4
THEM6
THEMIS
THEMIS2
THG1L
THNSL1
THNSL2
THOC1
THOC2
THOC3
THOC5
THOC6
THOC7
THOP1
THRA
THRAP3
THRB
THSD1
THSD4
THSD7A
THTPA
THUMPD1
THUMPD2
THUMPD3
THYN1
TIA1
TIAF1
TIAL1
TIAM1
TIAM2

TICAM1
TICAM2
TICRR
TIE1
TIFA
TIFAB
TIGAR
TIGD1
TIGD2
TIGD4
TIGD5
TIGD6
TIGD7
TIGIT
TIMELESS
TIMM10
TIMM10B
TIMM13
TIMM17A
TIMM17B
TIMM21
TIMM22
TIMM23
TIMM23B
TIMM29
TIMM44
TIMM50
TIMM8B
TIMM9
TIMMDC1
TIMP1
TIMP2
TIMP3
TIMP4
TINAGL1
TINCR
TINF2
TIPARP
TIPIN
TIPRL
TIRAP
TJAP1
TJP1
TJP2
TJP3
TK1
TK2
TKFC
TKT
TKTL1
TLCD1
TLCD2
TLCD3A
TLCD3B
TLCD4
TLCD4-RWDD3
TLCD5
TLDC2

TLE1
TLE2
TLE3
TLE4
TLE5
TLK1
TLK2
TLK2P2
TLL2
TLN1
TLN2
TLNRD1
TLR1
TLR10
TLR2
TLR3
TLR4
TLR5
TLR6
TLR7
TLR8
TM2D1
TM2D2
TM2D3
TM4SF1
TM4SF20
TM6SF1
TM7SF2
TM7SF3
TM9SF1
TM9SF2
TM9SF3
TM9SF4
TMA16
TMA7
TMBIM1
TMBIM4
TMBIM6
TMC2
TMC3
TMC4
TMC5
TMC6
TMC7
TMC8
TMCC1
TMCC2
TMCC3
TMCO1
TMCO3
TMCO4
TMCO5A
TMCO5B
TMCO6
TMED1
TMED10
TMED2
TMED3

TMED4
TMED5
TMED7
TMED7-TICAM2
TMED8
TMED9
TMEM101
TMEM104
TMEM106A
TMEM106B
TMEM106C
TMEM107
TMEM108
TMEM109
TMEM11
TMEM115
TMEM116
TMEM117
TMEM119
TMEM120A
TMEM120B
TMEM121B
TMEM123
TMEM125
TMEM126A
TMEM126B
TMEM127
TMEM128
TMEM129
TMEM130
TMEM131
TMEM131L
TMEM132A
TMEM132B
TMEM134
TMEM135
TMEM138
TMEM139
TMEM140
TMEM141
TMEM143
TMEM144
TMEM145
TMEM147
TMEM14A
TMEM14B
TMEM14C
TMEM150A
TMEM150C
TMEM154
TMEM156
TMEM159
TMEM160
TMEM161A
TMEM161B
TMEM163
TMEM164
TMEM165

TMEM167A
TMEM167B
TMEM168
TMEM169
TMEM17
TMEM170A
TMEM170B
TMEM171
TMEM175
TMEM176A
TMEM176B
TMEM177
TMEM178A
TMEM178B
TMEM179B
TMEM18
TMEM181
TMEM182
TMEM183A
TMEM183B
TMEM184A
TMEM184B
TMEM184C
TMEM185A
TMEM185B
TMEM186
TMEM187
TMEM189
TMEM189-UBE2V1
TMEM19
TMEM190
TMEM191C
TMEM192
TMEM198B
TMEM199
TMEM200A
TMEM200B
TMEM200C
TMEM201
TMEM203
TMEM205
TMEM208
TMEM209
TMEM212
TMEM213
TMEM214
TMEM216
TMEM217
TMEM218
TMEM219
TMEM220
TMEM222
TMEM223
TMEM229B
TMEM230
TMEM231
TMEM231P1
TMEM232

TMEM234
TMEM236
TMEM237
TMEM238
TMEM238L
TMEM241
TMEM242
TMEM243
TMEM245
TMEM248
TMEM25
TMEM250
TMEM251
TMEM254
TMEM255A
TMEM255B
TMEM258
TMEM259
TMEM260
TMEM262
TMEM263
TMEM265
TMEM266
TMEM267
TMEM268
TMEM269
TMEM272
TMEM273
TMEM30A
TMEM30B
TMEM33
TMEM35A
TMEM35B
TMEM37
TMEM38A
TMEM38B
TMEM39A
TMEM39B
TMEM40
TMEM41A
TMEM41B
TMEM42
TMEM43
TMEM44
TMEM45A
TMEM45B
TMEM47
TMEM50A
TMEM50B
TMEM51
TMEM52
TMEM52B
TMEM53
TMEM54
TMEM59
TMEM60
TMEM61
TMEM62

TMEM63A
TMEM63B
TMEM63C
TMEM64
TMEM65
TMEM67
TMEM68
TMEM69
TMEM70
TMEM71
TMEM79
TMEM80
TMEM86A
TMEM87A
TMEM87B
TMEM88
TMEM8B
TMEM9
TMEM91
TMEM92
TMEM94
TMEM97
TMEM98
TMEM99
TMEM9B
TMF1
TMLHE
TMOD1
TMOD2
TMOD3
TMPO
TMPPE
TMPRSS11A
TMPRSS11B
TMPRSS11BNL
TMPRSS11D
TMPRSS11E
TMPRSS12
TMPRSS13
TMPRSS2
TMPRSS3
TMPRSS4
TMPRSS7
TMSB10
TMSB15B
TMSB4X
TMTC1
TMTC2
TMTC3
TMTC4
TMUB1
TMUB2
TMX1
TMX2
TMX2P1
TMX3
TMX4
TNC

TNF
TNFAIP1
TNFAIP2
TNFAIP3
TNFAIP6
TNFAIP8
TNFAIP8L1
TNFAIP8L2
TNFAIP8L3
TNFRSF10A
TNFRSF10B
TNFRSF10C
TNFRSF10D
TNFRSF11A
TNFRSF11B
TNFRSF12A
TNFRSF13B
TNFRSF13C
TNFRSF14
TNFRSF19
TNFRSF1A
TNFRSF1B
TNFRSF21
TNFRSF25
TNFRSF6B
TNFRSF8
TNFRSF9
TNFSF10
TNFSF12
TNFSF12-TNFSF13
TNFSF13
TNFSF13B
TNFSF14
TNFSF15
TNFSF4
TNFSF8
TNFSF9
TNIK
TNIP1
TNIP2
TNIP3
TNK1
TNK2
TNKS
TNKS1BP1
TNKS2
TNNC1
TNNI1
TNNI2
TNNI3
TNNT2
TNNT3
TNPO1
TNPO2
TNPO3
TNR
TNRC18
TNRC6A

TNRC6B
TNRC6C
TNS1
TNS2
TNS3
TNS4
TNXB
TOB1
TOB2
TOE1
TOGARAM1
TOGARAM2
TOLLIP
TOM1
TOM1L1
TOM1L2
TOMM20
TOMM22
TOMM34
TOMM40
TOMM40L
TOMM5
TOMM6
TOMM7
TOMM70
TONSL
TOP1
TOP1MT
TOP2A
TOP2B
TOP3A
TOP3B
TOPBP1
TOPORS
TOR1A
TOR1AIP1
TOR1AIP2
TOR1B
TOR2A
TOR3A
TOR4A
TOX
TOX2
TOX3
TOX4
TOX4P1
TP53
TP53AIP1
TP53BP1
TP53BP2
TP53I11
TP53I13
TP53I3
TP53INP1
TP53INP2
TP53RK
TP63
TP73

TP73-AS1
TPBG
TPCN1
TPCN2
TPD52
TPD52L1
TPD52L2
TPGS1
TPGS2
TPH1
TPH2
TPI1
TPI1P2
TPK1
TPM1
TPM2
TPM3
TPM3P9
TPM4
TPMT
TPO
TPP1
TPP2
TPPP
TPPP3
TPR
TPRA1
TPRG1
TPRG1L
TPRKB
TPRN
TPRXL
TPSAB1
TPSB2
TPST1
TPST2
TPT1
TPTE2P2
TPTE2P5
TPTE2P6
TPTEP1
TPTEP2
TPX2
TRA2A
TRA2B
TRABD
TRABD2A
TRAC
TRADD
TRAF1
TRAF2
TRAF3
TRAF3IP1
TRAF3IP2
TRAF3IP3
TRAF4
TRAF5
TRAF6

TRAF7
TRAFD1
TRAIP
TRAK1
TRAK2
TRAM1
TRAM2
TRANK1
TRAP1
TRAPPC1
TRAPPC10
TRAPPC11
TRAPPC12
TRAPPC13
TRAPPC2
TRAPPC2B
TRAPPC2L
TRAPPC3
TRAPPC3L
TRAPPC4
TRAPPC5
TRAPPC6A
TRAPPC6B
TRAPPC8
TRAPPC9
TRAT1
TRBC1
TRBC2
TRDC
TRDMT1
TREM1
TREM2
TREML1
TREML2
TREML3P
TREML4
TRERF1
TREX1
TRGC1
TRGC2
TRHDE
TRIAP1
TRIB1
TRIB2
TRIB3
TRIM11
TRIM13
TRIM14
TRIM16
TRIM16L
TRIM2
TRIM21
TRIM22
TRIM23
TRIM24
TRIM25
TRIM26
TRIM27

TRIM28
TRIM29
TRIM3
TRIM31
TRIM32
TRIM33
TRIM34
TRIM35
TRIM36
TRIM37
TRIM38
TRIM39
TRIM4
TRIM41
TRIM44
TRIM45
TRIM46
TRIM47
TRIM5
TRIM52
TRIM55
TRIM56
TRIM58
TRIM59
TRIM6
TRIM61
TRIM62
TRIM65
TRIM66
TRIM68
TRIM69
TRIM7
TRIM71
TRIM72
TRIM73
TRIM8
TRIM9
TRIO
TRIOBP
TRIP10
TRIP11
TRIP12
TRIP13
TRIP4
TRIP6
TRIQK
TRIR
TRIT1
TRMO
TRMT1
TRMT10A
TRMT10B
TRMT10C
TRMT11
TRMT112
TRMT12
TRMT13
TRMT1L

TRMT2A
TRMT2B
TRMT44
TRMT5
TRMT6
TRMT61A
TRMT61B
TRMT9B
TRMU
TRNAU1AP
TRNP1
TRNT1
TRO
TROAP
TRPC1
TRPC3
TRPC4AP
TRPC5
TRPM2
TRPM3
TRPM4
TRPM6
TRPM7
TRPM8
TRPS1
TRPT1
TRPV1
TRPV2
TRPV3
TRPV4
TRPV6
TRRAP
TRUB1
TRUB2
TSC1
TSC2
TSC22D1
TSC22D2
TSC22D3
TSC22D4
TSEN15
TSEN2
TSEN34
TSEN54
TSFM
TSG101
TSGA10
TSHR
TSHZ1
TSHZ2
TSHZ3
TSKU
TSN
TSNARE1
TSNAX
TSNAXIP1
TSPAN1
TSPAN10

TSPAN11
TSPAN12
TSPAN13
TSPAN14
TSPAN15
TSPAN17
TSPAN19
TSPAN2
TSPAN3
TSPAN31
TSPAN33
TSPAN5
TSPAN6
TSPAN7
TSPAN8
TSPAN9
TSPO
TSPOAP1
TSPY26P
TSPYL1
TSPYL2
TSPYL4
TSPYL5
TSR1
TSR2
TSR3
TSSC2
TSSC4
TST
TSTA3
TSTD1
TSTD2
TSTD3
TTBK1
TTBK2
TTC1
TTC12
TTC13
TTC14
TTC16
TTC17
TTC19
TTC21A
TTC21B
TTC22
TTC23
TTC23L
TTC25
TTC26
TTC27
TTC28
TTC29
TTC3
TTC30A
TTC30B
TTC31
TTC32
TTC33

TTC34
TTC37
TTC38
TTC39A
TTC39B
TTC39C
TTC3P1
TTC4
TTC41P
TTC5
TTC6
TTC7A
TTC7B
TTC8
TTC9
TTC9C
TTF1
TTF2
TTI1
TTI2
TTK
TTL
TTLL1
TTLL10
TTLL11
TTLL12
TTLL13P
TTLL3
TTLL4
TTLL5
TTLL6
TTLL7
TTLL9
TTN
TTPA
TTPAL
TTYH1
TTYH2
TTYH3
TUB
TUBA1A
TUBA1B
TUBA1C
TUBA4A
TUBA4B
TUBA8
TUBB
TUBB2A
TUBB2B
TUBB2BP1
TUBB3
TUBB4B
TUBB6
TUBBP5
TUBD1
TUBE1
TUBG1
TUBG2

TUBGCP2
TUBGCP3
TUBGCP4
TUBGCP5
TUBGCP6
TUFM
TUFT1
TULP2
TULP3
TULP4
TUNAR
TUSC1
TUSC2
TUSC3
TUT1
TUT4
TUT7
TVP23A
TVP23B
TVP23C
TVP23C-CDRT4
TWF1
TWF2
TWISTNB
TWNK
TWSG1
TXK
TXLNA
TXLNB
TXLNG
TXLNGY
TXN
TXN2
TXNDC11
TXNDC12
TXNDC15
TXNDC16
TXNDC17
TXNDC2
TXNDC5
TXNDC8
TXNDC9
TXNIP
TXNL1
TXNL4A
TXNL4B
TXNRD1
TXNRD2
TXNRD3
TYK2
TYMP
TYMS
TYR
TYRO3
TYROBP
TYSND1
TYW1
TYW1B

TYW3
TYW5
U2AF1
U2AF1L4
U2AF1L5
U2AF2
U2SURP
UACA
UAP1
UAP1L1
UBA1
UBA2
UBA3
UBA5
UBA52
UBA6
UBA7
UBAC1
UBAC2
UBALD1
UBALD2
UBAP1
UBAP1L
UBAP2
UBAP2L
UBASH3A
UBASH3B
UBB
UBBP4
UBC
UBD
UBE2A
UBE2B
UBE2D1
UBE2D2
UBE2D3
UBE2D4
UBE2E1
UBE2E2
UBE2E3
UBE2F
UBE2G1
UBE2G2
UBE2H
UBE2I
UBE2J1
UBE2J2
UBE2K
UBE2L3
UBE2L6
UBE2M
UBE2N
UBE2O
UBE2Q1
UBE2Q2
UBE2Q2P1
UBE2Q2P2
UBE2R2

UBE2S
UBE2V1
UBE2V2
UBE2W
UBE2Z
UBE3A
UBE3B
UBE3C
UBE3D
UBE4A
UBE4B
UBFD1
UBIAD1
UBL3
UBL4A
UBL5
UBL7
UBLCP1
UBN1
UBN2
UBOX5
UBP1
UBQLN1
UBQLN2
UBQLN4
UBQLNL
UBR1
UBR2
UBR3
UBR4
UBR5
UBR7
UBTD1
UBTD2
UBTF
UBXN1
UBXN10
UBXN11
UBXN2A
UBXN2B
UBXN4
UBXN6
UBXN7
UBXN8
UCHL1
UCHL3
UCHL5
UCK1
UCK2
UCKL1
UCN3
UCP2
UCP3
UEVLD
UFC1
UFD1
UFL1
UFM1

UFSP2
UGCG
UGDH
UGGT1
UGGT2
UGP2
UGT1A1
UGT1A10
UGT1A6
UGT1A7
UGT2A1
UGT2A2
UGT3A1
UGT8
UHMK1
UHRF1
UHRF1BP1
UHRF1BP1L
UHRF2
UHRF2P1
UIMC1
ULBP1
ULBP3
ULK1
ULK2
ULK3
ULK4
UMAD1
UMODL1
UMPS
UNC119
UNC119B
UNC13A
UNC13B
UNC13C
UNC13D
UNC45A
UNC50
UNC5B
UNC5C
UNC79
UNC80
UNC93B1
UNG
UNK
UNKL
UPB1
UPF1
UPF2
UPF3A
UPF3B
UPK1B
UPK3B
UPK3BL1
UPP1
UPP2
UPRT
UQCC1

UQCC2
UQCC3
UQCR10
UQCR11
UQCRB
UQCRC1
UQCRC2
UQCRFS1
UQCRFS1P1
UQCRH
UQCRHL
UQCRQ
URB1
URB2
URGCP
URGCP-MRPS24
URI1
URM1
UROD
UROS
USB1
USE1
USF1
USF2
USF3
USH1C
USH1G
USH2A
USHBP1
USO1
USP1
USP10
USP11
USP12
USP13
USP14
USP15
USP16
USP17L6P
USP18
USP19
USP2
USP20
USP21
USP22
USP24
USP25
USP27X
USP28
USP3
USP30
USP31
USP32
USP32P1
USP32P2
USP33
USP34
USP35

USP36
USP37
USP38
USP39
USP4
USP40
USP41
USP42
USP43
USP44
USP45
USP46
USP47
USP48
USP49
USP5
USP51
USP53
USP54
USP6
USP6NL
USP7
USP8
USP9X
USP9Y
USPL1
UST
UTP11
UTP14A
UTP14C
UTP15
UTP18
UTP20
UTP23
UTP25
UTP3
UTP4
UTP6
UTRN
UTS2
UTS2B
UTY
UVRAG
UVSSA
UXS1
UXT
VAC14
VAMP1
VAMP2
VAMP3
VAMP4
VAMP5
VAMP7
VAMP8
VANGL1
VANGL2
VAPA
VAPB

VARS1
VARS2
VASH1
VASH2
VASN
VASP
VAT1
VAV1
VAV2
VAV3
VBP1
VCAM1
VCAN
VCL
VCP
VCPIP1
VCPKMT
VDAC1
VDAC1P8
VDAC2
VDAC3
VDR
VEGFA
VEGFB
VEGFC
VENTX
VENTXP4
VEPH1
VEZF1
VEZT
VGLL1
VGLL3
VGLL4
VHL
VIL1
VILL
VIM
VIPAS39
VIPR1
VIRMA
VKORC1
VKORC1L1
VLDLR
VMA21
VMAC
VMO1
VMP1
VN1R21P
VN1R40P
VNN1
VNN2
VNN3
VOPP1
VPS11
VPS13A
VPS13B
VPS13C
VPS13D

VPS16
VPS18
VPS25
VPS26A
VPS26B
VPS26C
VPS28
VPS29
VPS33A
VPS33B
VPS35
VPS35L
VPS36
VPS37A
VPS37B
VPS37C
VPS39
VPS41
VPS45
VPS4A
VPS4B
VPS50
VPS51
VPS52
VPS53
VPS54
VPS72
VPS8
VPS9D1
VRK1
VRK2
VRK3
VSIG1
VSIG10
VSIG10L
VSIG2
VSIG4
VSIR
VSNL1
VSTM2A
VSTM2L
VSTM4
VSX1
VTA1
VTCN1
VTI1A
VTI1B
VWA1
VWA2
VWA3A
VWA3B
VWA5A
VWA5B1
VWA7
VWA8
VWC2
VWCE
VWDE

VWF
VXN
WAC
WAPL
WARS1
WARS2
WAS
WASF1
WASF2
WASF3
WASH2P
WASH3P
WASH4P
WASH5P
WASH6P
WASH7P
WASH8P
WASH9P
WASHC1
WASHC2A
WASHC2C
WASHC3
WASHC4
WASHC5
WASL
WBP1
WBP11
WBP1L
WBP2
WBP2NL
WBP4
WDCP
WDFY1
WDFY2
WDFY3
WDFY4
WDHD1
WDPCP
WDR1
WDR11
WDR12
WDR13
WDR17
WDR18
WDR19
WDR20
WDR24
WDR25
WDR26
WDR27
WDR3
WDR31
WDR33
WDR34
WDR35
WDR36
WDR37
WDR38

WDR4
WDR41
WDR43
WDR44
WDR45
WDR45B
WDR45P1
WDR46
WDR47
WDR48
WDR49
WDR5
WDR53
WDR54
WDR55
WDR59
WDR5B
WDR6
WDR60
WDR61
WDR62
WDR63
WDR66
WDR7
WDR70
WDR72
WDR73
WDR74
WDR75
WDR76
WDR77
WDR78
WDR81
WDR82
WDR83
WDR83OS
WDR86
WDR87
WDR89
WDR90
WDR91
WDR92
WDR93
WDR97
WDSUB1
WDTC1
WDYHV1
WEE1
WEE2
WFDC1
WFDC11
WFDC2
WFDC21P
WFDC3
WFDC6
WFS1
WHAMM
WHAMMP2

WHAMMP3
WHRN
WIPF1
WIPF2
WIPF3
WIPI1
WIPI2
WIZ
WLS
WNK1
WNK2
WNK3
WNK4
WNT2B
WNT4
WNT5A
WNT5B
WNT7A
WNT7B
WNT9A
WNT9B
WRAP53
WRAP73
WRN
WRNIP1
WSB1
WSB2
WSCD2
WTAP
WTAPP1
WTIP
WWC1
WWC2
WWC3
WWOX
WWP1
WWP2
WWTR1
XAB2
XAF1
XBP1
XDH
XG
XIAP
XIAPP2
XIAPP3
XK
XKR4
XKR5
XKR6
XKR8
XKR9
XKRX
XPA
XPC
XPNPEP1
XPNPEP3
XPO1

XPO4
XPO5
XPO6
XPO7
XPOT
XPR1
XRCC1
XRCC2
XRCC3
XRCC4
XRCC5
XRCC6
XRN1
XRN2
XRRA1
XXYLT1
XYLB
XYLT1
XYLT2
YAE1
YAF2
YAP1
YARS1
YARS2
YBEY
YBX1
YBX1P1
YBX3
YDJC
YEATS2
YEATS4
YES1
YES1P1
YIF1A
YIF1B
YIPF1
YIPF2
YIPF3
YIPF4
YIPF5
YIPF6
YJEFN3
YJU2
YKT6
YLPM1
YME1L1
YOD1
YPEL1
YPEL2
YPEL3
YPEL5
YRDC
YTHDC1
YTHDC2
YTHDF1
YTHDF2
YTHDF3
YWHAB

YWHAE
YWHAEP1
YWHAG
YWHAH
YWHAQ
YWHAQP5
YWHAZ
YY1
YY1AP1
YY2
Z82195.2
Z95152.1
Z97055.1
Z97634.1
Z98755.1
ZACN
ZADH2
ZAP70
ZBBX
ZBED1
ZBED2
ZBED3
ZBED4
ZBED5
ZBED6
ZBED6CL
ZBED8
ZBP1
ZBTB1
ZBTB10
ZBTB11
ZBTB14
ZBTB16
ZBTB17
ZBTB18
ZBTB2
ZBTB20
ZBTB21
ZBTB22
ZBTB24
ZBTB25
ZBTB26
ZBTB3
ZBTB33
ZBTB34
ZBTB37
ZBTB38
ZBTB39
ZBTB4
ZBTB40
ZBTB41
ZBTB42
ZBTB43
ZBTB44
ZBTB45
ZBTB45P1
ZBTB46
ZBTB47

ZBTB48
ZBTB49
ZBTB5
ZBTB6
ZBTB7A
ZBTB7B
ZBTB7C
ZBTB8A
ZBTB8B
ZBTB8OS
ZBTB9
ZC2HC1A
ZC2HC1C
ZC3H10
ZC3H11A
ZC3H12A
ZC3H12B
ZC3H12C
ZC3H12D
ZC3H13
ZC3H14
ZC3H15
ZC3H18
ZC3H3
ZC3H4
ZC3H6
ZC3H7A
ZC3H7B
ZC3H8
ZC3HAV1
ZC3HAV1L
ZC3HC1
ZC4H2
ZCCHC10
ZCCHC14
ZCCHC17
ZCCHC2
ZCCHC24
ZCCHC3
ZCCHC4
ZCCHC7
ZCCHC8
ZCCHC9
ZCRB1
ZCWPW1
ZCWPW2
ZDHHC1
ZDHHC11
ZDHHC11B
ZDHHC12
ZDHHC13
ZDHHC14
ZDHHC15
ZDHHC16
ZDHHC17
ZDHHC18
ZDHHC2
ZDHHC20

ZDHHC21
ZDHHC23
ZDHHC24
ZDHHC3
ZDHHC4
ZDHHC4P1
ZDHHC5
ZDHHC6
ZDHHC7
ZDHHC8
ZDHHC9
ZEB1
ZEB2
ZER1
ZFAND1
ZFAND2A
ZFAND2B
ZFAND3
ZFAND4
ZFAND5
ZFAND6
ZFAT
ZFC3H1
ZFHX2
ZFHX3
ZFHX4
ZFP1
ZFP14
ZFP2
ZFP28
ZFP3
ZFP30
ZFP36
ZFP36L1
ZFP36L2
ZFP37
ZFP41
ZFP42
ZFP62
ZFP64
ZFP69
ZFP69B
ZFP82
ZFP90
ZFP91
ZFP91-CNTF
ZFP92
ZFPL1
ZFPM1
ZFPM2
ZFR
ZFR2
ZFX
ZFY
ZFYVE1
ZFYVE16
ZFYVE19
ZFYVE21

ZFYVE26
ZFYVE27
ZFYVE28
ZFYVE9
ZG16
ZG16B
ZGPAT
ZGRF1
ZHX1
ZHX1-C8orf76
ZHX2
ZHX3
ZIC3
ZIK1
ZIK1P1
ZKSCAN1
ZKSCAN2
ZKSCAN3
ZKSCAN4
ZKSCAN5
ZKSCAN7
ZKSCAN8
ZMAT1
ZMAT2
ZMAT3
ZMAT5
ZMIZ1
ZMIZ2
ZMPSTE24
ZMYM1
ZMYM2
ZMYM3
ZMYM4
ZMYM5
ZMYM6
ZMYND10
ZMYND11
ZMYND12
ZMYND15
ZMYND19
ZMYND8
ZNF10
ZNF100
ZNF101
ZNF106
ZNF107
ZNF112
ZNF114
ZNF117
ZNF12
ZNF121
ZNF124
ZNF131
ZNF132
ZNF133
ZNF134
ZNF135
ZNF136

ZNF137P
ZNF138
ZNF14
ZNF140
ZNF141
ZNF142
ZNF143
ZNF146
ZNF148
ZNF154
ZNF155
ZNF157
ZNF16
ZNF160
ZNF165
ZNF169
ZNF17
ZNF174
ZNF175
ZNF177
ZNF18
ZNF180
ZNF181
ZNF182
ZNF184
ZNF185
ZNF189
ZNF19
ZNF195
ZNF197
ZNF2
ZNF20
ZNF200
ZNF202
ZNF204P
ZNF205
ZNF207
ZNF208
ZNF211
ZNF212
ZNF213
ZNF214
ZNF215
ZNF217
ZNF219
ZNF22
ZNF221
ZNF222
ZNF223
ZNF224
ZNF225
ZNF226
ZNF227
ZNF229
ZNF23
ZNF230
ZNF232
ZNF233

ZNF234
ZNF235
ZNF236
ZNF239
ZNF24
ZNF248
ZNF25
ZNF250
ZNF251
ZNF252P
ZNF253
ZNF254
ZNF256
ZNF257
ZNF26
ZNF260
ZNF263
ZNF264
ZNF266
ZNF267
ZNF268
ZNF271P
ZNF273
ZNF274
ZNF275
ZNF276
ZNF277
ZNF28
ZNF280B
ZNF280C
ZNF280D
ZNF281
ZNF282
ZNF283
ZNF284
ZNF285
ZNF286A
ZNF286B
ZNF287
ZNF292
ZNF296
ZNF3
ZNF30
ZNF300
ZNF300P1
ZNF302
ZNF304
ZNF311
ZNF316
ZNF317
ZNF318
ZNF319
ZNF32
ZNF320
ZNF321P
ZNF322
ZNF322P1
ZNF324

ZNF324B
ZNF326
ZNF329
ZNF330
ZNF331
ZNF333
ZNF334
ZNF335
ZNF337
ZNF33A
ZNF33B
ZNF34
ZNF341
ZNF343
ZNF345
ZNF346
ZNF347
ZNF35
ZNF350
ZNF354A
ZNF354B
ZNF354C
ZNF358
ZNF362
ZNF365
ZNF366
ZNF367
ZNF37A
ZNF37BP
ZNF382
ZNF383
ZNF384
ZNF385A
ZNF385C
ZNF385D
ZNF391
ZNF394
ZNF395
ZNF396
ZNF397
ZNF398
ZNF404
ZNF407
ZNF408
ZNF41
ZNF410
ZNF414
ZNF415
ZNF415P1
ZNF416
ZNF417
ZNF418
ZNF419
ZNF420
ZNF425
ZNF426
ZNF428
ZNF429

ZNF43
ZNF430
ZNF431
ZNF432
ZNF433
ZNF436
ZNF438
ZNF439
ZNF44
ZNF440
ZNF441
ZNF442
ZNF443
ZNF444
ZNF445
ZNF446
ZNF449
ZNF45
ZNF451
ZNF454
ZNF460
ZNF461
ZNF462
ZNF467
ZNF468
ZNF469
ZNF470
ZNF471
ZNF473
ZNF474
ZNF48
ZNF480
ZNF483
ZNF484
ZNF485
ZNF486
ZNF487
ZNF490
ZNF491
ZNF492
ZNF493
ZNF496
ZNF497
ZNF500
ZNF501
ZNF502
ZNF503
ZNF506
ZNF507
ZNF510
ZNF511
ZNF512
ZNF512B
ZNF513
ZNF514
ZNF516
ZNF517
ZNF518A

ZNF518B
ZNF519
ZNF519P1
ZNF524
ZNF525
ZNF526
ZNF527
ZNF528
ZNF529
ZNF530
ZNF532
ZNF540
ZNF542P
ZNF543
ZNF544
ZNF546
ZNF547
ZNF548
ZNF549
ZNF550
ZNF551
ZNF552
ZNF554
ZNF555
ZNF556
ZNF557
ZNF558
ZNF559
ZNF559-ZNF177
ZNF56
ZNF561
ZNF562
ZNF563
ZNF564
ZNF565
ZNF566
ZNF567
ZNF568
ZNF569
ZNF57
ZNF570
ZNF571
ZNF572
ZNF573
ZNF574
ZNF576
ZNF577
ZNF578
ZNF579
ZNF580
ZNF581
ZNF582
ZNF583
ZNF584
ZNF585A
ZNF585B
ZNF586
ZNF587

ZNF587B
ZNF589
ZNF592
ZNF594
ZNF595
ZNF596
ZNF597
ZNF598
ZNF599
ZNF600
ZNF605
ZNF606
ZNF607
ZNF608
ZNF609
ZNF610
ZNF611
ZNF613
ZNF614
ZNF615
ZNF616
ZNF618
ZNF619
ZNF620
ZNF621
ZNF622
ZNF623
ZNF624
ZNF625
ZNF625-ZNF20
ZNF626
ZNF627
ZNF628
ZNF629
ZNF630
ZNF638
ZNF639
ZNF641
ZNF644
ZNF646
ZNF648
ZNF649
ZNF652
ZNF653
ZNF654
ZNF655
ZNF658
ZNF658B
ZNF66
ZNF660
ZNF662
ZNF663P
ZNF664
ZNF665
ZNF667
ZNF668
ZNF669
ZNF670

ZNF670-ZNF695
ZNF671
ZNF672
ZNF674
ZNF675
ZNF677
ZNF678
ZNF680
ZNF680P1
ZNF681
ZNF682
ZNF683
ZNF684
ZNF687
ZNF688
ZNF689
ZNF69
ZNF691
ZNF692
ZNF695
ZNF696
ZNF697
ZNF699
ZNF7
ZNF70
ZNF700
ZNF701
ZNF702P
ZNF703
ZNF704
ZNF706
ZNF707
ZNF708
ZNF709
ZNF71
ZNF710
ZNF711
ZNF713
ZNF714
ZNF716
ZNF717
ZNF718
ZNF720
ZNF721
ZNF724
ZNF726
ZNF727
ZNF736
ZNF737
ZNF738
ZNF74
ZNF740
ZNF746
ZNF747
ZNF749
ZNF750
ZNF75A
ZNF75D

ZNF76
ZNF761
ZNF763
ZNF764
ZNF765
ZNF766
ZNF767P
ZNF768
ZNF77
ZNF770
ZNF771
ZNF772
ZNF773
ZNF774
ZNF775
ZNF776
ZNF777
ZNF778
ZNF780A
ZNF780B
ZNF781
ZNF782
ZNF783
ZNF784
ZNF785
ZNF786
ZNF787
ZNF788P
ZNF789
ZNF79
ZNF790
ZNF791
ZNF792
ZNF793
ZNF799
ZNF8
ZNF80
ZNF800
ZNF805
ZNF808
ZNF81
ZNF812P
ZNF813
ZNF814
ZNF816
ZNF816-ZNF321P
ZNF821
ZNF823
ZNF826P
ZNF827
ZNF829
ZNF83
ZNF830
ZNF831
ZNF833P
ZNF835
ZNF836
ZNF837

ZNF839
ZNF84
ZNF841
ZNF843
ZNF844
ZNF845
ZNF846
ZNF85
ZNF850
ZNF852
ZNF853
ZNF859P
ZNF860
ZNF862
ZNF865
ZNF875
ZNF876P
ZNF879
ZNF880
ZNF891
ZNF90
ZNF90P1
ZNF91
ZNF92
ZNF93
ZNF969P
ZNF99
ZNFX1
ZNHIT1
ZNHIT2
ZNHIT3
ZNHIT6
ZNRD1
ZNRD1ASP
ZNRD2
ZNRF1
ZNRF2
ZNRF2P2
ZNRF3
ZP3
ZPBP2
ZPLD2P
ZPR1
ZRANB1
ZRANB2
ZRANB3
ZRSR2
ZSCAN12
ZSCAN12P1
ZSCAN16
ZSCAN18
ZSCAN2
ZSCAN20
ZSCAN21
ZSCAN22
ZSCAN25
ZSCAN26
ZSCAN29

ZSCAN30
ZSCAN31
ZSCAN32
ZSCAN4
ZSCAN5A
ZSCAN9
ZSWIM1
ZSWIM3
ZSWIM4
ZSWIM5
ZSWIM6
ZSWIM7
ZSWIM8
ZSWIM9
ZUP1
ZW10
ZWILCH
ZWINT
ZXDA
ZXDB
ZXDC
ZYG11A
ZYG11B
ZYX
ZZEF1
