# Supplementary material for: Identification of Novel COVID-19 Biomarkers by Multiple Feature Selection Strategies
Source: Comput Math Methods Med. 2021 Sep 27;2021:2203636. doi: 10.1155/2021/2203636 (PMC8485143; doi:10.1155/2021/2203636)
Supplement: Supplementary 2 — Supplementary Table 2: screened top 500 feature genes after the mRMR method. [file 2203636.f2.pdf]

Supplementary Table S2 Screened top 500 feature genes after mRMR sequencing

Order	Name
1	OAS2
2	RPLP0
3	IGFBP2
4	ZMYND10
5	CLSTN1
6	XAF1
7	PRDX5
8	RPL15
9	KRT8
10	HERC6
11	C9orf24
12	RPS7
13	MAGED2
14	CMPK2
15	EEF2
16	ATP5IF1
17	VAPA
18	IFI44L
19	CAPS
20	OAZ1
21	TAGLN2
22	EPCAM
23	RPLP1
24	SPINT2
25	RRAD
26	RPL10A
27	CLU
28	RPL3
29	TUBB2A
30	RPL30
31	STOML3
32	CKB
33	OAS3
34	RPL13
35	TUBB4B
36	PTTG1IP
37	RPS28
38	C11orf88
39	RPL4
40	CRIP1
41	ST6GALNAC2
42	TRIM22
43	RPS18
44	CCDC33
45	UBE2L6
46	RPS8
47	SLC44A4
48	GPX4
49	CD99
50	RPS5
51	STAT1
52	LGALS3
53	GNAS
54	CD59
55	RPL18
56	DDX60
57	RPL32

58	ACTR1B
59	EZR
60	RPS3A
61	RHOB
62	KRT10
63	CTSH
64	RPL35
65	C9orf116
66	IGFBP5
67	SLC256A
68	CITED2
69	TUFM
70	RPL26
71	TMEM59
72	ARL3
73	PTPRN2
74	CHCHD2
75	ALDH3A1
76	CCN1
77	RPS14
78	TTC29
79	FBXW9
80	PARP9
81	RPL13A
82	COPS6
83	CBR1
84	EEF1G
85	GBP4
86	SLC25A25
87	FAM166B
88	NDUFA4
89	WDR13
90	RPS10
91	SAMD9
92	ERBB2
93	EEF1D
94	CD24
95	RPL21
96	RIPK4
97	ANAPC11
98	CIB1
99	ARL4A
100	APOL6
101	PIGR
102	RPL34
103	TPT1
104	ELMO3
105	COPS9
106	STEAP3
107	ACO2
108	IFI16
109	FAU
110	DGCR6
111	COMMD6
112	ABHD2
113	TRAF4
114	RPS9
115	RBM3

116	PKIG
117	EDF1
118	EPHX1
119	HSPBP1
120	DTX3L
121	RPS13
122	ELF3
123	CES1
124	RPL37
125	C9orf135
126	ITGB4
127	FAM183A
128	EIF2AK2
129	CALM1
130	NDUFV1
131	JUN
132	NDUFB10
133	TMEM205
134	RPL27A
135	FDXR
136	TPPP3
137	EPSTI1
138	RPS29
139	CD9
140	RPLP2
141	AGR2
142	FIS1
143	FAM174A
144	ITGB5
145	GCHFR
146	MAPK8IP1
147	RPS27
148	SLFN5
149	RPN1
150	LAMTOR4
151	MB
152	CIRBP
153	RPL18A
154	COQ4
155	C20orf85
156	RPS17
157	H2AJ
158	UQCR11
159	IGFBP7
160	FAM166A
161	RPL36A
162	TCTEX1D2
163	KIAA2013
164	C11orf97
165	DAG1
166	MYL6
167	SMDT1
168	ANKRD65
169	GDF15
170	LRP11
171	ZNFX1
172	CDC34
173	ATP5F1E

174	HNRNPF
175	GNA11
176	RPS21
177	ZNF703
178	TRIM28
179	SSR4
180	NDUFB2
181	KRT19
182	UQCRQ
183	PPP2R1A
184	DVL1
185	RPL17-C18orf32
186	MS4A6A
187	TMEM141
188	NDUFB7
189	COX6A1
190	CELSR1
191	ROMO1
192	CCDC153
193	S100A6
194	ARF5
195	CYB561
196	CTSB
197	RPL7
198	MID1IP1
199	CXXC5
200	GSN
201	RUVBL2
202	GADD45A
203	RPL8
204	TMEM9
205	RPL7A
206	ST14
207	TIMM13
208	NACA
209	DDR1
210	COMT
211	POLR2L
212	DPY30
213	CD82
214	TRIM5
215	LRRC23
216	ATAD3A
217	TMA7
218	AL928654.3
219	KLF4
220	SLPI
221	ARL6IP4
222	H3-3A
223	NOP53
224	NDUFA2
225	TACSTD2
226	RBMS2
227	RPS19
228	ZDHHC1
229	CFAP53
230	SIX2
231	SELENOH

232	RPL19
233	CDKN2AIP
234	RPS6
235	LRRC46
236	OAS1
237	HINT1
238	HAGH
239	PLPPR3
240	JTB
241	ERGIC3
242	MRPS31
243	GUK1
244	ITPA
245	PERP
246	UBXN6
247	RPS15A
248	CCDC74B
249	PFKP
250	ALOX15
251	GTPBP6
252	RHBDD2
253	RPL29
254	STUB1
255	PGRMC1
256	AKT1
257	PTPRF
258	UQCRC1
259	C22orf15
260	C12orf57
261	ATP6V0B
262	DHCR24
263	AC011295.1
264	RPS2
265	EIF3CL
266	RGL2
267	RPS3
268	LRRC75A
269	MVP
270	SPAG7
271	RPL24
272	TADA3
273	P4HB
274	PPP1R7
275	SLC2A1
276	RPS15
277	TBCB
278	EEF1B2
279	BLOC1S1
280	PYCR2
281	SUN1
282	MBTPS1
283	POLR2J
284	ASL
285	SMIM22
286	PLK2
287	TLE5
288	ATP5ME
289	CCDC78

290	GPX3
291	BSG
292	AKR1A1
293	RPS24
294	CFAP73
295	NDUFC1
296	TUBGCP2
297	EIF3K
298	C5orf49
299	IMPA2
300	DEGS2
301	RPL41
302	NIBAN2
303	LRRC43
304	LARP6
305	GADD45GIP1
306	XRN1
307	EFNA1
308	MRPL14
309	RPL10
310	CD151
311	PROSER1
312	RSPH9
313	DDT
314	SSBP4
315	CHCHD6
316	SGSM3
317	SNRPD2
318	SELENOS
319	RPL6
320	POLR2I
321	CLDN4
322	GSTA2
323	ERP29
324	CCDC96
325	RPL17
326	SNRPB
327	RBP1
328	UBXN11
329	ARPC1A
330	DDOST
331	PRKCSH
332	RPS16
333	SRI
334	NME2
335	IFT43
336	VPS51
337	SELENBP1
338	PSMA7
339	NDUFAB1
340	CYB5A
341	CLDN3
342	UBXN1
343	UBL5
344	LMNA
345	EIF4H
346	NDUFA13
347	ST6GALNAC6

348	DNAI2
349	LRWD1
350	ANAPC16
351	APBB1
352	COX5B
353	POR
354	CAPN1
355	ROGDI
356	CTTN
357	MIF
358	KRT17
359	TRAK1
360	EPHA2
361	LRRC10B
362	C12orf75
363	WDR1
364	OGFOD2
365	GSTA1
366	MMP24OS
367	CLDN7
368	KRT5
369	PRR29
370	NGRN
371	ALKBH7
372	RSPH1
373	GTF3C5
374	YIF1B
375	SNX17
376	MORN5
377	R3HCC1
378	RPS20
379	KRT15
380	DNPH1
381	PUF60
382	EIF3C
383	MPG
384	GAS2L2
385	S100A13
386	MRPL23
387	SYTL1
388	CHCHD1
389	SCRN2
390	PHACTR4
391	ECHS1
392	NENF
393	COX4I1
394	MRPL28
395	CSRP1
396	TOMM7
397	ZNF562
398	ECRG4
399	CFAP298
400	GADD45B
401	ENKD1
402	NQO1
403	FKBP8
404	TMED1
405	GET3

406	PEPD
407	RPL23A
408	SGTA
409	LMAN2
410	RPL35A
411	CUL7
412	MRPS34
413	RPL27
414	ROPN1L
415	AAMP
416	SPOUT1
417	AKR7A2
418	BAIAP2
419	RPL39
420	WDR54
421	RPL36
422	TSPAN15
423	RPL12
424	PSMG3
425	INO80D
426	VILL
427	RPSA
428	CDK9
429	SURF2
430	SEPTIN9
431	IFT57
432	AHCY
433	DPCD
434	RPS10-NUDT3
435	GPAA1
436	GON7
437	SIVA1
438	DYNLRB2
439	SEC61B
440	MICOS10
441	ADIRF
442	ELOB
443	NDUFAF3
444	PWWP2B
445	SERTAD1
446	TCEA2
447	SOD1
448	CSTB
449	DHX30
450	PCYT2
451	RABGAP1L
452	ILF3
453	PPIA
454	CCDC88C
455	TAB3
456	NDUFS6
457	UPF1
458	LONP1
459	DUS1L
460	RPS12
461	FAM92B
462	NUMA1
463	REL

464	KEAP1
465	AURKAIP1
466	TFF3
467	HMGN3
468	UBAC1
469	DNAJB2
470	RPS25
471	TST
472	FXYD3
473	CCDC74A
474	MRPL55
475	AQP5
476	DNAJB1
477	TRAF7
478	PFDN5
479	HSPA1B
480	DYNLL1
481	CTSD
482	PRPF31
483	C16orf71
484	HMGN2
485	NUDC
486	PHPT1
487	THAP7
488	RNH1
489	GPS1
490	PRMT2
491	TSTD1
492	PRPF6
493	AC011448.1
494	SERF2
495	KRT18
496	PEBP1
497	SOX2
498	ALDH3B1
499	BCR
500	GPX1
