# Supplementary material for: Identification of Novel COVID-19 Biomarkers by Multiple Feature Selection Strategies
Source: Comput Math Methods Med. 2021 Sep 27;2021:2203636. doi: 10.1155/2021/2203636 (PMC8485143; doi:10.1155/2021/2203636)
Supplement: Supplementary 3 — Supplementary Table 3: screened top 66 feature genes by the IFS method. [file 2203636.f3.pdf]

Supplementary Table S3 Screened top 66 feature genes by IFS method

Order	Name
1	OAS2
2	RPLP0
3	IGFBP2
4	ZMYND10
5	CLSTN1
6	XAF1
7	PRDX5
8	RPL15
9	KRT8
10	HERC6
11	C9orf24
12	RPS7
13	MAGED2
14	CMPK2
15	EEF2
16	ATP5IF1
17	VAPA
18	IFI44L
19	CAPS
20	OAZ1
21	TAGLN2
22	EPCAM
23	RPLP1
24	SPINT2
25	RRAD
26	RPL10A
27	CLU
28	RPL3
29	TUBB2A
30	RPL30
31	STOML3
32	CKB
33	OAS3
34	RPL13
35	TUBB4B
36	PTTG1IP
37	RPS28
38	C11orf88
39	RPL4
40	CRIP1
41	ST6GALNAC2
42	TRIM22
43	RPS18
44	CCDC33
45	UBE2L6
46	RPS8
47	SLC44A4
48	GPX4
49	CD99
50	RPS5
51	STAT1
52	LGALS3
53	GNAS
54	CD59
55	RPL18
56	DDX60
57	RPL32

58	ACTR1B
59	EZR
60	RPS3A
61	RHOB
62	KRT10
63	CTSH
64	RPL35
65	C9orf116
66	IGFBP5
